# Transcriptome Screening and Identification of Chemosensory Genes in the Goji Berry Psyllid, *Bactericera gobica* (Hemiptera: Psyllidae)

**DOI:** 10.3390/biology14081105

**Published:** 2025-08-21

**Authors:** Zhanghui Liu, Yang Ge, Zekun Zhang, Jiayi Liang, Chuanzhi Kang, Chengcai Zhang, Kang Chen, Xiufu Wan, Liu Zhang, Wangpeng Shi, Honghao Chen

**Affiliations:** 1 Institute of Plant Protection, Ningxia Academy of Agriculture and Forestry Sciences, Yinchuan 750002, China; 15879774115@163.com; 2 Department of Entomology and MOA Key Lab of Pest Monitoring and Green Management, College of Plant Protection, China Agricultural University, Beijing 100193, China; 3 State Key Laboratory for Quality Ensurance and Sustainable Use of Dao-di Herbs, National Resource Center for Chinese Materia Medica, China Academy of Chinese Medical Sciences, Beijing 100700, China; 4 Institute of Plant Inspection and Quarantine, Chinese Academy of Quality and Inspection & Testing, Beijing 100176, China

**Keywords:** psyllid, transcriptome, sensorial perception, olfactory, gustation, chemosensory mechanism, *Bactericera gobica*

## Abstract

Goji berry is widely consumed worldwide and holds substantial market value, yet its cultivation faces significant threats from the goji berry psyllid (*Bactericera gobica*). Chemosensory-related genes play critical roles in regulating insect behaviors, which makes them key molecular targets for the development of environmentally friendly pest control strategies. However, chemosensory genes in *B. gobica* have not been previously identified or characterized. In this study, we sequenced transcriptomes from the antennae and body tissues of male and female *B. gobica* and annotated genes associated with chemosensory functions. We identified 15 odorant-binding proteins (OBPs), 18 chemosensory proteins (CSPs), 3 sensory neuron membrane proteins (SNMPs), 26 odorant receptors (ORs), 8 gustatory receptors (GRs), and 32 ionotropic receptors (IRs). Transcriptome data and a quantitative real-time PCR confirmed the tissue-specific expression patterns of these genes, with several genes, including three *BgobOBPs*, eight *BgobCSPs*, one *BgobOR*, two *BgobGRs*, and two *BgobIR*, highly expressed in the antennae, suggesting their role in olfactory recognition. *BgobGR1* was most highly expressed among GRs, indicating its important role in gustatory perception. We also identified gene *BgobGR5* with differential expression patterns between females and males. Our study represents the first characterization of chemosensory genes in a *Bactericera* species.

## Simple Summary

Goji berries are now widely consumed across the globe with a huge market value. The pest goji berry psyllid (*Bactericera gobica*) represents one of the most significant threats to their cultivation. Insects use their chemosensory system to detect and discriminate among a wide array of chemical cues in their environment. This study identifies chemosensory genes in the psyllid’s antennae and body tissues and conducts phylogenetic analyses to confirm gene annotations and infer potential functions. We also perform a quantitative analysis to measure the expression patterns of these chemosensory genes, particularly those linked to antennae-biased and sex-biased expression. Our results will facilitate the targeted identification of attractants and repellents by focusing on these olfactory genes, offering a sustainable solution to control this pest. Our findings pave the way for developing environmentally friendly and targeted methods to protect goji berry crops.

## 1. Introduction

Insects have evolved a highly specialized chemosensory system composed of complex and sensitive olfactory and gustatory subsystems [1,2,3]. These systems enable insects to detect and discriminate a wide array of chemical cues in their environment, facilitating essential behaviors such as foraging, mating, oviposition site selection, and predator avoidance [4,5,6]. Generally, the olfactory system is involved in the long-range detection of volatile molecules, which helps individuals locate hosts or mates, and the gustatory system plays a crucial role in short-range recognition, particularly in the adaptation of herbivorous species to their hosts [7]. Chemosensory organs, including the antennae, mouthparts, legs, wings, and reproductive structures, are involved in both olfaction and gustation. Olfactory sensilla detect volatile compounds such as host plant odors, pheromones, carboxylic acids, amines, and humidity changes [8,9,10,11,12,13,14,15,16], whereas gustatory sensilla respond to non-volatile substances such as sugars, bitter compounds, salts, and water [17].

These sensilla house olfactory receptor neurons (ORNs) and gustatory sensory neurons, where lipophilic odorants enter the sensillar lymph through cuticular pores and bind with odorant-binding proteins (OBPs) or chemosensory proteins (CSPs). These binding proteins then transport odorants to olfactory receptors (ORs), which modulate the membrane potential of ORNs, generating electrical signals that initiate downstream behavioral responses [18,19]. OBPs have also been shown to interact with sensory neuron membrane proteins (SNMPs) and play additional roles in chemosensation [20]. Gustatory receptors (GRs), in turn, are essential for detecting sugars, amino acids, salts, and water, as well as for the rejection of bitter compounds in herbivores [17]. Ionotropic receptors (IRs) comprise another major chemosensory receptor family; these differ structurally from ORs and GRs but serve complementary functions in both olfactory and gustatory processes [21,22,23]. Genes encoding these chemosensory-related proteins are key to insect chemical communication, and disruptions in their expression or function can interfere with mating, host localization, and feeding, thereby reducing herbivore damage and providing potential avenues for pest control. These genes thus provide important molecular targets for behavior-based pest management strategies.

Psyllids (Psylloidea) are a group of hemipteran herbivores and include several economically significant pests such as the Asian citrus psyllid (*Diaphorina citri*) and the potato psyllid (*Bactericera cockerelli*). Goji berries (*Lycium* spp.) are globally significant health foods, supplements, and traditional medicines; it is reported that the output of dried wolfberry in China alone reached about 250,000 tons, with an export volume of USD 120 million, and the output value of the wolfberry industry exceeded USD 4 billion [24]. However, goji berry cultivation is severely threatened by the goji berry psyllid *Bactericera gobica*, which causes significant economic losses by extracting sap, deforming leaves, causing fruit and leaf drop, and promoting sooty mold through honeydew excretion [25]. The high fecundity of *B. gobica* further exacerbates its threat to goji production. The chemosensory mechanisms of psyllids, including *B. gobica*, at the molecular level remain poorly understood, and this has hindered the development of effective detection and control strategies.

To address this knowledge gap, we employed high-throughput transcriptome sequencing to systematically identify chemosensory-related genes in *B. gobica*, including OBPs, CSPs, SNMPs, ORs, GRs, and IRs. Phylogenetic analyses were performed using homologous genes from other hemipteran and model insect species, as well as the *Plodia interpunctella,* to verify gene identification, classify them into specific subfamilies or clades, and infer potential functions based on their evolutionary relationships. In addition, a quantitative real-time PCR was conducted to examine the tissue- and sex-specific expression of candidate genes. The results increase the number of chemosensory-related genes identified in *B. gobica* and lay the groundwork for future functional studies and the development of targeted pest management strategies.

## 2. Materials and Methods

### 2.1. Insect Rearing and Tissue Collection

The experimental goji berry psyllid population was originated from field collections in Yinchuan, China and then reared for multiple generations in laboratory nylon mesh cages (60 cm × 60 cm × 60 cm) at 25 ± 2 °C with a 16L: 8D photoperiod, and the same colony as we described in our previous study [26]. Potted goji berry seedlings were provided as food and oviposition substrates. Adult *B. gobica* were collected using an aspirator and anesthetized with CO_2_ for sex discrimination based on the appearance of their abdominal segments. Under a stereomicroscope, antennae were collected from 24–72 h newly emerged adult male and female psyllids, with 400 pairs per sex separately. Because of the small RNA yield per antenna, pooling 400 pairs of antennae per replicate was unavoidable to obtain sufficient RNA. From these individuals, the heads without antennae were dissected as head samples, and the decapitated bodies were used as body samples for each sex. Additionally, ten whole female and male psyllids were collected as whole-body samples. All samples were placed in RNase-free 1.5 mL centrifuge tubes, with three biological replicates for each sample type. All samples were immediately frozen in liquid nitrogen and stored at −80 °C until RNA extraction.

### 2.2. RNA Etraction, cDNA Library Construction, Sequencing, Assembly, and Annotation

Total RNA was extracted from antennae, heads, bodies, and whole-body psyllids tissues using the Total RNA Extraction Kit (Jianshi Bio, Bejing, China). To prevent RNA degradation during dissection, forceps were sterilized using an autoclave. The dissection table and stereomicroscope were disinfected with 75% ethanol. Lab coats, disposable masks, and gloves were worn during the procedure. All dissections were performed on ice. Antennae were immediately transferred into RNase-free tubes placed in liquid nitrogen and stored at −80 °C until RNA extraction. RNA concentration was measured with a spectrophotometer (Nanodrop: Thermo Fisher Scientific, Waltham, MA, USA), and integrity was verified by agarose gel electrophoresis. Qualified RNA samples were sent to Novogene Co., Ltd. (Beijing, China) for cDNA library construction and sequencing. Antennae, head, and body samples from male and female psyllids were sequenced on the Illumina NovaSeq X Plus-PE 150 platform (second-generation sequencing: Illumina, Inc., San Diego, CA, USA). To obtain longer reads, whole-body samples were sequenced on the PacBio Sequel platform (long-read sequencing: Pacific Biosciences, Menlo Park, CA, USA). The obtained sequences underwent a quality check (SMRT Link V8.0), assembly, and clustering by Novogene Co., Ltd. (Beijing, China).

For Illumina sequencing data from antennae, head, and body tissues, raw reads were cleaned by removing adapters and low-quality bases. GC content and Q30 values were calculated to assess sequence quality. Clean reads were assembled de novo using Trinity 2.6.6 to generate transcripts. For the whole-body transcriptome data from PacBio Sequel II Sequencing, the sequencing process begins with polymerase reads generated during SMRT sequencing, which are processed to yield subreads representing continuous long reads from individual ZMWs (zero mode waveguide). These subreads are then aligned and consensus-called to produce circular consensus sequences (CCS), which are classified as either full-length reads (FL) containing both 5’ and 3’ primers or non-full-length reads (NFL) missing either primer. The FL reads undergo further processing to remove artificial concatemers and chimeric artifacts, resulting in full-length non-chimeric sequences (FLNC). These FLNC sequences are used to generate initial consensus sequences, which are subsequently polished using the Arrow algorithm for improved accuracy. The polished consensus sequences then undergo hybrid error correction with LoRDEC V0.7 software utilizing second-generation sequencing data, followed by sequence deduplication using CD-HIT v4.6.8 software to cluster similar sequences and produce a final non-redundant dataset. The clustered sequences were used as references for downstream analyses. All Illumina- and PacBio-assembled transcripts were annotated by BLAST 2.9.0 alignment against seven major biological databases, NR, NT, KO, SwissProt, PFAM, GO, and KOGs, for gene function annotation.

### 2.3. Identification of Chemosensory Gene Families and Phylogenetic Analysis

Candidate chemosensory genes were identified by searching the *B. gobica* transcriptome annotation files using chemosensory gene-related keywords (“OBP”, “odorant”, “odorant-binding”, “odorant-binding protein”, “CSP”, ”chemosensory”, “chemosensory protein”, “OR”, “Olfactory receptor”, “Olfactory “, “odorant receptor”, “IR”, “Ionotropic receptor”, “Ionotropic”, “GR”, “gustatory receptor”, “gustatory”, “SNMP”, “sensory neuron membrane protein”), followed by BLAST 2.9.0 searches with a screening threshold of e-value < 10^−5^. We did not apply an FPKM cut-off in our analysis, as we were concerned that potentially meaningful lowly expressed genes (e.g., FPKM < 5) might be inadvertently excluded. ORFs were predicted using ORF Finder, and the resulting protein sequences were verified by alignment against the NCBI database. More chemosensory genes were detected in the Illumina transcriptomes from different tissues, which formed the primary basis for our analysis. The PacBio dataset, derived from whole-body samples, was used to supplement Illumina data and verify sequence accuracy. We found that the ORF sequences were consistent between platforms. A small number of chemosensory genes were identified only in the PacBio data, and these were also included in our results. Signal peptides of OBPs, CSPs, and SNMPs were predicted using SignalP 6.0. The molecular weight, isoelectric point, hydrophilicity index, and instability coefficient of OBPs, CSPs, and SNMPs were predicted using the ExPASy 3.0 Proteomics Server. To support gene annotation and infer evolutionary relationships, we performed phylogenetic analyses on sequences from *B. gobica* and other Hemipteran insects and model species. Transmembrane domains of ORs, IRs, and GRs were predicted using the TMHMM Server v.2.0. For the phylogenetic analysis, maximum likelihood trees were constructed based on amino acid sequences of candidate chemosensory genes from *B. gobica*, and other insect species using IQ-TREE v3.0.1 and visualized using iTOL v7.0. Eight psyllid and other Hemipteran species (*Diaphorina citri, Aphis gossypii, Myzus persicae*, *Aphis craccivora*, *Bemisia tabaci*, *Halyomorpha halys*, *Planococcus citri*, *Rhopalosiphum padi*), as well as two model insect species (*Drosophila melanogaster* and *Bombyx mori*), and *Plodia interpunctella* were used for the phylogenetic analysis.

### 2.4. Transcript Abundance Analysis and qPCR Validation

Based on the transcriptome data, TPM (Transcripts Per Million) and FPKM (Fragments Per Kilobase of transcript per Million mapped reads) values for all chemosensory genes were obtained, and differential expression profiles among different tissues were analyzed after log_2_(*n* + 1) transformation of the TPM values [27]. RNA samples extracted from the antennae, heads, and bodies samples of male and female psyllids were treated with PrimeScript™ RT Reagent Kit (Takara Bio Inc., Kusatsu, Japan) with gDNA Eraser (Perfect Real Time) to remove genomic DNA and reverse-transcribed into cDNA. The cDNA was diluted five-fold and used as a template for qPCR. *β-Actin* and *Gapdh* genes have been proven to be suitable reference genes in *B. gobica* [28], and the expression levels of these were stable across different tissues of *B. gobica* (Supplementary Figure S5). These two reference genes were used as internal reference genes for qPCR validation to confirm the transcriptome results and characterize gene expression patterns in male and female antennae, heads, and bodies. Due to sample limitations, we prioritized a subset of genes for qRT-PCR validation, focusing on those with antenna-enriched expression or putative functional importance. A real-time qPCR was performed in a 20 μL reaction system under the following conditions: initial denaturation: 95 °C for 30 s (1 cycle); amplification: 95 °C for 5 s and 60 °C for 30 s (40 cycles); melting curve analysis: 95 °C for 5 s and 60 °C for 30 s (1 cycle), then increase temperature from 65 °C to 95 °C at a rate of 0.5 °C/s with continuous fluorescence acquisition. Relative expression levels were calculated using the 2^^(−ΔΔCt)^ method. To determine the significance of differences among groups, we performed the Shapiro–Wilk test to assess normality and Levene’s test to assess homogeneity of variance. qPCR data that conformed to a normal distribution with homogeneous variance were analyzed using a one-way ANOVA with Duncan’s post-hoc test, while the remaining data were analyzed using the non-parametric Kruskal–Wallis test in SPSS 22.0 software. Data were not transformed. Data were presented as mean ± standard error. A significance threshold was set at *p* < 0.05. All experiments were conducted with three independent biological replicates, each with three technical replicates. Only genes showing a significant difference in the transcriptome data (|log_2_(FoldChange)| ≥ 5 and *p* ≤ 0.05) and a significant difference in the qPCR results (*p* ≤ 0.05) were classified as sex-differentially expressed genes.

## 3. Results

### 3.1. Transcriptome Sequencing and Assembly

A total of eighteen libraries were generated through Illumina sequencing: three biological replicates of *B. gobica* antennae from 400 females and three from 400 males, plus three replicates each for female heads, male heads, female bodies, and male bodies (using 50 individuals per replicate) with an average depth of 98.81×. An average of 21,900,530 clean reads were obtained per sample, and 96.72% of the reads had a Phred quality score of Q20 or greater. Over 90% of the bases had a quality score greater than Q30 (Supplementary Table S1). The de novo assembly of the clean reads yielded 108,102 transcripts. After removing redundant reads and isoforms, a total of 63,823 unigenes were obtained, with an average length of 1061 bp and an N50 of 1612 bp (Supplementary Tables S2 and S3).

Additionally, a full-length transcriptome library of *B. gobica* adult females and males was constructed using the PacBio platform. This yielded 730,803 polymerase reads with an average length of 120,320 bp and a HiFi read coverage depth of 26.57×. Sequences were further clustered, and redundancies were removed using CD-HIT v4.6.8 software through sequence alignment, which yielded 17,290 transcript sequences with an average length of 2626 bp and an N50 of 2907 (Supplementary Tables S4 and S5).

Among the antennal transcriptome data, a total of 30,216 unigenes (47.34%) were successfully annotated in at least one database. Specifically, 22,871 unigenes (35.83%) were annotated in the NR database (Supplementary Table S6). In the whole-body transcriptome dataset, a total of 16,171 transcript sequences (93.5%) were annotated in at least one public database, including 15,887 (91.89%) in the NR database (Figure S1). A total of 38% of the genes in the *B. gobica* transcriptome showed significant sequence similarity to *Diaphorina citri*. The four species with the highest percentages of BLAST 2.9.0 matches were *D. citri* (38.3%), *Myzus persicae* (27%), *Bemisia tabaci* (3.4%), and *Cryptotermes secundus* (2.4%) (Figure S2).

### 3.2. Mining of Chemosensory Genes

Based on the transcriptome annotations, we used ORFfinder (https://www.ncbi.nlm.nih.gov/orffinder/, accessed on 15 September 2022) to predict open reading frames (ORFs) in the transcripts. Protein-coding sequences were identified through a domain-based Blastp analysis, followed by the removal of redundant sequences. A total of 102 transcripts were identified as encoding proteins homologous to six major chemosensory gene families. These included 15, 18, 3, 26, 8, and 32 transcripts encoding *BgobOBPs*, *BgobCSPs*, *BgobSNMPs*, *BgobORs*, *BgobGRs*, and *BgobIRs*, respectively.

### 3.3. Identification and Phylogenetic Analysis of Chemosensory Genes in B. gobica

#### 3.3.1. OBPs

A total of 15 OBP genes (*BgobOBPs*) were identified *B. gobica*, which were all identified from the antennae Illumina transcriptome. These encoded proteins ranged in length from 70 to 243 amino acids, with molecular weights between 7.8 and 25.9 kDa and isoelectric points between 4.18 and 9.44. All 15 sequences contained complete ORFs, and 11 of them possessed N-terminal signal peptides ranging from 17 to 25 amino acids in length (Supplementary Table S7).

All proteins were functionally annotated via conserved domain searches within the insect odorant-binding protein superfamily, including its key subfamilies: pheromone-binding protein (PBP) and general odorant-binding protein (GOBP).The homology analysis revealed that five BgobOBPs shared over 75% sequence identity with OBPs from *D. citri*.

Based on the number and location of conserved cysteines, the 15 BgobOBPs were classified into three subfamilies: classic, minus-C, and plus-C. A sequence analysis revealed that the subfamily with the largest number of BgobOBPs (12) was the classic subfamily, which displayed the typical six conserved cysteines. BgobOBP3 contained only four conserved cysteines and was classified as a Minus-C OBP, and BgobOBP12 and BgobOBP13 were clustered with the Plus-C subfamily (Supplementary Figure S3).

**Figure 1:**
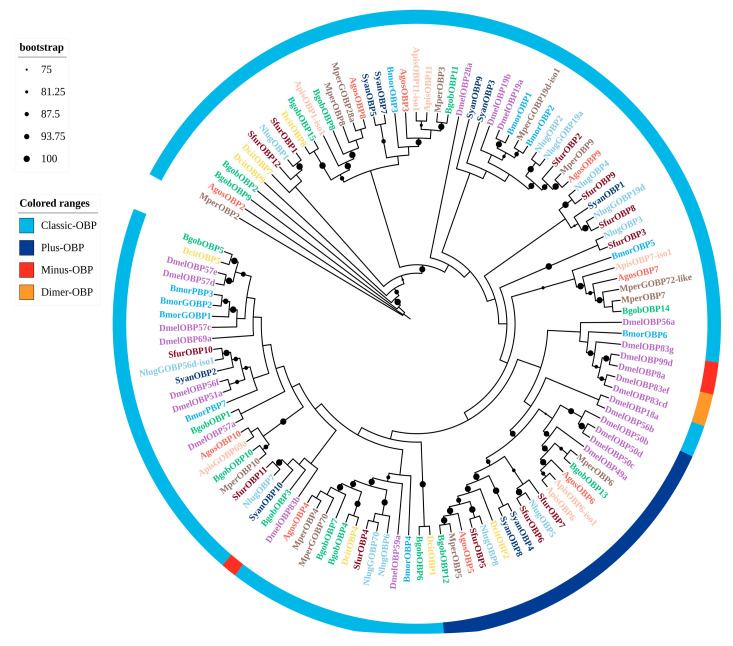
Maximum likelihood phylogenetic tree of candidate OBPs from *B. gobica*. The tree was constructed using predicted OBP protein sequences from *B. gobica*, as well as homologs from seven hemipterans and the model organisms *Drosophila melanogaster* and *Bombyx mori* (*Bactericetra gobica*: Bgob; *Diaphorina citri*: Dcit; *Aphis gossypii*: Agos; *Myzus persicae*: Mper; *Sogatella furcifera*: Sfur; *Bombyx mori*: Bmor; *Drosophila melanogaster*: Dmel; *Nilaparvata lugens*: Nlug; *Subpsaltria yangi*: Syan; and *Acyrthosiphon pisum*: Apis). OBPs from different species are highlighted in different colors and labeled with species abbreviations. Bars delimit clusters of *B. gobica* OBPs and their orthologs. Branches with circles are highly supported branches with bootstrap values > 75%. Distinct color-coding in ranges is used to denote specific OBP subtypes: light blue (Classic-OBP), dark blue (Plus-OBP), red (Minus-OBP), yellow (Dimer-OBP).

A phylogenetic tree was constructed using BgobOBP candidates along with OBPs from seven hemipteran species and model insects, including *Drosophila melanogaster* and *Bombyx mori* (Figure 1). The BgobOBPs were found to comprise distinct subclades, indicating relatively low intra-species sequence similarity and low species specificity. BgobOBP4 clustered with DcitOBP4*,* BgobOBP6 clustered with DcitOBP1, and BgobOBP15 clustered with DcitOBP8. This high sequence similarity and specific clustering likely reflect conserved orthologous relationships and potentially conserved functions for these OBPs in chemoreception within the Psyllidae family (Figure 1).

#### 3.3.2. CSPs

A total of 18 candidate *CSP* transcripts were identified in *B. gobica,* including 9 *CSPs* from the illumina transcriptome and 9 from the long-read sequencing Pacbio transcriptome. These 18 *BgobCSPs* encoded proteins ranging from 91 to 183 amino acids in length, all of which had complete ORFs. The predicted molecular weights ranged from 10.6 to 19.7 kDa, and the isoelectric points ranged from 4.94 to 9.83. Seventeen of the BgobCSPs possessed signal peptides, which ranged from 19 to 29 amino acids in length (Supplementary Table S7).

**Figure 2:**
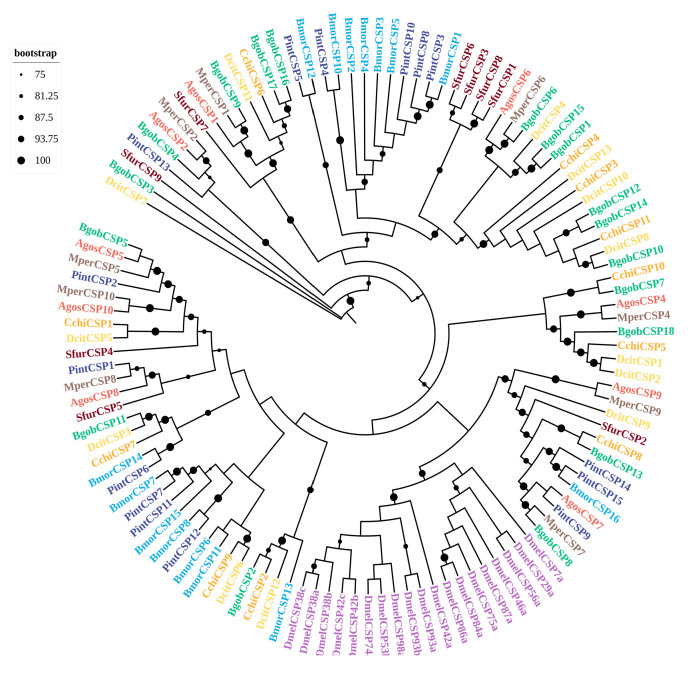
Maximum likelihood phylogenetic tree of candidate CSPs from *B. gobica*. The tree was constructed using predicted CSP protein sequences from *B. gobica* and homologs from five hemipterans and the model organisms *Drosophila melanogaster* and *Bombyx mori*, and *Plodia interpunctella*. (*Bactericetra gobica*: Bgob; *Diaphorina citri*: Dcit; *Aphis gossypii*: Agos; *Myzus persicae*: Mper; *Sogatella furcifera*: Sfur; *Plodia interpunctella*: Pint; *Cacopsylla chinensis*: Cchi; *Bombyx mori*: Bmor; and *Drosophila melanogaster*: Dmel). CSPs from different species are highlighted in different colors and labeled with species abbreviations. Bars delimit clusters of *B. gobica* CSPs and their orthologs. Branches with circles correspond to highly supported branches with bootstrap values > 75%.

A sequence analysis revealed that 16 BgobCSPs displayed the characteristic four-cysteine signature of the CSP family (C1-X6-C2-X18-C3-X2-C4) (Supplementary Figure S4). A homology analysis showed that five BgobCSPs shared more than 55% identity with *D. citri* CSPs; two were 100% identical to *Myzus persicae* CSPs. A phylogenetic analysis revealed that, except for the *D. melanogaster* CSPs that clustered together, CSPs from hemipteran species were scattered across different subfamilies. Several BgobCSPs showed close phylogenetic relationships with CSPs from *D. citri*, *Cacopsylla chinensis*, and *M. persicae*. For example, BgobCSP1 and BgobCSP15 clustered with DcitCSP4*,* BgobCSP10 clustered with DcitCSP8*,* and BgobCSP11 clustered with DcitCSP3. BgobCSP9 clustered with MperCSP1*,* and BgobCSP7 clustered with CchiCSP10 (Figure 2).

#### 3.3.3. SNMPs

Three SNMP genes were identified from the antennae illumine transcriptome of *B. gobica*. These BgobSNMPs encoded proteins of 40–117 amino acids in length, each with a complete ORF. The molecular weights ranged from 4.8 to 13.8 kDa, with isoelectric points between 6.13 and 10. Structural predictions indicated that each protein contained two transmembrane domains, one at the N-terminus and one at the C-terminus (Supplementary Table S7).

**Figure 3:**
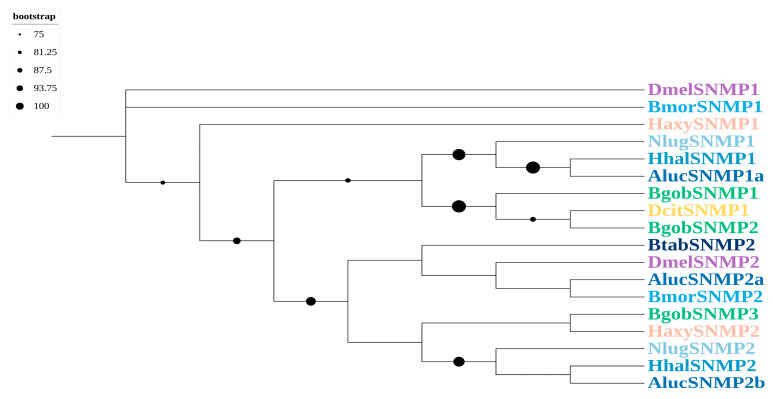
Maximum likelihood phylogenetic tree of candidate SNMPs from *B. gobica*. The tree was constructed using predicted SNMP protein sequences from *B. gobica* and homologs from six hemipterans and the model organisms *Drosophila melanogaster* and *Bombyx mori* (*Bactericetra gobica*: Bgob; *Diaphorina citri*: Dcit; *Harmonia axyridis*: Haxy; *Nilaparvata lugens*: Nlug; *Halyomorpha halys*: Hhal; *Apolygus lucorum*: Aluc; *Bemisia tabaci*: Btab; *Bombyx mori*: Bmor; *Drosophila melanogaster*: Dmel). SNMPs from different species are highlighted in different colors and labeled with species abbreviations. Bars delimit clusters of *B. gobica* SNMPs and their orthologs. Branches with circles correspond to highly supported branches with bootstrap values > 75%.

A phylogenetic analysis indicated that BgobSNMP1 and BgobSNMP2 were more closely related to SNMP1 in *D. citri*. The BgobSNMPs had homologs with DcitSNMP with identities of up to 80%, suggesting that they may belong to SNMP1-type proteins. In addition, BgobSNMP3 clustered with SNMP2 from other Hemiptera species, indicating that it is likely an SNMP2-type protein (Figure 3).

#### 3.3.4. ORs

A total of 26 odorant receptors (*BgobORs*) were identified in *B. gobica*, including 25 *BgobORs* identified from the antennae illumine transcriptome, and 1 *BgobOR* from the Pacbio transcriptome. Of these, 25 *BgobORs* were identified from antennal transcriptomes, and *BgobOR26* was detected from the body transcriptome. The protein lengths ranged from 94 to 486 amino acids. Twenty-three *BgobORs* had full-length ORFs, and *BgobOR13*, *BgobOR22*, and *BgobOR24* had partial ORFs.

The predicted molecular weights of these ORs ranged from 11.5 to 57.8 kDa, with isoelectric points between 6.39 and 10.32. The TMHMM Server v2.0 predicted that 24 of the ORs contained between 1 and 7 transmembrane domains, and BgobOR5 and BgobOR24 lacked predicted transmembrane structures (Supplementary Table S7).

**Figure 4:**
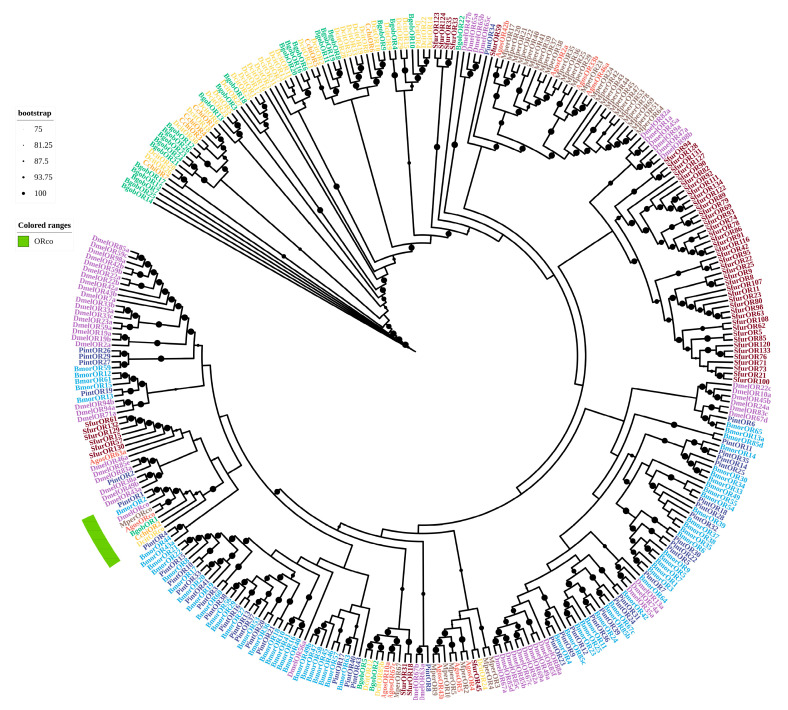
Maximum likelihood phylogenetic tree of candidate ORs from *B. gobica*. The tree was constructed using predicted OR protein sequences from *B. gobica* and homologs from five hemipterans and the model organisms *Drosophila melanogaster* and *Bombyx mori*, and *Plodia interpunctella*. (*Bactericetra gobica*: Bgob; *Diaphorina citri*: Dcit; *Aphis gossypii*: Agos; *Myzus persicae*: Mper; *Sogatella furcifera*: Sfur; *Plodia interpunctella*: Pint; *Cacopsylla chinensis*: Cchi; *Bombyx mori*: Bmor; *Drosophila melanogaster*: Dmel). ORs from different species are highlighted in different colors and labeled with species abbreviations. Bars delimit clusters of *B. gobica* ORs and their orthologs. Branches with circles correspond to highly supported branches with bootstrap values > 75%. Green color-coding in the range is used to denote Orco.

A phylogenetic tree was constructed using the JTT+F+G4 model to establish their evolutionary relationships and identify potential orthologs. The results showed that 14 BgobORs were homologous to ORs from *D. citri*, with identities ranging from 26.8 to 90.46%, and 11 BgobORs showed high similarity to ORs from *Drosophila melanogaster*, with identities ranging from 19.01 to 27.71%. A phylogenetic analysis showed that ORs were highly conserved within species. BgobOR1 clustered with DcitOrco*,* MperOrco*,* AgosOrco*,* and DmelOrco in a highly conserved clade, supporting its identification as the *Orco* gene in *B. gobica*. All BgobORs clustered with ORs from *D. citri*, suggesting that they are closely related (Figure 4).

#### 3.3.5. GRs

A total of eight *GR* transcripts were identified from *B. gobica*, including seven *GRs* from the antennal Illumina transcriptome and *BgobGR8* from the Pacbio transcriptome. These eight BgobGRs ranged in size from 46 to 331 amino acids, and all possessed complete ORFs. Their predicted molecular weights ranged from 5.4 to 38.1 kDa, with isoelectric points between 5.25 and 9.61. The TMHMM Server v.2.0 predicted that all eight *BgobGRs* contained transmembrane domains, with the number of transmembrane domains ranging from 1 to 7 (Supplementary Table S7).

A maximum likelihood phylogenetic tree was constructed based on GR sequences previously identified in psyllids and model insects, revealing several distinct GR subfamilies. The tree was generated using the mtInv+F+G4 model. A phylogenetic analysis showed that all BgobGRs were closely clustered with GRs from *D. citri* and *Planococcus citri*.

**Figure 5:**
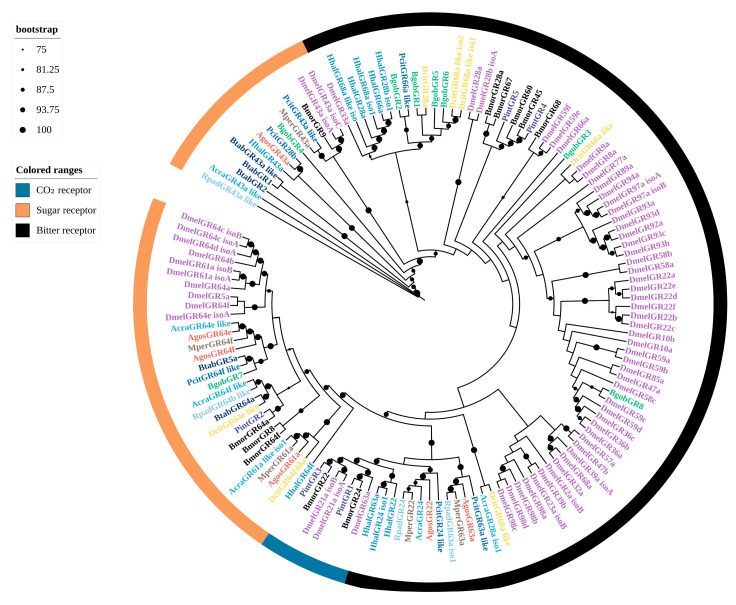
Maximum likelihood phylogenetic tree of candidate GRs from *B. gobica*. The tree was constructed using predicted GR protein sequences from *B. gobica* and homologs from eight hemipterans and the model organisms *Drosophila melanogaster* and *Bombyx mori*, and *Plodia interpunctella*. (*Bactericetra gobica*: Bgob; *Diaphorina citri*: Dcit; *Aphis gossypii*: Agos; *Myzus persicae*: Mper; *Plodia interpunctella*: Pint; *Aphis craccivora*: Acra; *Bemisia tabaci*: Btab; *Halyomorpha halys*: Hhal; *Planococcus citri*: Pcit; *Rhopalosiphum padi*: Rpad; *Bombyx mori*: Bmor; *Drosophila melanogaster*: Dmel). GRs from different species are highlighted in different colors and labeled with species abbreviations. Bars delimit clusters of *B. gobica* GRs and their orthologs. Branches with circles correspond to highly supported branches with bootstrap values > 75%. Distinct color-coding in ranges is used to denote specific GR subtypes: dark blue (CO_2_ receptor), yellow (Sugar receptor), black (Bitter receptor).

No BgobGR candidates were found within the clade of CO_2_ receptor orthologs (e.g., *DmelGR* CO_2_ receptor clade, shown as the blue clade in Figure 5). Based on known *Drosophila melanogaster* GRs, the BgobGRs could be divided into two major functional groups: sugar receptors and bitter receptors. Two candidate BgobGRs were identified in the sugar receptor clade, and six candidates were clustered in the bitter receptor clade (Figure 5).

#### 3.3.6. IRs

We identified 32 transcripts in *B. gobica* belonging to the *IR* family. Of these, 29 were identified from antennal Illumina transcriptomes and 3 from body Pacbio transcriptomes. These *BgobIRs* encoded proteins ranging from 24 to 956 amino acids in length, with 22 containing complete ORFs. Ten IRs, including *BgobIR10*, *BgobIR13*, *BgobIR21–24*, and *BgobIR26–29*, were partial sequences.

The predicted molecular weights of the IR proteins ranged from 3.4 to 106.9 kDa, with isoelectric points between 4.99 and 9.71. The TMHMM Server v2.0 was used to predict transmembrane domains in 25 IRs, and one to five domains were predicted per protein. A homology analysis revealed that 17 BgobIRs were homologous to IRs from *D. citri*, with 14 showing more than 75% sequence similarity. Nine BgobIRs were homologous to IRs from *M. persicae*, all of which had over 99% identity (Supplementary Table S7).

**Figure 6:**
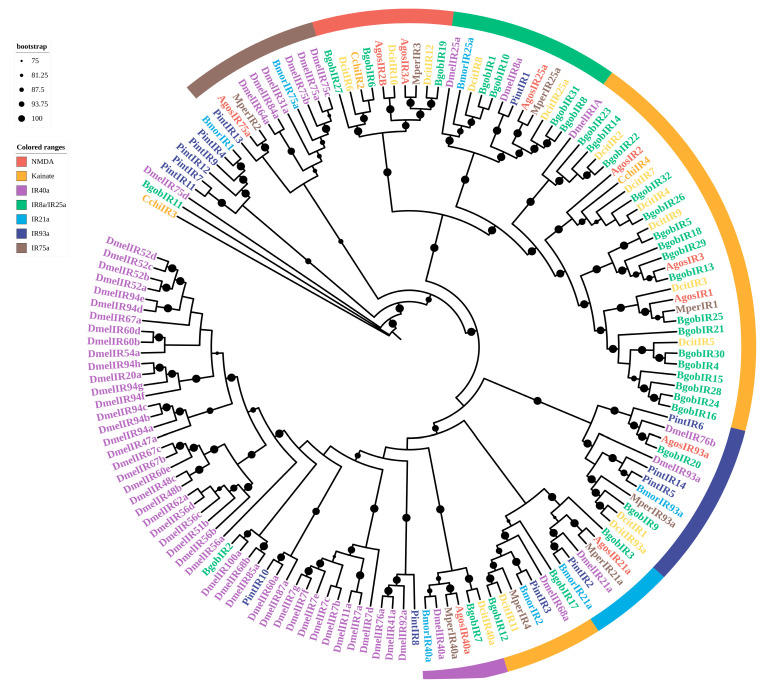
Maximum likelihood phylogenetic tree of candidate IRs from *B. gobica*. The tree was constructed using predicted IR protein sequences from *B. gobica* and homologs from four hemipterans and the model organisms *Drosophila melanogaster* and *Bombyx mori*, and *Plodia interpunctella*. (*Bactericetra gobica*: Bgob; *Diaphorina citri*: Dcit; *Aphis gossypii*: Agos; *Myzus persicae*: Mper; *Plodia interpunctella*: Pint; *Cacopsylla chinensis*: Cchi; *Bombyx mori*: Bmor; and *Drosophila melanogaster*: Dmel). IRs from different species are highlighted in different colors and labeled with species abbreviations. Bars delimit clusters of *B. gobica* IRs and their orthologs. Branches with circles correspond to highly supported branches with bootstrap values > 75%. Distinct color-coding in ranges is applied to denote specific receptor subtypes: red (NMDA), yellow (Kainate), purple (IR40a), green (IR8a/IR25a), light blue (IR21a), dark blue (IR93a), and brown (IR75a).

We reconstructed a maximum likelihood phylogenetic tree using the LG+F+R8 model. The phylogenetic analysis identified several IR subgroups, including IR8a/25a, IR21a, IR40a, IR75a, IR93a, Kainate receptors, and NMDA receptors (i.e., N-methyl-D-aspartate receptor) (Figure 6). NMDA and Kainate receptors from different species each formed distinct clades. Based on their clustering patterns, BgobIR6*,* BgobIR19*,* and BgobIR27 were predicted to be NMDA receptors, and 19 BgobIRs were clustered with Kainate-type receptors. BgobIR1, BgobIR8, BgobIR10, and BgobIR31, along with IR25a and IR8a from other species, were clustered together, suggesting that these BgobIRs may be co-receptors.

### 3.4. Expression Abundance of Chemosensory Genes in B. gobica

#### 3.4.1. Expression Profiles of BgobOBPs, BgobCSPs, and BgobSNMPs

**Figure 7:**
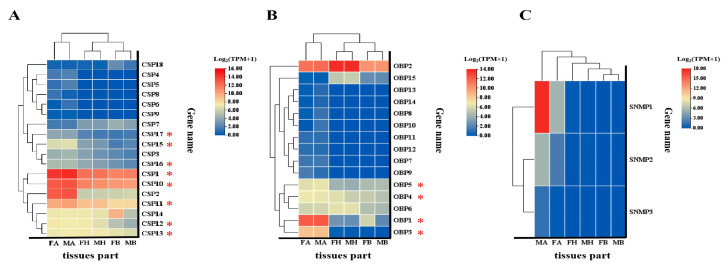
Abundance (TPM: Transcripts Per Millionvalues) of *B. gobica* OBPs, CSPs, and SNMPs in the transcriptomes of females and males. The x-axis shows the abbreviations of different tissue types. The y-axis represents the TPM values (log-transformed) across these tissues. (**A**) TPM values of CSPs in different tissues; (**B**) TPM values of OBPs in different tissues; (**C**) TPM values of SNMPs in different tissues. FA: female antennae; MA: male antennae; FH: female head; MH: male head; FB: female body; MB: male body. Red asterisks *; genes were significantly more abundant in female and male antennae than in other body tissues of *B. gobica* according to *p*-value < 0.05. Data are presented as mean values, with *n*= 3 for each tissue of both female and male *B. gobica*.

To explore the expression patterns of chemosensory genes in *B. gobica*, transcript abundance was analyzed using TPM (Transcripts Per Million) values. Among the BgobOBP genes, *BgobOBP1*/2 showed the highest expression level, which was significantly higher than that of the other BgobOBP genes (Kruskal–Wallis test, H(14) = 72.196, *p* < 0.001). Four BgobOBP genes (*BgobOBP1*, *3*, *4*, and *5*) exhibited significantly higher expression levels in the antennae (one-way ANOVA, F = 1439.771, *p* < 0.001; Kruskal–Wallis test, H(5) = 14.38, *p* = 0.013; Kruskal–Wallis test, H(5) = 14.614, *p* = 0.012; Kruskal–Wallis test, H(5) = 16.111, *p* = 0.007). *BgobOBP6* and *BgobOBP15* were highly expressed in the head (one-way ANOVA, F = 18.482, *p* < 0.001; Kruskal–Wallis test, H(5) = 15.316, *p* = 0.009). Among the 18 BgobCSP genes, *BgobCSP1* showed the highest expression levels (Kruskal–Wallis test, (H(17) = 12.76, *p* = 0.005)), with mean TPM values of 23,190. *BgobCSP1*, *10–13*, *15–17* also showed significantly higher expression in the antennae (one-way ANOVA, F = 136.854, *p* < 0.001; one-way ANOVA, F = 166.805, *p* < 0.001; one-way ANOVA, F = 47.373, *p* < 0.001; one-way ANOVA, F = 32.088, *p* < 0.001; one-way ANOVA, F = 15.955, *p* < 0.001; Kruskal–Wallis test, H(5) = 16.158, *p* < 0.006; one-way ANOVA, F = 10.839, *p* < 0.001; one-way ANOVA, F = 29.961, *p* < 0.001). Among the BgobSNMP genes, *BgobSNMP1* was the most highly expressed, and its expression was significantly higher than that of other BgobSNMPs in the antennae (Kruskal–Wallis test, H(2) = 14.76, *p* < 0.001). Although other genes (e.g., *BgobOBP7*, *BgobOBP8*, *BgobOBP10–13*, *BgobSNMP1–3*) also displayed some variation in expression among the antennae, head, and body tissues, these differences were not statistically significant (Figure 7, Supplementary Table S8).

#### 3.4.2. Expression Profiles of BgobORs, BgobGRs, and BgobIRs

**Figure 8:**
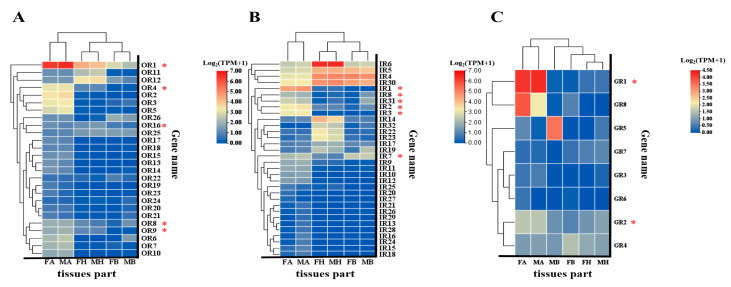
Abundance (TPM: Transcripts Per Millionvalues) of *B. gobica* ORs, IRs, and GRs in the transcriptomes of females and males. The x-axis shows the abbreviations of different tissue types. The y-axis represents the TPM values (log-transformed) across different tissues. (**A**) TPM values of ORs in different tissues; (**B**) TPM values of IRs in different tissues; (**C**) TPM values of GRs in different tissues. FA: female antennae; MA: male antennae; FH: female head; MH: male head; FB: female body; MB: male body. Red asterisks *; genes were significantly more abundant in female and male antennae than in other body tissues of *B. gobica* according to *p*-value < 0.05. Data are presented as mean values, with *n* = 3 for each tissue of both female and male *B. gobica*.

More than half of the BgobORs were specifically or predominantly expressed in antennae. Although a few BgobORs, BgobGRs, and BgobIRs had TPM values greater than 5 in the antennae, the majority were expressed at relatively low levels. Among the 26 BgobORs, *BgobOR1* (the *Orco* gene) was the most highly expressed in antennae (average TPM = 94), and all others had TPM values below 5. Five BgobORs were significantly expressed in both male and female antennae, including *BgobOR1*, *4*, *8*, *9*, *16* (Kruskal–Wallis test, H(5) = 15.503, *p* = 0.008; one-way ANOVA, F = 426.096, *p* < 0.001; Kruskal–Wallis test, H(5) = 15.339, *p* = 0.009; Kruskal–Wallis test, H(5) = 15.661, *p* = 0.008; one-way ANOVA, F = 18.482, *p* < 0.001). Among the 32 IRs, those with high antennal expression included *BdgobIR1–3*, and 7–*8*, *31* (Kruskal–Wallis test, H(5) = 15.503, *p* = 0.008; Kruskal–Wallis test, H(5) = 14.380, *p* = 0.013; Kruskal–Wallis test, H(5) = 14.158, *p* = 0.015; one-way ANOVA, F = 9.971, *p* = 0.001; one-way ANOVA, F = 86.091, *p* < 0.001; Kruskal–Wallis test, H(5) = 15.924, *p* = 0.007). We also found that *BgobIR 17*, *30*, and *32* were highly expressed in the head (one-way ANOVA, F = 29.961, *p* < 0.001; one-way ANOVA, F = 19.478, *p* < 0.001; Kruskal–Wallis test, H(5) = 15.480, *p* = 0.008). Among the eight BgobGRs, the expression of *BgobGR1*, *2* was significantly higher in antennal tissues than in other tissues (Kruskal–Wallis test, H(7) = 27.48, *p* < 0.001). Though no significant difference was found between the two sexes, the TPM value of *BgobGR5* in body tissues in males was higher than in females (Kruskal–Wallis test, H(7) = 11.510, *p* < 0.042). Other BgobOR, BgobGR, and BgobIR genes showed no significant expression differences across the antennae, head, and body tissues (Figure 8,  Supplementary Table S8).

#### 3.4.3. Real-Time PCR Validation of the Expression of Chemosensory Genes

**Figure 9:**
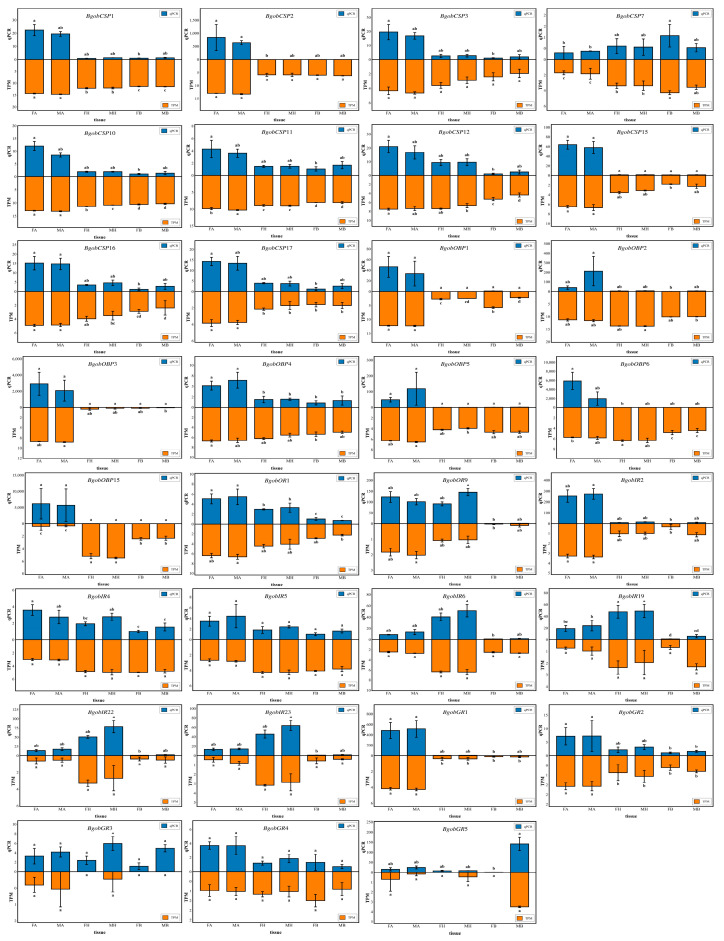
Verification of the expression levels of 31 chemosensory genes in different tissues of *B. gobica* using real-time PCR. FA: female antennae; MA: male antennae; FH: female head; MH: male head; FB: female body; MB: male body. Data that conformed to a normal distribution with homogeneous variance were analyzed using a one-way ANOVA followed by Duncan’s multiple range test, while the remaining data were analyzed using a Kruskal–Wallis test in SPSS 22.0 software. Different lowercase letters (a, b) indicate statistically significant differences among tissues (*p* < 0.05), where groups marked with distinct letters (e.g., a vs. b) differ significantly. Groups sharing the same letter or overlapping letters (e.g., a vs. ab) show no significant difference. Data are shown as mean ± SEM.

To validate the transcriptome-based expression profiles, a qPCR analysis of 31 chemosensory genes with high abundance, antennae-biased, and sex-biased expression in *B. gobica* was performed, including *7 BgobOBPs, 10 BgobCSPs, 2 BgobORs, 5 BgobGRs,* and *7 BgobIRs.* The results of the qPCR analyses were consistent with the results of the transcriptome analysis (Figure 9). Genes highly expressed in the antennae included *BgobOBP2*, *4*, *6*; *BgobCSP1–3*, *10–12*, *16–17*; *BgobOR1*, *9*; *BgobIR2*, 4 (Kruskal–Wallis test, H(5) = 14.895, *p* = 0.011; one-way ANOVA, F = 13.006, *p* < 0.001; Kruskal–Wallis test, H(5) = 14.988, *p* = 0.010; Kruskal–Wallis test, H(5) = 14.287, *p* = 0.014; Kruskal–Wallis test, H(5) = 16.111, *p* = 0.007; Kruskal–Wallis test, H(5) = 13.678, *p* = 0.018; Kruskal–Wallis test, H(5) = 14.427, *p* = 0.013; Kruskal–Wallis test, H(5) = 13.491, *p* = 0.019; Kruskal–Wallis test, H(5) = 15.129, *p* = 0.010; Kruskal–Wallis test, H(5) = 14.520, *p* = 0.013; Kruskal–Wallis test, H(5) = 14.848, *p* = 0.011; one-way ANOVA, F = 15.989, *p* < 0.001; Kruskal–Wallis test, H(5) = 15.409, *p* = 0.009; Kruskal–Wallis test, H(5) = 15.222, *p* = 0.009; one-way ANOVA, F = 10.343, *p* = 0.001); and *BgobGR1*, *2* (Kruskal–Wallis test, H(5) = 15.971, *p* = 0.007; Kruskal–Wallis test, H(5) = 14.661, *p* = 0.012). Only genes showing a significant difference in the transcriptome data (|log_2_(FoldChange)| ≥ 5 and *p* ≤ 0.05) and a significant difference in the qPCR results (*p* ≤ 0.05) were classified as sex-differentially expressed genes. *BgobGR5* was highly expressed in body tissues, and its expression was significantly higher in males than females (Kruskal–Wallis test, H(5) = 15.363, *p* = 0.009). The expression level in the male body was 140-fold higher than in the female body.

## 4. Discussion

The psyllid *B. gobica* is an economically significant pest that infests goji berry plants, yet research on chemosensory genes in *B. gobica* remains limited. To obtain sequence data for chemosensory genes in *B. gobica*, we sequenced the antennae and body tissues using Illumina sequencing and the long-read sequencing transcriptomes using Pacbio sequencing. From antennal and body transcriptomes, we identified a total of 102 chemosensory-related genes, including 15 OBPs, 18 CSPs, 26 ORs, 8 GRs, 32 IRs, and 3 SNMPs, which indicates that the antennae and bodies of *B. gobica* have a diverse array of chemosensory genes. Because of the small RNA quantity per antenna, pooling 400 pairs of antennae per replicate was necessary; however, its potential impact on biological variation should be considered.

A total of 9 OBPs, 12 CSPs, 46 ORs, 35 IRs, 20 GRs, and 4 SNMPs were identified in the Asian citrus psyllid *D. citri* [29]. In the pear psyllid *Cacopsylla chinensis*, 12 OBPs and 11 CSPs have been identified. These findings suggest that the number of CSPs and OBPs is similar among psyllid species such as *B. gobica*, *D. citri*, and *C. chinensis*. However, *B. gobica* possesses fewer ORs than *D. citri*, which may be related to its monophagous feeding habits.

A phylogenetic analysis of the 15 identified BgobOBPs showed that, similar to *D. citri*, most belonged to the classic OBP type [30]. *BgobOBP12* and *BgobOBP13* belonged to the Plus-C subfamily. These two proteins are both characterized by additional cysteine residues at the N-terminus, two to three additional cysteines downstream of C6, and a conserved proline following the seventh cysteine. *BgobOBP1* and *BgobOBP2* were primarily expressed in antennae, and TPM values for these genes were higher than those for most other *BgobOBP* genes. In addition to the OBPs, *BgobCSP1*, *10–13*, *15–17* also showed antennae-biased expression. These antennae-enriched OBPs and CSPs may play key roles in detecting environmental chemical cues. *BgobOBP1* and *BgobCSP10* were highly expressed in female antennae, suggesting that they are potentially involved in the perception of host plant volatiles (camphor, decahydro-2-naphthalenone, and acetic acid). BgobOBPs and BgobCSPs were clustered with other psyllids such as *D. citri* and *C. chinensis*. BgobOBPs and BgobCSPs also clustered with aphid species, including *M. persicae* and *A. gossypii*, indicating that they are closely related to these species and potentially have similar chemosensory systems capable of detecting similar odorants.

DcitOBP7 binds strongly to host plant volatiles. RNA interference of *DcitOBP7* reduces antennal electroantennogram responses and impairs olfactory attraction to host plant odors [31]. Recent studies have shown that OBPs and CSPs may also perform non-olfactory physiological functions, including enhancing the resistance of insects to various insecticides such as chlorpyrifos, spinetoram, lambda-cyhalothrin, and spirotetramat [32,33]. For example, in *D. citri*, the expression of *DcitOBP9* is significantly induced upon exposure to the neonicotinoid imidacloprid [34]. The silencing of *DcitpCSP8* enhances resistance to thiamethoxam [35]. Additionally, CSPs may function as effectors in plant–insect interactions and potentially activate plant immune responses [36,37]. Thus, OBPs and CSPs play crucial roles in signal perception and behavioral regulation in hemipteran pests, making them valuable targets for pest control strategies. However, functional assays and expression profiling under chemical exposure conditions were not investigated in this study. Future studies investigating these aspects would be valuable to provide a more comprehensive understanding.

SNMPs belong to the large CD36 protein family and perform various functions. A detailed phylogenetic analysis is often required to distinguish between SNMP subtypes [38,39]. We identified three SNMPs in *B. gobica*. SNMP1 and SNMP2 in *D. citri* and other insects are often expressed in the antennae or terminal abdomen [30,40,41]. SNMP1 proteins are thought to function as “tunnel” proteins in pheromone detection, and they mediate the transfer of odorant molecules from OBPs to ORs; SNMP2 might be involved in pheromone degradation [42,43]. In our study, *BgobSNMP1* was most highly expressed in the antennae, suggesting that it might play a specific role in pheromone detection.

Insect ORs function as heterocomplexes composed of ligand-specific ORs and the co-receptor *Orco*. In this study, we identified *BgobOR1* as the *Orco* gene in *B. gobica*. Previous studies have shown that the *Orco* of the peach aphid consists of 498 amino acids [44]; the *Orco* of the Asian citrus psyllid consists of 461 amino acids [30]. These *Orcos* all possess seven transmembrane domains. The *BgobOR1* identified here consists of 481 amino acids, with characteristics similar to those of Drosophila, the peach aphid, and the Asian citrus psyllid. Insects rely on plant volatiles to locate suitable hosts for feeding and oviposition. ORs play an important role in detecting plant volatile compounds for the host. We found that five *BgobORs* were significantly more abundant in the antennae of both male and female insects, suggesting that they play an important role in the olfactory perception of *B. gobica*. ORs have the potential to aid the development of green pest control strategies, including biosensors for pest monitoring and repellent screening via reverse chemical ecology [45]. Attractants and repellents for *B. gobica* could be screened in future studies of these five highly expressed BgobORs.

Chemoreception includes both olfaction and gustation [45]. Currently, few studies on insect gustatory receptors have been conducted, and most receptor studies have focused on Lepidoptera and the model organism *Drosophila*. Among the eight GRs identified in this study, GR1 was highly expressed, indicating that it has an important chemosensory function. The number of gustatory receptor genes was lower in the goji berry psyllid *B. gobica* than in the Asian citrus psyllid *D. citri* (20), indicating that *B. gobica* has a relatively smaller gustatory receptor gene family. *D. citri* is a monophagous herbivore, while *B. gobica* is an oligophagous herbivore. This is consistent with the results of previous studies indicating that insects with broader diets tend to have greater numbers of GRs [46,47]. Interestingly, although *B. gobica* has fewer GRs than *D. citri*, the proportion of BgobGRs clustered with bitter taste receptors from other species remains high (six out of eight). It has been reported that the monophagy of *Bombyx mori* is determined by the combined function of multiple bitter GRs involved in detecting specific secondary metabolites in mulberry leaves [48]. Solanaceae plants contain various glycoside alkaloids and other secondary metabolites. These six bitter taste receptors in *B. gobica* may play important roles in mediating the specific adaptation of *B. gobica* to feeding on wolfberry plants (Solanaceae). However, this speculative hypothesis still needs further functional data support. Previous studies have shown that *IR8a* and *IR25a* act as co-receptors and are co-expressed with other IRs to form ligand-gated ion channels [49,50]. A response to phenylacetaldehyde requires the co-expression of *DemlIR84a* and *DemlIR8a* in *Drosophila* [51,52]. In this study, BgobIR1, BgobIR8, BgobIR10, BgobIR31, and the IR8a/IR25a of other insects were clustered within the same evolutionary clade, suggesting that BgobIR1, BgobIR8, BgobIR10, and BgobIR31 may function as IR co-receptors in *B. gobica*. Gene editing and behavioral analyses in model organisms such as *Drosophila* and Lepidoptera have demonstrated that IRs play a role in temperature sensing, the detection of certain odors, and gustation of various compounds, including acids, amines, aldehydes, and salts. A phylogenetic analysis of BgobIRs showed that BgobIR3 clusters with IR21a from other insects. In *Drosophila*, *DemlIR21a*, *DemlI93a*, and *DemlIR25a* influence the larval avoidance of high and low temperatures [53]. Thus, BgobIR3 may play a role in sensing temperature changes to guide behavioral responses. IR93a is thought to be involved in both humidity and temperature sensing [54]. BgobIR9 and BgobIR20 were clustered with IR93a from other insects, suggesting that they may be related to temperature and humidity perception. It should be noted that the open reading frames (ORFs) for some receptor genes (e.g., *BgobOR13, BgobOR22, BgobOR24, BgobIR10, BgobIR21–24*) are incomplete. Consequently, obtaining more comprehensive genome data is necessary to resolve these sequences.

Our transcriptome analysis of male and female *B. gobica* revealed *BgobGR5* was highly expressed in the male body. In *Drosophila*, GRs highly expressed in the male-specific gustatory bristles on body tissues were found to be involved in pheromone recognition and male courtship, which affect sperm viability or post-mating reproductive competition [55,56,57]. This male highly expressed GR found in our study may be involved in male mating behavior or pheromone detection in *B. gobica*.

## 5. Conclusions

We employed transcriptome sequencing combined with a bioinformatics analysis to comprehensively identify candidate chemosensory genes in *B. gobica*, an economically significant pest of goji berry. A total of 102 chemosensory-related genes were identified, including 15 OBPs, 18 CSPs, 26 ORs, 8 GRs, 32 IRs, and 3 SNMPs. The expression profiles of these genes were analyzed by comparing TPM values across different body tissues. Quantitative real-time PCR validation of 31 selected genes confirmed their differential expression patterns in various tissues of *B. gobica*. We also identified gene *BgobGR5* with differential expression patterns between females and males. A phylogenetic analysis revealed orthologous chemosensory genes in the Asian citrus psyllid *D. citri*. A deeper understanding of the chemosensory system in *B. gobica* will aid the development of environmentally friendly pest monitoring and control strategies targeting olfactory and gustatory pathways. The transcriptome dataset generated in this study, which includes numerous full-length ORFs, provides a valuable resource for future research on chemosensation, detoxification, and related functional studies in *B. gobica* and other psyllid species.

## Data Availability

The data presented in this study are available on request from the corresponding author.

## Supplementary Materials

The following supporting information can be downloaded at: https://www.mdpi.com/article/10.3390/biology14081105/s1, Figure S1: Statistical map of Whole-organism gene annotation results; Figure S2: NR database alignment statistics chart for antennal genes; Figure S3: Amino acid multiple sequence comparison of OBPs of *Bactericera gobica*; Figure S4: Amino acid multiple sequence comparison of CSPs of *Bactericera gobica*; Figure S5: Differential expression of *actin* and *gapdh* in different tissue; Table S1: Quality Table for the Transcriptome Unigenes of *Bactericera gobica*; Table S2: Table of Frequency Distribution of Antenna Splicing Lengths; Table S3: List of Distribution of Antenna Splicing Lengths; Table S4: Whole-organism Gene Reads Statistics Results; Table S5: Whole-organism Gene Correction and Redundancy Removal Statistics Results; Table S6: Antennae Gene Annotation Statistics Table; Table S7: Genetic identification; Table S8: TPM; Table S9: amplification efficiency; Data File S10: Gene Sequences.
