# Supplementary material for: Transcriptome Screening and Identification of Chemosensory Genes in the Goji Berry Psyllid, Bactericera gobica (Hemiptera: Psyllidae)
Source: Biology (Basel). 2025 Aug 21;14(8):1105. doi: 10.3390/biology14081105 (PMC12383947; doi:10.3390/biology14081105)
Supplement: Supplementary file 1 [file biology-14-01105-s001.zip › biology-3758692-supplementary/Supplementary Files/Supplementary materials S10-Gene Sequences.pdf]

>CSP1
ATGTACAAAGTTTTAGCTTTCGCCGTCTGTTGTGCAGTGATCGTCAGCTG
TCTGGCGAAACCCCAGGACAAGGAGAAGAAGTACACCACCAAGTATGACA
ACATTGATCTGGAAGAGATCCTGAACAATGAGAGACTCCTCAAGAACTAC
TACAACTGTTTGATGGATGAGAGCCCATGCACACCTGATGGTGGTGAACT
CAAGAAACTCCTCCCCGACGCCCTTGAGAACGACTGCAAGTCCTGCTCCA
AGAAACAGAGGGAAGGTGCCGAGATCATGATCAAGCATCTGATCGAGAAC
AAGAAAGACATGTGGGAGAACCTCGAGAAGAAGTACGACAGCAACAAGGT
CTACAGAAAGAAGTACGCCAAGGAGGCCGAACAACGTGGCATCAAGGGCA
TCACCGCCGAATAG

>CSP2
ATGGATGTCAGCAGAGTTGCCCTCTTCGTTTTCATCTGCTGCGCAGCTCT
GACTGGTGCAACCCACGGGTATGAATTCGACGGCGGGGATGAGAGTGTGG
ACTGCGACCAATTCATGAGCAATGACCGTCTAGTCAATGGTGTGGTGGAG
TGCTTGATGTCGGACGATCCAGAGTGTGGCAGTGCGCTGTTCACCCAGGT
CAAACATTACGCTCCAGAGATCCTGGAGACCACATGTGCCAAATGCACCG
ACAAACAAAAGGAGAAGTTCAAGGAGTGCACCAACAAATTCATCAAGATC
CGTCCTGCCGACTATGAGGCAATCATCAAGAAGTACGACCCTGAAAACAA
ATATAGATCTGCGTTGGAAGCTTTCCTCGCTAGTTAG

>CSP3
ATGGCCACATCCGCACTTCTACCTACGGTTCTCATAGCGACAACATGCGT
TGCCATAGCAACCGGCGCCCCAGTTCCAGCCGACACGGGGCTCGCGAGCA
AATACGAAAGCTTCAACGTGGATAGCGTCATCGGCAATGATCGGATCCTA
ACGAACTACATCAAGTGTCTGATGGACCAGGGATCTTGTACGAACGAGGG
ACGAGATTTGAAAAAGACTATTCCAGACGCTCTGGCGGGAGGCTGTTCTC
AATGCTCTGAGAAGCAGAGACAGACCACTGAGAGAGTCATCAAGCATCTG
AAAGACAACCGGAAAAGCGATTGGGACCGACTAGTCAAGAAGTATGACCC
CAAGGGCATCTACGAAAAACAGTACCTTCAATTGTTGAGCCCACAGAAGT
CAGGTGCCACAGAGCAAGCCAAAGACGCAAAAGCTACATCGGAAAAGTCA
GCGGGCACCAAAGTCAGCACAGCAGCAGAAGTCCAGGCAAAAGAGAAGTC
GAAGCCTGCCGTGAAGAAACAAGCTCCAACACTGACAGCAGCTAAAATTT
GA
>CSP4
ATGTTGAAATGTTTATTAATTTGTTTTGAACAGAATTTTATATTAATTAT
ATTGTGTTGTGTGATTAAATTTACAGAAGTTTTACCAGACGCATTAAAAA
CTGACTGCAGCAAATGTACAGAAGTACAAAAAGACAGATCAGAAAAAGTG
ATTAAGTTTTTAATCAAGAACCGTTCTACTGACTTTGATCGTTTGACCGC
CAAATACGACCCATCGGGCGAATACAAGAAGAAGATCGAAAAATTCGACT
CTGAAAAGGCTGCAGCTGCTAAACATTAA
>CSP5
ATGAACTGCAAGGTCTTGATCGCTCTGTGCTGCGTGGCCGTGTACGCCGC

GCACGCCAGTCCCGCCGGTGCGGCCACAGCCGCCGCCGCATCCGCCGACG
AAGAGATCAAGGATTTCCCGGCCTACATGAAGCGGTTCGATAAGCTCAAC
GTGGAACAGGTTCTGAACAACGACCGCGTCCTGGCCAGCCATCTCAAGTG
TTTCCTCAACGAGGGCCCGTGCGTCCAGCAGTCCAGGGACTTGAAGAGAG
TCATCCCGGTGATCGCCAACAACGGCTGCAACGGATGCACCGAAAGACAG
ATGACCACCATCAAGAAGTCGCTGAACTTCTTGAGGACGAAGAAGCCAGT
CGAATGGGCGAGACTCGTCAAGATCTACGACCCGTCCGGCACCAAGTTGA
ACAAATTCCTCGACGCGTAA
>CSP6
ATGAACACACTTCTCCTAGCAGTTGCTCTTTGCATCGCCATCACGATGAC
TGTGGTCCAGACAGCACCTGCTAAATATACTACTAAGTACGACAACGTAA
ATATCGATGACATTCTGAACAACGACCGCTTGGTCGCCAGCTACTTTAAG
TGTCTGATGGAAACCGGAAAATGTACACCAGAAGGCGAAGAAATTAAACG
GTGGTTACCAGAAGCAATAGAGAATAAATGTGAAAACTGTTCGGAAAAAC
AAAAAATTGGTTCCGAAAAAATTATTAAGTTTCTAATTGAAAAGAAAAAT
GATATGTGGAAACAACTTGAACAAAAATATGACCCCCAAGGACTCTACAA
ACAACGTTATTCGGAAGAGGCAAAGAAATTGAACCTTGATGTTTAA
>CSP7
ATGAGTTCCTCAATGGCAGCCCTTGTGCTGGCAAGTTGCTTGACTTTCGT
GTTCAGCACCACCGTTCCGCTGACGCAAGTTGACAAAACGTCTGCGGCGT
CTCCAAACGACAAACAAGCCGGGTCGTTTCTGGATCGTTTGCCCATTCTG
GAGCTGCTGAAGGACCCTAAAGTGGTGGATAGATACACGGGCTGCTTCCT
GGACAAAGGCCCTTGCACGCCGGACGCAGCCGAAGTCAAAGGTATCTTGA
GTGATGCACTCACCAAAGGCTGCGCCAAGTGCACCCCCCTACAGAAGCAA
GTCAGCGAGAGGCTGATGCTGACTTACAACGAGAAGCAGCCTGAAGTGTT
CAAGCAGGTGACTGCCAAGTACGACGACAAGGGAGAGTTTAGCAAGAAAT
ATTTGGCTAGCTTGAAGAAGAGTCTGAAAAAGGAGTAG
>CSP8
ATGGATCGATCGTCGTCAAGTGTCACCATGAAAGTATTCGTAATCGCCGT
GTGCGTGTGCGCCGCACTCGCCCGTCCGGAAGACTCGAAAGTGGAAAACA
AACCGGCTGCGGTCAAATCGGAGACCTTGGCCGCACCACTTCCGACGACC
ATAGTGAAACGGGCTACTCCTCAGGTCGTCTCAACCCAGCAAGGCGCATC
GCTTCCGAACGTCAGCGAGGACGTACTTGATAAGGCGCTCAGTGACAGGA
GGTTCGTGCTAAGGCAACTCAAGTGTGCTACGGGCGAAGGACCGTGCGAC
CCCATTGGTCGAAAAATTAAAGCTCATGCACCGCTAGTGTTGAGAGGAAT
GTGCGTCAAGTGCTCGCAGTCGGAAATCAAACAGATTCAACGTGTCATGT
CACATATACAGAAGAATTATCCCAAGGAGTACACTATGATGCTCAAACAG
TACCAGAGCGGATTCTAA
>CSP9
ATGAACAATGAACGAATTATTAAAATTCTTTTCAACTGTGTTATGAACCA
AGGACCGTGTACCAGAGAAGGTTTGGAGCTTAAAAGGATTGTTCCGGATG
CAATACAGACAGAATGTGCTAAATGTAATGAAAGACAAAGAAAACAAGCA
GGCAAAGTTTTGGCTCATTTACTACAGTACAAACCAGAATACTGGAACAT
GTTGGTGAAAAAATTTGATCCAAATAATGTATATTTGAAAAAGTATATGG

CAGACAATGATGATGACGAGAAGGTATCTCTTCAAAAACTCACCAACGAT
ACAACCAAATAA

>OBP1
ATGATTTGCCTTTTTTTTCAGGCATTGCCCGAAGCTCAAATTAAAGCCTT
CAAGAAGCAAGTGAAAAATCAATGTCTTGCCAAAACCAAAGTCGATCCCG
GTTTGATTGACACTTTACTTAATGGAGAGTTTCCCGACGATCATAAAACA
GAGTGCTTCGTTAAATGCATCATGGACAAGTCAATGGTGACAACCAAGGG
CCGCATTGATTGGAAGAAGATCCAGACGACAGCCAAGGCCATGTTACCTC
CCAAACTGGCCAAGAAAGTAGAGGTGGTTGCGGGCGAATGCAAGGATATC
CCTCTAGAAGAAGACCTTTGTAAGCATGCCATGGTAGTCACCAAATGTGT
ATATAATGCTGATCAGGAACTTTTTAAATTCATGAACCCGGAGTAG
>OBP2
ATGGCTGCCAAACTAATTGTACTTGCCGCCCTCTGTGTTTACGTGCAGGC
TCAAAGCAGTTCGACCACCACCCTCCCCAACGTCGCCCAGAAAGTCAACC
TGGTCGCCACCAAATGTAAAACCGAGCTGAGTTCCCCACCTGAGGCCGTA
GCCCTACTGGGCTCCAAGGCCCTTCCCGCCGACGAGAAACAGAGATGCTT
CATGGAGTGCGTCTACAAGAACCTCAACCTGATCAAGGACAACAAGTTCA
ACGTCGAGGGAAGCAAAGCCCTGGCCGCCCAGAGATTCAAGGGCGACGAG
TTGACCAAGGCCAACAAGCTGATCGACACGTGCGCAAAGGAAGCGGTGGT
CGCCCCCGGTAACACCGAAAAGTGCGCCCTGGGCAAGTCGGTCCGATCCT
GTTTCGCCAAGAACGGAGACAAGATCAACTTCTTCCCCAAAGCTTGA
>OBP3
ATGTGCACCCTGCAGCAAATTTCGGCGATGGACGATGAAGGAAATGTAGA
CGCAGACGTTTTCATCGGTACCATGCCCGAAGAGTACAAGTCCTATGCCT
TGGAAGTTGTGGATAAGTGCACGCACATAAACGGTGCAACTCCATGTGAA
AAGGCTTACAACTTGAACGTGTGTGCTCAAAACGTGGATCCAAATAAATA
CATGTTCATTTGA
>OBP4
ATGGAATCTCCTCAGTGGGTCGTCTTCTTGTGTGTGGTGTTTTTCAGTCA
GGTGCTCTGTCAAATTCAGCACAGGCCGCAGCAAATTCTACAATCTAAAA
CAGCGGAGCCCCAAAAATTTTCAAAATGTGAGCCTCCATCATCCGCGCCA
CAAAAGTTGGAAAAAAATAATCGGCCAGTGTCAAGATGAGATCAAGCAGGC
ACTCCTACAAGAGGCTCTAGAAGTAATCGACGACGTTAAACATCAGATCA
ACAACAACAACAACAATCATAATCAAAACAACAACAATAACAACCAAAAC
AACAACAAAGGATTCAACAATCCCCACACCAGGGTCAAGAGAGAAAACTT
TTCGGGCGAGGAGAGGAGAGTGGCTGGATGCTTGTTGCAATGCGTCTACA
GAAAAGTCAAAGCAGTTGACTCCACAAACTTTCCTTCGGCTGAAGGACTG
GTGCGCCTGTACTCAGAAGGAGTCCAGGATAGGAACTACTTCACCGCCAC
CTACCAATCTGTACAGTTCTGCATGAAGCTGGCCGAGCAAGTCAGAGCGG
CCAAACCCAACGGAATTCTGGACGGAGGTCAAACATGTGACTTGGCGTAT
GATATGTTCAACTGTGTGAGCGACCAGATTGAGAAGTTCTGTGAAGTTCT
TGTATAG
>OBP5

ATGAGACTGGCAGGTGTCGTCTCTCTGACGGCTGTCTTGTCTCTCTGTGT
AGCCATCGCTTCGGGCAGATACTTACAAGATAGTGCTGAAGAAAAAGCAG
ATTTTGTTACGATGTGCAACAGCAGTTGGCCTACAGATACTGATATCCTC
ACCTCCGTCATCTCAGAGAAAAAGTTCCTCAGTATTCACAATAAAAATTT
CAAGTGTTTTCTTCACTGCCTCTACATCCACTACGAATGGATGGACCAGA
CTGGCGGCTTCCTGCTTCACAACATGAAGGAGGAGCTGCTGAGGACAAGC
TTGGATGATGAAACAGCCGACGTCATCCTGTTCAAATGCACAGCTATAGA
CTCCAGCCATGCCTGTGATAGAGCCTACAGGTTCACCGATTGTTTCTGGA
GGGAGACTCAAATGTACGGTGATGTAGAGACGAATGAGATTGACAAGTAC
GTGCACGTGGATTGA
>OBP6
ATGAATCTTCTCTTGCAATTCTCCAGTGTCAGATTGTTGCTGTTTGTTTC
CTTCTGTTTATTAGTGAACAATATTCTTCGAATTACGGCTTACTCCAAAA
ATGATCTCGATGATCTCGGAAAAACGTGCAATGCCTCCAAAGCCGATTTA
GAAGTCACCAAACAATTCGAAGTACCCGATTCAGAATCTGGCAAGTGTTT
CGCCAAGTGTATCCTCAGCACTCTCAACTTGATGAATACCGCAGGCGAGA
TTGACAAGGAAGCCTCCATTCAAAGCATGATCAAGTATTGGCCGAATTTC
AGTGCAGAGCTTAATGGCCAAATTGCCAAACAGTGCTACGACCAGGTGAG
TCCCTTCCACAAGGAACTGACCGGCACTTGTGAGCTCGCATACAAGATGC
TCAAATGTTTGAACGTCGAGTCCAGGAAGCACGGATACTACGAAGACTTC
CTCAAACCTTAA
>OBP7
ATGCGTGGAAATTATTCTTTGACGGTTTTCCTTCTTTTTGTTATTGGATT
ACAAGATATTTATTGTCAAAAACAAGAACCATCAGGAAAATGTAGAGCTC
CCGATAAGGCGCCTTTAAATCTTGAAATAATAATTAATATTTGCCAAGAA
GAAATCAAATCCGCATTACTTCAAGAAGCCTTAGATATCCTCAATGATGG
CAATTTGGAACAAAATACGCCAAGCTATAGCAGTAGATCAAAAAGAGAGG
CCGATGAAGATTTGACAAACGAGGAACGTAGAGTTGCAGGGTGTTTGCTC
CAGTGCGTCTACAAGAAAGTGAAAGCAGTTGATGAAACTGGTTTTCCGGT
AGTCGATGGGTTGATGAAACTGTACAATGAAGGTGTCCAGGACAGAAACT
ACTACATGGCTACATTATCTGCAGTTAGGCATTGTATTTCTATCGCACAA
CAGCTAAAGCAGCTACAGCCCTCCAAAAGTTTCGATGATGGACAAACATG
TGATCTTGCATATGAAATGTTTGAATGCGTCAGTGAAAAAATCGAAGAAA
ACTGTGGAGTCGAAAATAAGTCAAATAACTTAAGCCAACGTCAAGTTTAA

>OBP8
ATGTTCGTTCTTAAAGTGGCGTGTCTGTGCTTGTCCGTCGCCGTCGTTTT
CGGTGAAAACAATCAACAAAACTCAAGTGATCGGTCCGCCACTATATTCC
AGAGTTGCATAGCGGAGACAAAATTGTCCGGAGACGCACTCAAAGGGTTC
CGGTCGATGAGTATACCAAAAACACAAGCCGAAAAATGTATGATGGGTTG
TCTGATGAGAAAAGTAAACGTGATTAACAAGGGCAAGTTTTCGGTTGAAG
AGGCTACCAAGGTTGCACAGAAATATTACGGGACGAACGAAACGATGATG
AAAAAGGCAAAGGACCTCATCGACGTTTGCGCAAAAAAAGCTCAATCGAC
GACTGAAGAGTGTGCGTTGGCCGGAATTGTGACAACTTGTATCGTGGAGG

AAGCTCAAAAAGCGGGTTTGGCCGGCGGACCTGGCAGCCGCTCCAGACGA
ACCGTTTCACCGAAATTCAGACGCAATAGCATGTAA
>OBP9
ATGAAGGTATCTGCAGCGACCGCCGTCCTGGTTGCTCTGGTCGCCACCGT
GCAGAGCTCGGACCCGTGTAACATATCCACTTGCTACAAGAGTGGCACGA
CGAAGCCGCCGATGAATGTGACGCCCACTCGCCTGCCGGTTCAGTCGTCG
TCCACCCCGACCAGCCACCCACAGACCACTTACGCCAAGGACCATGCGCA
CGGTTCGACCACCGTCAAGTCCGGTGCCAATGCTACGGCCACGACAGCCA
GCGGAGCGTCCGTCAACGGTACAGAGCGGCCAGCCGTCGCCAAGTCTTCG
GCCGGAGTAACCGGAAACTCTACCACACCAAAGCCTACGATGACCGAAGG
ACATGTGGCTCTGAAACAGAAGTTGAACACAATCGCGGTTAAGTGCAAGG
ACGAGTTGCACGCCCCCCAGGAGATCATGGCACTGGTCAGCAACACGGTG
GTCCCACAGAACGAGCAGCAAAGGTGTTACTTAGAGTGTGTGTACAAAAA
TCTAAACTTGATCAAGAATAACAAGTTCAGCGTTGACGACGGCAAGGCGA
TGGCTAGGATACGCTTCGCTAACCAACCGGAAGAGCACAAGAAGGCGGTG
ACCATAATAGAGACTTGCGAAAAAGAAGCTGTTATCGATCCGAAAACCAC
TGAAAAATGCGCAGCTGGACGAGTGATCAGAAACTGCTTTGTTAAAAACG
GAGAAAAAATAAATTTCTTCCCTAAAGCATAA
>OBP10
ATGGAACATTTACGTAGCACAAACGTTGTGTTTGCAATTGTAATGGCATT
GTTGGTAGTACAGTCATCTACACGACCACAACCAGATGAATTGGAGGAAA
TAAAAAAGACACTGTACAATGCATGTGCTGGAAAGTTTCCAATTACGGAA
GAAATGAAGAAAGATATTCTAAATTCAAATATGGTAGATGATCAAAATTT
TAAATGTTTTTTAAGGTGTTGCTTTGACGAGATGTCAATGATTGATGAAG
ATGGTATTATCGACGGGGAATCATTGATATCAATGGCTACAGACAATCTT
AAGCCAGTCATTCAGCAAGTCGTTCAGAGTTGTGTAAAAGATATTAAGCA
AGATGGTTGTGAAGCTGCATTCAACTTTATTAGCTGTGGATTGAAATTAA
ATCCAATGACTATTCAACTGTTGCCATTGTGA
>OBP11
ATGATTTCGTCGACGTTTTACATAACTTTGCTGTTCGGTATTGCGATGCT
GATTTCATGTGGCTACGGGCGATTTTCGACGGAACAAATCGATTATTACG
GAAAAGCGTGCAATGCCAGCGAAGATGACCTCGTCGTAGTCAAATCCTAC
AAAGTACCAACTACAGAAACCGGAAAGTGTCTGATGAAATGCATGATCAC
CAAACTAGGACTGCTAAACGACGATGGTTCGTACAACAAAACTGGCATGG
AAGCGGGTTTGAAGAAATACTGGTCGGAGTGGTCTACGGAGAAGATTGAG
GCTATAAACAACAAGTGTTATGAAGAAGCCTTACTTGTGTCGAAGGAGGT
AATAGCGACGTGCAATTACTCGTACACTGTGATGGCATGTTTGAACAAGC
AGTTGGATCTCGACAAGTCAACTTGA
>OBP12
ATGTCCGCTAACTCTGCTACGATCAAGTGCATCGCGGTGGCCGTCGTCCT
GCTTCAAATATCCGTCGTTTTCGCGGATGCGGGTCACCACAGACGGGGCA
AAGAGCTGTTGGACACCGAAGACAGCGACTTCTTCCGGTGCAAACAAGCC
AGCAGAAAGTCATGTTGTGGCCCGGAAAACGCAATGAAGCGATTCGGCGA
TAAAGACAAAGTAGCAGCTGACGAGTGTTACGCACAAGTAGCAGAGAAGT

TTGCAACGGTTGCAGCTACTACTCCCAAACAAGACTTGTTTTCCGCCGAT
GCGGTAAAGATTACCAAGAAGAAGCAATTTTGCCTTCACGAGTGTATTGG
CAAGAAAAAATAGATTGCTCACTGAAGACGGTTCACTGAACAAAACGTTCA
TAGCTGATTACGCAATGAAGAGCGTCTTCAAAGAACAGTGGCAGAAACAA
GTGGGGCAAAAGGCCTTGGACAAATGCCTCGAGGAGACCTACATACCGTG
GCCAGCAGAAGATAAAGAAAACGTGTGTAATCCAGTATACGTACAATTCC
AACACTGCCTGTGGTTGCAATACGAATCGAACTGTCCAGACAACAAGATT
AAGATCACCAAAAAATGCGAGAAGACACGAAACCGATACAGGATGCAAAA
ATCGACATCAAACTAA

>OBP13
ATGCAAAAGGTGGTTTTTATATGTATTTTTGCGATCATCTACCAAACTGT
GTTTACTGTTGGGTACGAGAGAACATGGATTTTACGCCAAAAACGAATGA
CAAATGATGATGAATGCCGAACTCTCCTTCCAAGCTCGGAAAAGAAATTA
CCTTCGTGTTGTCAAATGCCAAATATACTACCCGGGTTGGACAGTACTTG
GGAAAAATGTTATGAGAAATTCATACAATTCAAGGATAAACCTGAAACAA
AAGAATATAAAGAAATGTCACATGGAAAAGAGCCACCATGTCTATTTCAA
TGTATTTTCATGGAATCTGGATTAACTACTAATGACGGAAAACTCAATGA
AGATGCCATCACAAAAAAAAATGACCGAAGGAATAAACAATGATGAAAAAT
GGAAGTCTACATGGAAGAAGTCTCTCGATAAATGCTTTGATGATGTCAAA
CAAGAAGATAAGAAACAAATTCTAATCATGAATACTCCAGCAGGAAGATT
GATGAAATGTTTCTTAAGAGATATATACATGAACTGCCCAGAAAATGTAT
GGGTTGAAAGCTCAGAATGTCTGAATGTGAAGAATTTGGTGCAAAAATGT
CCAGAAATGCCCCCGCCAGTATTCCAATCCGCACCCAAATTAATTTAA

>OBP14
ATGAATAATATGATACCAGCTACAGTTTTGCTCGCTGTTATAGCAGCGAC
CGTCTTAAAGGATTGCGATGCTTACTTGAGTGAAGCGGCCATTAAAAAAA
CACAACAGATGTTGAAAACCGTATGCTCCAAGAAACATTCAGTCGAAGAG
GACGTATTTACTGACATCAAGAAAGGAATATTTCCGGAGAACAACAACAA
CATAAAATGTTACTTTGCCTGTAATTTCAAAACTATGCAAATGATCAATC
AAAAAGGAACCCTCGACAAAAAATTATTCAAAGACAAGATGTCGATGATG
GCACCACCGAACATATACAACATTTTATTACCAGCTATTGAACAGTGCAT
TGGAATAGATAAAGGCGAAGAACTTTGTCAGTCTTCGTACAACTTCATTA
AGTGTGCACACCGCGTGGATCCAAAAAGTTTAGAGTATCTACCACTATAG

>OBP15
ATGTCCGGCATTTCGTGGGTTCTGTTCCTAGGCTGCGTCCTTCTGGGCCT
TGTAAGTCTGGCCGCGGCATCGGAGGCCGACAACAGGGAGAGAGTGCAAC
AGATCTACGCCAAGTGCATGTCGGAGGTGCAGGCAGACGACAAAGACCTG
GAGGGATTTCGGAAGATGCAGATTCCTGACTCCGAAAAAGGCAAATGCAT
GATGGCGTGTCTGATGAAGGAGGCGGGAATTATCGAGTCCTCGAGATTCT
CCAAGGAAGGAGCAGAGCGACTGGCCCAGCGCTACTATGCGGACAACGAG
GATAATATGGCCAAGGCCAGATCTATAATAGACAGCTGCGATAGCTATGT

GGCCGAGGAAACGGAAGAGTGCAACTTGGCCACCAAACTGGCCTTTTGTG
TGGTGGAGGAGGCCCAGAAAGTTGGCCTGTCCTCCGTTCCGGGCGGTTGA

>OR1
ATGATCATTAACTTACAGCTGATTGAGGAAGCTAGTGGAAACAGTATAGG
CTCCTCTGTCATGCAGAAGGTGAACAAGCACGGTCTGGTCGGCGACCTCT
GGCCGAACATTCGCCTCATGCAGCTGACGGGCATGTTCATCCTGGAATTC
CACGAGGACTCATCGCCCGGGACTCAAATGTTGCGACTCGCCTACTGCTG
GTTAGTCACCGTGCTGTTGGTGGCCCAGTATGTCTGTCTGGTCATGTTTG
TGGTGGCTATCGACTATTCTAACGACCTGTTGGCGGGTGGCGTGGTGACG
GCTCTCTTCTTTGCGCATGGCATGATCAAGTACATCTATATCGGACTCAA
GAACAAGTCCTTCTATCGGGTCCTCTCCTCCTGGAATAATGCTAACTCCC
ATCCTGTATTTTCCGAATCCAACGCCAGGTATCGAGCCAAGAGCCTCGCG
CGTATGAAACGAGTACTCACCATCATATGCGTGTGGACAACAGCCACCAT
CGTTGCCTGGGTGACCATCACAATGTGCGGGGACAGTACGTTCCCAGTGC
CGGACCCCGAGGACAAGAACAAGACCATTAGCGTGAAGGTGGCCAGACTG
CCGGTACACTCCTGGTACCCCTGGGACTGCCTCAACAACCAGACGGCCTA
CATGCTCACCTTTGTGTTTCAGGTCTATTGGGTGTTCATGCTCGTCGCCC
ACTCCCAGTTGTGTGACGGCATGTTCTGCTCGTTTGTCCTGTTTTCCTGT
GATCAGCTCAAACACCTGAAGGAGATTCTGAAGCCCCTCATTGATCTGAG
CATCTCCCACCACGGAATACCGAGCTCCCTCGACCTCTTTGTGAACAAAT
CCGCGTCGAGCAACAAAAAACTGATCGCTAACGAGGACTTTGACTACTCG
AGTGTCTACGAGACTCACCACGACTTTAGCAACTACACCCAGAGCAAGGA
ATCCTACCCGGTAGACTCGCAGAAAGAAGAACTCACCCGCTCATCCATCA
AGTACTGGGTTGAGCGCCACAAGCACATTGTGCGCTACACGGAGATGGTG
GGGGACTGCTACGGAATATCACTTCTCTTTCACATGCTGGTCAGTACCGT
CGCGTTGACACTACTTGCCTACCAAGCCACCAAGATTGAAGGAGTCAATG
TCTACGCTTTCTCTACCATCGGTTACTTGGTCTATGCACTGGCTCAAGTG
TTTTTCTTCTGTTTCTATGGCAACGAACTAATTGAACAGAGTTCTTCAGT
GATGGAGGCTGCGTACAGTTGCTCCTGGTACGATGGTTCGGAGGACGCCA
AGACCTTCGTCCAGATAGTTAGTCAACAATGTCAGAAGAGTCTGTCCATC
ACAGGGTCCAAGTTTTTTACCGTCTCACTGGATTTGTTTGCTTCGGTCCT
GGGAGCTGTGGTCACCTACTTCATGGTGCTGGTTCAGCTACAGTAA
>OR2
ATGGGTCAAAGAAACCCTCCATTGGACCGTAAGAACTCCTACATGCAGTT
CAATATGCAGCTGCTCAAAGTATGTCTCCTGTGGCCTCTGGAAACCCACA
ACCCTCTCATACTCTTCTGCCATAGCTTCGTCATATGGTTCACCATGTTC
GTTCTGGCCTTTACCTCGATAGGCCAAGTCCTCAAGATGTGCGTCTCCTT
GGACCTGGCCGAGTTGTCTTCAACCATGGACTTGTTCACTCTGACAGCCT
CCGCCCTCTACAAGATGATCTACCTCCTGATCAATGTGAAGACGATTAAG
AAAATGGTGGATGTGGTCCACAACACATTTGAGGAGGCGCCACTGCTGGG
TATTCACAAATTCCGCATGCAATCTTTTATACAACCGACTCGCCTGTTCA
GCATCATGTACATCCTGAGTGGCCAAATAACGGTCACCTTGTGGTGTTTT
CCGCCACTTCTGTTTGCTCCCCTGGTACCTATCTCGGACCCGAGTGCCCC

TCTCACCATCAAGAACCGAAAAACTATGCCTTTAAATATGTGGCTACCGA
TCAACATTGCTGAAAGTCCCACTTACGAGATCATGTTTGTCCTAGAGACC
TACGCATACTACGCCAGCGCTCTTCTCTACTTGACCATCGACAGCTTCTA
TTTCTATCTCATCTACGTCATCTGTGGACAGCTTAAATTGGTGGATGCAT
CCTTAAGGACGCTGTTTGAGATAAATGAGCGTTTCAGAGAGGAAGAAGAG
TACGCTTTTGTCACTACCACTGAGCAAACTGGACATAATGAAAAAGGTGA
TCTGGTCGTTAAAAAGGAATTCAAAGCTTCTGATAGGCTCAAGGAAAACC
AAAAACTCACCAGACACCGGATATTGAACCAACACTTGGACCGCATTGTC
GATCTTCATGCCATCGTGCTTGACTTGGTACGTCTGATAGAGATCTTCTT
CAGTCAAGCTATTGTTGTGGATTTTCTTCACGCCATTTTGTCTCTAAGCT
TTGCTCTCTTTCAAACCCAGATGTCTGTGACTGTCATAGAGTCGATAAAG
ATGTACGTCTTTCTCGTGGTCTGTATCTCGCATCAGTTCTTCAACAACTT
GTTCGGGGAGCTACTCATTCATGCGCAACACATGATCGTGAATTCGGTTT
ACGAGACACCCTGGTACACCGGTGATCGCAAGTTCAAGAAGACCGTCAGC
ATGATTATCACCCGCTCAATGGTACCTATCAAACTCAGCGGCTTCAAGAT
GTACATCCTCTGCTTGCAGTCCTTCTTGGAGTTCTGCAGACGCATCTTCT
CCTACTACACAGTTCTCAGTGAGGTGTCCAAAACTTAA
>OR3
ATGTCCAAATCCGAAAAGAACAATCCCACCTCCATTCGATTCATCTGCAA
GCTAATGGAAACGATCGGCTACGTAAACTTTCACAACTACGACAGCCCCC
TTCACAATCTCCTCCAGCAAATCTTCTGCTACGTCCAACATTCCATCATA
TTCTTGTTCTTTGCTTTTCAATTTTTGTCCACTCTCACCCGTTCAATCCG
CTACACGCCACAATTCCTGCAAGATCTCTACGTTATGGCGGTAACCTTCA
AAATGATGCACGGGGCTCAACTTCTATTCCACCGCAGCTCTGAACTCACT
TCTTTGGCCAACTGCATGGAGAAATCCTTTAGCAAAGCTGACGCGGGGAT
TGTTAGAAAGAGTCAGCGAAAGGCGGACGCAATTTTTTTCGTCTACGCCG
CTCTCGTCAGTTGCGTCCTAATCTTGAACGTGATTGAAAAGATGCTGCCA
GCTTCCGAAGAGACATTGGAGATCATCAAACGAGTTTACAAGACGTCGCA
TCCTGAAAGGCGCGTCTTGTGCAATATCAAAGTCCCGTTTGTGGACGAGT
CAGAGTCTCCGTATTTCGAGATCATCTTCGCGTGGCACATCTACTTGTCA
TTCATCATGATTCCCAGTTTGGCCACGGCTTTCACGCTTCTGCCTGTTAT
TGTTGCTCATGTGGAGGGGCAGTATGAGATCCTCAGCTTGTTCGTTGAGA
AGATCGGACGCGAACATATGGACGCGAAAGGGAACAAGATATTCTATACA
AGCATTGAGAAGAATGAGTATTTGATTGCTGACAATATGATGGGAGTTGG
GAATACAAAAATCGGAGTTTATAATCACAAGATTGGAGTTGGAATTAATG
CGATGAATTTTGGGAAAGCAAGAAATGCTGTGTCAAATGCGAGGCGAAAC
GCTTATCAAGAGAGGGATGTTCTGTATGAGAAGTTCTTCTTCCGGGAGCT
GGTGAAGTTTCATCAGAAACTGTCAACTTTTGAAACTAGAGTGCTCGAAC
TGTTCAACACCAACATGTTCGTCATGATCATGATCAACAGCATCACCATC
TGTCTCTGTCTGTACCAGCTCACTCTCTTTTCCGACTCTTTCTCCCTATT
GCGTCTCTACACGTTTGTGTCCGAGGTGGTCGCTGTCACCTCCGAGTATT
ACGTCCTCTGCCATTCCTCTGAGGTGCTCGATGATTGCAACGCCAAGTTG
CGTCTGGCCATCCAGAACAGCCACTGGTACGGATGCTCCGGCGAAACCAA
GAGGGACCTGGTGTTCCTCCTGCGGAGACTGCAGAGGCCGAACCACATGA

GGTTCAACCAGGGCGCCATCGTGCTCAGTCGGGTCTTCTTTCTCAAGGTC
ATTCGGGTGTCGTACAGTTTCGTCAACTGTTTGCGACTCGTTAAGTAG
>OR4
ATGGATCTGAATTCAAAGCCTTGGGACGGCTCAGCTTTGAAGCATCGCCT
CGTGACAAAAGTTCAACCTTTGAGTCAGGAAGGTGAAAGACAATGGAATG
ACGATTCTCATCCTCACATCAGTCAGACAAGCTCGAAAGTAATCGAGAAT
CAAAAGAATAAAGAGCTTGAGACACGGAATCCCGGACAAGGAAATGTAGT
TCCGGTTCCTCATGTTAGCTCCATAGTGAACTTGGATCCACACGGCCAGA
AAAATGATTTGGAGCAAATGTTCGCGCACAGCGAGTTCAAAGATTTGTCT
AGCTCTAGCGAGTATGAGTGTTTCCTGGTCAACTTCAAACTCTTTCATTA
CATTGGACTGTGGCGGGAGGATGATAGCTCCCAGCTTTATACTCTCTACA
GTGGTACAGTTTACGCTCTCTTGGTAGTCTTCTTCGTCCTCTCTCTGTTC
CACACCATCCAATCATTCTCCCACTTTGAACTCTTCACTCAGCTCATGGT
GGAGACTATCTTCGTTAGCACTTGCTTCGTCAACATCTCCACCTTCTTTC
TTCTATCCAAGAGGATCAAGTCGTTGCTGCACATCATGAAGAATGATTTT
CTCTCTCTTCAGAGTGACATAACAAGGAATTGCATTCGACTGGAGAAATT
CATCTGCATTGTATCTTGCATTCTGGCTGTTACCACTTACTATGCTTTGG
TCCGGGAGAAAATATTTGGTACTCCTACCATCAGAGAGGTCAATATATTG
AAGAAGATCTACAACCGCACACATCCTGAGAGGAGGCTGCCGGTGCGACT
GTACAGCGGCCCCCTGGACTACACACTCTCTCCCTACTATGAGGCCGTTG
CCTGCTATGACGTCATACTTCTCGGCGTCTGCTTCTACATCCACTACATG
GCCATCTCCACCGTGCCCATATTCTGTGTCCACATCGCGGGCCAACTGCG
GACACTGTCTAAACTGGCGGAAGCTAGCGGCCCGGCGGACCTCGATTCTC
GAAACCTGGCCCTTATCAAACACCACATTGGTATTATAAAATATTTCAAA
GAGTTCGAAACGATTTTTCGTCCGGTGTTCATGATCAAGGTAGTGCTCCG
TCTCATCTTCATTACCCTGGAAATCGTACAACTGTCCCTTCTCAAATCAA
TCACCGACCCCCGGTTCACTAGGACCATGATTCAAATGACCATCGGGTTC
ACCGACTTTCTCTTCTTTTGCGCAAGCTCAGAGGTGGTCAACAACGGGGA
AGAAACATTGTTCCGCTCCATTTACAAGAGTCGGTGGTATGAAACCAGTC
CTCGCATTCGTCAGAGGACCAGTATGATGCTGGAATATTTGAAGCGACCT
CTCAAGTATTCTTTCTACAGCACTTTCCTTATTATAGATATGAGCACGTT
TATTGGAGGCATGCGACTTGCTTATTCCGTGTTCACACTGCTACGTTCTT
TTATAGAATAG
>OR5
ATGTATGTGTTCCTGGCTATCTGTATTCTTCATCAGTTCCTCAATAACTA
CTTCGGCGAAATCATCAAGTATTGGCAAACTCAACTATCGTACTCGGCCT
ATGAAACGCCGTGGTATCTCCGGGATAGGCAATTCAAGCGCTCCGTCCAG
ATGATAACTGCCCGCACCCGCGTTCCCATTATGTTGAACGGACTCAAGAT
GTACGTCCTGTGTCTAGCTTCTTTTGTGGAGTTCATGAGGCGCATATTCT
CCTACTACACAGTTCTGCGGGAGATTTCCAAGTGA
>OR6
ATGTTTGAAAAACGGAAGTTAAGTATGAAATCTTCAAAAACAGTTTATCC
TCATTCAATAGGAGTCTTATACATAGTTTTGGAAGCAATGGGTTTTGTTG
ACAAAATCAAATTTAAATCAGTTTTACTCAACAAAATGAAGCATTTCTAT

TTCATAGGTTTTCATTATATAGCTGTGCTTGCCTTAGTTTCACATTTCAT
ATCGACCATCACTCGGTCTATTCGCTATCTCCCTGAGTTTTTCCAAAAAA
TCTTTGAGGATTTTGGTTGCAGTTCTTTTTACATCGATTGCTTGTTATAT
CTTTTTCGTTACAAAAAACTCCAAGGTATGATGAGATTTATGGAAACGTC
TTTCAGCATGGCAAATGAAGATGTGGTTCGAAAATGTGTCTACAAAGCAA
AAATAACTCTGATTATGTTCACAATTTTAGCTTCAGTTACACTTGCCAGC
CCAATTATAGAAACCTACTTCCCAGTATCAGAGAAAGAAACCGAGATACT
TCGCTATGTTTACCACAGAAAACACCCCGAGAGAAGACTTCAAACCAATT
TGTGGATTCCATTCATTGATGACAGTGAATCTTGGTATTACGAAGCCATT
TTCATTACTATATTCTATTTGATAATTACCATTGTTATATTGGCTGTGTT
ATCAGTTTGTCTCATTATATTTTTGATGATCCACATAGAAGGGCAGTACA
TTATACTGTGTGAATATATTGAGAAGATAGGATATGAACATAAGGATGAA
GAAGGAAATAAAATTATCCATACAAATATAATAAAAGGCAGTGTTCTATA
TCCTGAATACCCTAATAAATTAATTTATAAATGCAATTTGAGATGGAAAC
AAAAGCAGGAAGATATTTATCAACAGAATTACTGTCGCCAAGTTGTCCAT
TTTCATCAGATGCTGATTTACTTTCAAGAGCAGATGGTCAGTCTACAATT
TCCTTTTATGACCGTAAAAGTCATTTTATTCAACACCATGTGTGCTTTGT
GTATGTATCAGCTGACAACCAATCCAACGGACATTTCTCAATATCGTGTC
TTTAAAATGGTGACCGAATGTTTTGCAACAGGAGTCTACTTTTTTACCCT
CTGCTACTGTTCTGAGAGACTCGATGATTGCAATGCGAGGCTACGCCACT
CTATCAGTCAGGCAGACTGGTGGAAATGTTCACCCGGAGTGAGAATGAAT
TTAGTCATGATGCTGAGGGCAGTGAGTGAGCCTAATCATTTGAAGTGGGG
CTATCAGTTTCAGGTGATAAATTATCAGTTCTTTCTGGCGGTTGTGAAAT
CTTCATTCACGTTTGTGAATTTCATGAGGCTGAGGTCAAATGTATCTTTA
AATAATTAA
>OR7
ATGGTGACTGTAAGACACATCCTGAGGTCGTTGCAAGCAGTGGGTGTACT
GAACCTTGGTGACTTCCTCACACAGAAGAGAAACAATATCCACCGACTCT
ACATGGCTGTGCAGACCCTGGTGTGGGTCATCTTCATCTCCACTCACATC
TACAGCACCTTCTCCCGCTCCATACGTCAGCAGCAGGAGTTCATCCAGAT
GCTACTTGAGGATGTGGTGACTATCACGATATTCTTGATAACCATCGAAT
TGAGAGCCAAAGTCCACATTCTAAAATCTTTAACAGATTCCGAGGAGAAA
TTTTTGAACAGAGGTGAAAAGAAGATTCTGCGAAAGTATGAACAACAAGC
AAGAATCTTGTATTTTACTTTTTCTGGTGCAACCGTCAGTTTTGTAATAG
GTATTTACGTCCAGAGAATGTGGCCACTTTCAGAAGAGGACATTGAAATC
CGTCGTAATGTGTACAGGACAGCTCATCCTGAAAGACAGCATCCTTTCTA
TCTGCGCATACCTTTTGTTGATGAATCAGAGTCCTTGGTGTTCGAGATTG
TGATGTTTTTTCTCATTTTCTATGGAACTGTATTTATAATAACTACAATA
GCTGTGATTATGTTGCTCCCTATTGCCACCATACATTTGTATGCTCAGTA
TGAAGTTCTAAGTAAAAGCTTTGAGCAATTCGGTGACTGGACTAAAAATG
AACACACCAACAGACAAGCTGACCTTGATATCTATTATGCTAATATGGAT
AATGCAGTTGACAAAAGATTGGTCCACCTTAATATCTGTAATCCTAATAC
AACCAATACCAATGCATCCACGCCCAATGCATTTAAAATGTTAGCCATGC
GTATGAAAGAAAAGAAAAGACGAGAACATGTTTCCCTCCGACGTTTCGTT

CTGTTACACAACAAACTGCTAGCTTTTCAAAATGAGCTTTTGCGACTCTG
GAGCCCCATTATGTTCGGTTTCATCGTCGCCAACAACATCATGTTCTGCC
TCATCATCCATCAGATAGTGTTTAGCCCCTCGCATCTATCCCCTGCAGTG
CACTTCAAGTTACTGACGCAGCTGTTGGCAGCACCCATGGAGTACTTTCT
TCTTTGCAACTGTTCTGAGATCCTGGACGAATGCAATGCTATCCTACGCC
GAGCGATATTGAACTGTAGGTGGTACAGCTGTTGTGCTAGCACGAGGAGA
GATCTCTGTGTGATCCTGATCAGAGTGCAGAGGCCCAACTACTTGAAATT
CTACAATGGTTCCATCATTCTAAGCAGGGTCTTCTTTATGGGCATCTTGC
GTGTCACCTACACGTTTCTGAACTTCATGAGGCTCAAGAGTTAA
>OR8
ATGGGTGCATCGGCTAGCGCGCACACTGAAACAGCCAGAGATACGAGTGA
GCCGCAAGAGAAGGCGACCATGCTACTGGATCTCATGCTGAACAGTCTCT
GCATTTGCGGCATCTTCAAACTCCCACCCGGTAGATTCTCAGCGCGCGTC
CACCGCATCCACACTGCGTATTTGACCTTCAACCTCTGTCTCAGCTTTCT
GTATGGATTCTCCTGCTGCATGTGTGTCCTAATCTATTCCAGAAGGGACC
TTATCGAATTCTTCAACAAATTCTTGGAAGCGGTTGGCATCAGCGGCGTC
ATTGTGGAAATATCCATCTTCAATATAAACTCTGACAACATCCGCAAACT
ACTTGATCGTCTGAATCAGTTTGACACCAGTAACAGTTTCTTCCGAACGT
CACATCGAATCGAAAAGTGGCTATTATGCATCTTCAGCAGTCTTGTTGCA
CTGATTTTATCTTTTTTAACGATTCGCCCATTTTGTCCCATCGGTCCTGA
ACAGGCAGCGTTTCAGAGAGAGCTGTACGGCTGGAAGTATCCTAAAAACC
GTCTGCCATTTAACTTGTGGATTCCTTACGTGGACACTAGTGAGCCGACT
TACTATTGGCCCCTTTATCTGCTGGAGATCTATTTGACTCTCGTTTTTGC
AGTGGCTTGTATCATGACGATTCAGTTCATACCGCTGACTGTACATCTTT
TAGGAGCGCAGAATTTATGGCTGTCCCAGCAACTGAGAGCTTTGGGGAAT
TCTTCTGAGAGTCCTGTTGATAATATTCATACTCCTGCGAATCCGCTGGA
AAATAGAGGCCCTGTTCAATCTTCTGAAAGGGTCAGATTGATTAGAATTG
CTCAACGTCGTCTGCGTATGAAGCAAGGACAGGAGGTTTTAGACACGAAG
AATTGTGTCTTAATTCACCAGAACCTTCTGAATATTCGAGCAATGTTCGA
ATCCATCTACCGACACAACTTTCTGCTCCGCGTATTTCACTTCAGTATGG
AGTTCGGCATCACTCTCCAATCCATCTCCAACCTGTCTCGCATCTCTCGA
TTTGACGGTGGAACCCGAGTCCTGTTTGTCGCAGAGGCCGCCACCATCTT
GATCAACAACTACTACAACTGTATCGTGTCCGAAGCGCTGGAGTGGTCCA
ATTGGCAACTCCGTCAGGGTGTCTATCGGAGCCGCTGGTACACGATGTGT
CCTCGGGCGAGCCGCATGCTGCTAATGATGCTGCGGATGACTCAGAGGCC
GCAGTATATTCAAACCTTGGGCGGCATAATGAGGTTTGGCAACGAGTATT
TTCTCAACTTGATCAAGAATACTTACGCTTTTATTCGATTTATTGATTTG
AAGGAATCAAAGAAATCTGTGTAG
>OR9
ATGGCATCGGACAAGGACATTTTCATCCTTCGCTGGTACGTCCACTTTCT
TCACTACAGCGGGTTTTTCAGTCTACCCAACTTCTATCGGGAACCCTGGA
AGAATCGCCTGCATCGCTACTACTCTTTCATGGTCGTCACAATCGTCCAG
CTTTACGCCATGAGCATCGCTACAGCGCTGTTCATTTTCGCGGTCCGTGA
TTCTAGCGAATTTTGTGAGCGAATCTTCGAGACTCTGATCTGCGTGTGTT

GTTCGATGGACGTGCTACACATCCGTTGGAACCTGGGCTCCTTTCTAGAT
TTACTGGACGAGTATGAAGGCTTCGCCAAGCCGCATAGACAGCGGCCGGA
TCTTGTCAACCTCAGACGAGTGGAATGGCGTGTTATGCGAGTGGCGTGGA
TTGTGTTGGCTGTGGAGAATTCAATTAACTCCTACTTGTTGCCTCGGATA
CCAAAGTCTGCAGAGAATCTGGACCGTCTCCAGAGAATCTACAAGATCAA
GTATCCCCAAAACATGTTCATTGTCCCTATGTACATCCCTTTCGTTGACA
CTAGCGAGCCCACCACGTACACACTTCTCCTTATCTTCTTGGCTTACGTC
TTCTTCCTCTACACGTCCACATTTATGGTAGCTGTCCTTTTCTATCCGGT
GTTTGTATTTGAGATGAAAGTCTACACCGATATTTTGTGTGACTATGTGA
GACTAATTGGTCATGAACACAGAGACGAGAGCGGTAATCTGATCTTCTAC
ACAGATTTGGCCATAAAGAGTTTTATGCGGCGAGAGACTCTCCCTAAAGT
CTCCCACTACAACCACATTAGAAACACTCAAATAAAACACCTGAACTATT
CTCACTACAACAACATCAAGAATAGTTACATGAACGATATATACTACAAT
CACAGCAAAGATACCAAGGATCTAAATCAAGAAGAACAAGCCGTTATGTA
TGTGTTTGTCCGGCGCCCGAAAGTGTATGAATATTTCTATCTCAGTCAAG
TGATTTTGCTACAGAAAAAGTTGTTACTTATTAGAGAAAAGCTGGACATA
TTTCACCACCGCGCCTTCAAGGTTCGCTGGACGTTGGCCAGTGTAATGAT
AGTGGTCAGCATGTATGGCTTGCTAACGCCGGACTTGCTGTCCCCTATGA
TCCGGATCAAAGCTACCGGTGAAATCCTGTTGGTTCTCGGCGCCTACTAC
GGGCATATGTACTGCGGAGAGATGTTGGCCGATGTGAACGCCTATATACG
ACTTGCGGCCTGGGAGAGTAAGTGGTACCGGACTGCAGTCGGCACGCAGA
GAGGCCTCATGATGCTGCTGCGAATTACCCAGCGGCTCAAATATGTTAGC
ATCTTCAATATGATGAAACTCAACTATGGCCTGATGATCAAAACATTCAG
AGTTTCCTACTCATTCTTCAACTTCATGAATATGTCTCGTGAGCGATAA
>OR10
ATGACGTACGAAATTTTTACAAAAACAAACAATATCTTCAGAGCGGTAGG
GTTGTACCCGTTGGTGAATAGTAGAGTAAGGCAAGTCTACGCCTATTTCG
TGTTGAGTTTCATGATCTTGCTGACAATTCTGTATGTGGTTGAGTCCTCG
CGGGGAGGCTTGAAGTCGCTCAACTATGAGGTCTCATCCCTCGTGTTCAT
TATATTCATCAGCTCTGAGTTCCTCACCAGCTTGTTGCGTTTCGGCCAGA
GGGCGAAACTGAAACGCAAAGTCGAGGAAAGCTTTTGCAGCACCTTCAAG
GAACGACTTCGGAAGATCGAATTTATGGAGAAGGTTCTAATTTGGTATTG
GCTCGTCCTTATGATATTAGTTCATATATTCTGGCTGCTTCTTCCCATGA
TCGGAAATAGGAAATCCGATTACATACCGTGCAAAAGAGTTCTGCCGCAG
ATATTGTGGTCCCCTTGGTTCGATCTTAGCACCTATCCCCAATATGAGAT
AGCTTACGTGGTGGAAATTTACATTACCATCTATTACGTTATCATCAGTT
TCGATGTTGCCACCACCATTCCGATTATAGCTCTGCATACGTACGGCCAG
ATGGAAATAATTGCATTCTACTTGAAGAAAGTGGGGAAAACTGATTTTAA
AGGTTCCCGTGAAGTGATCTACACCAACATTCAAAACGGGACCTACGTGG
TGGTCAACAGGAAAATCAAAACCACCCGCCTTCACAATGGTCATATTGAA
ATCCCTGCGCCCAAGATAGAAGCCTACATGGAGATCTTTGTCAAGGATAT
AATCAGATACCACAGCACACTCCTGTCGATCATCCACCAGTACGCGCGTC
TCAACCACGCCAACATCATCGAGCGGTTCACCGTGGTCACCGTTCTACTC
ATCACAGTCACCACCCAGATCAACAAGACCTACTCTCCTCCACTCGCTCT

CATGTTCATCGTGGGCATTGTCGCCTGGGAGTTCAACCTGTGTCTCAGCA
GCGAACTGATCGACTCCGGGAATCAACGAATCTACGCGGCTACGTACGAC
AACGCCTGGTACAGATGTGGCCCGCGCTTGCGCAAAATGCTACACTTTAT
GATGGTGCGCACGCGCGCTCCCAACCATATTAAACTGTTCGGGTTCATCG
TTGTGTCGTTCACGTTGATTCCCGAAACGTTGCGTTTCTCCTACTCGGTG
TACATTTTGCTGGACCAGGGCTGA

>OR11
ATGAAGATGGTGGATAGCTTGTACATGAGGCAACTGGTTATCTTCCATCA
GAAACTGCTCGTCTTCAATGAGAAGTTCTACAGGGTGTTCGCTACAATGT
TCACTGTGCAGACAGTCAGCAGCATGCTGATTCTAACCCTGACTACGTAC
CAAGTGCTTCTGCTGAGCGGACTCTCAGATCAGCAAACTCCCAAGGTCAT
GGGCGATCTCGTTTGCTCCATGGGCTTCTTCTTCTACTTCAGCAACCTCT
CCGAGAGACTCGAGTCAGCAAATGTCGCCAACAGAATCAGCGCTGGTCAA
AGCGCCTGGTACAATGCGCCCCCGCACCTTCGACGATGTTTTCTTCTCTT
GCTTCTGCGCACTCAGAAGTGTGCACATCTGAGGACGATGCATGGCTTGA
TACGCGTCAACCATGCCTCGTACTTGCAGAGTCTGCAAGTCTCTTACCGG
TTTCTCAACTTCATGCGAATCAATGCCACCTGA

>OR12
ATGGTATCCGCTCGGACCCTCACCATCTGGCTGGAGCGCTTTGGCATGAT
CAACCTCCACCGCTTTCCTCTGCTCTGGCAAGATCACCTGCAAACTATCT
ACGCCTCCATTCAGACTAGCGTTTTTTTCCTCTATGCGATGGCGCACCTC
TACAGCACTCTGACCCGCGCCATCCGCTACCAGCCAGAGTTCATTCAGAC
ACTGCTAGAGGACGCCATCAACATTTTTCTTTTGTGTAACATCTGCTTCT
TCCGCTTCATGTACACCGAGCTTAACTTCATAGCCGACACTTTAGAAACC
TCCTTCAGCCGAGCCTACCCGGACGTTAACGATATGTGTGCGAGAAAAAG
TCGAAGACTCCTCATTTTTTTCTTCTCGTTCGTTTTCGTCGCAATTCTTG
GTAGTTTTATCGAGACAATACTGCCGCTCGTGGCGAGTGAACTGGAACTA
CGCCGGGAGATCTACCGCACGAAACACGCCGAGCGGCGACTACCATATAG
CGTGCGCGTTCCGTTAATCGATGAAACTGAGTCGTACGCGTACGAGATCG
TTTACGGGACGATTAGCTATCTTATGTGTTTCTTTCTCGTTTGGGTTAGT
ATTAGCGTTAGTTTGGCGCCAATTGTATTAATTCATCTCCGCGGTCAGTA
CATGATTTTGTCCCGGTTTGTAGCGCTCATCGGTAGACAGCATCGAGATA
GGTATGGCTACCGCATTTACTACACGAGTATTGAGAAGAATGAGTACGTG
ATCAAGATGTACGGGAACGTGGCTAGTCGTCAGAGTAGGATGTCGCAGTG
GGCGATGGAGAAGCGGTACGAGCGACAGTTCCTACGGCAGATTCTCAACT
ATCATCGCAAGCTGTTGGTGTTTCAGTCTGAGATGATCACCATCATCACA
CCATTTGTATACTGCGTCTTCGTCGCAAACAACATCATCATCTCCCTCAG
TCTTTACCAAATTGTTGCCAGCCCCAATAAGCTCACCCCTTTACGCTACT
TCAAATTCTCCGCCGAACTGATAGCCGCCATTATCGAGTACTTTATCTTG
TGCAACTGTTCGGAGGTCGCTGCGGAATGTAACGAACTAATCCGAAAGGC
ACTCGGCAATTCCCGGTGGTACTCGTGTACCCGAGAAACCCGAAAGGATT
TGATCATGTTTTTGAGACGTGTGACGCAGGAAAATCATTTGCGATTCTTT

CACGGTGCGATAGTTCTGAATCGAGCTTTACTTTTGAATGTGATGAGAGT
GGCCTATACGTTTGTCAACTTCATGAGGCTGAGGATGAAATGA
>OR13
ATGACTTTCATTTCAATGGGCATATCAGTGCTAGGTAGACTGTTGGAAAC
AGTCTTACCTCTTTCGCTGGACGAGCTCGCAATACACAACTGTGTCTACC
AGAACAAACATCCTGCGAGAATATTGGTGTGGATTGTCAAGATACCTTTT
ATCGATGAGACCGAGTCACCCTACTACGAGATAATCGCCTCCGTCGAGAT
CTTTTTAGGTGCTGCATTCATTATTCTTTCCACGTTTTTTGTCACTCTTA
CACCGATCAATATCACCCATCTCGCAGGACAATACATTGTTCTATGTGAA
CATGTTAAAAACCTCGGGCGGGTTCTAAATGACTCGCAGGGACAAAGGAT
CTACTACAGAGATATTGAGAAGAATCAGCTGACTTACTTCAAGTTCCCGT
CAGAAATGACATTGTCTGCAAAGGACATGAGAAGTATTATCAAGAAAAAG
AAGGAAAACCAATATGAACAGTTCTACATTAGACAAATCATTCGATTCCA
TCAGAAGTTATTGGACTTTCAACTAAAAATGCATGCTTTTCTACAACCCC
TCATGATTCCGACAATTTTTTCCATGTATGGCGCCTTCAGTTTTTGCCTG
TATCAGGTGACAGCCGTCAGTTTTCAGCTGTCTCTTGTGAGAACCATCAA
GTTTGTCTCTGAGTTCATCAGTCTCGTCATATGGTTCTTCATGGTCAACA
ACTCCTCCGAGAAGCTGGACGACTGCAACTCTCTCATGTGCTCTGCTATC
ACTTCAAGTCAATGGCACCTCTGCTCTCCAAACACAAGGAGGAGTCTGCT
ATGCGTTCTGCGCAGGTCACAGAGTCCCAATCATCTCAAGTTCTACGGTG
GCTACGTCATACTGAGTCGGGCCCTTTTCCTTCGGATCATGCGCTTTGCG
TACTCGGTAGTCAATATCATGAAACTCAAAACCTAA
>OR14
ATGGTATCAGTGGGATACATTCTGAGGACGATACAAGCTTTGGGCGTGCT
GAACCTGGGTACCGCCCCCAACCAGACCAAGAATGATCTGAAAAGTCTAC
AAGTGGCCTTGAGTAAATTACTGTGGGTTTTCATCATGTGCACACACTTC
TACAGCACCTTCACTCGCTCCATCCACTACCAGCCAGAGTTCATCCAGTG
TCTCTACGAGGACGTCGTATTCATCACCATATCCATGATACTCATAGTAC
TGGAAAAGAAGGTCCCAATGCTCAACTCCCTGATAGATTTCGAGAAAAAC
TTTCTGCAAAAAGGGGACGCAAAGGTTTTACGCAAATTCCAACGCCGAGC
GAAAATTTTGTACATCACCTATTTCATAGCAACAAGTGCTGTGGTAACAG
GTATATGCCTCGAGAAAATGGTTCCACTTTCGCCGGAAATCTTCGAGATC
CACCACAAGATTTACAGGACACAGCATCCCGAGAGACAACACCCCACTCA
TCTCCGCATCCCTTTCGTCGATGAAACGGAACATGGGGCTTTTGAACTTT
TGATGGTTTTCATCCTTATATTCGCTTTTCAATATGCAATAACCACCACT
GCTGTGACTATGATCCTCCCCATCTCTGTCGTACATCTGTGCGCCCAGTA
TGAACTGCTGGGTCAATGCTGTGAGCAATTGGGCGACTGGCCTGGGAGTG
CACCTGACGGAGGACCAGCTGGGAATGCAGATGCACCTAACGAAGGACCA
AATGATCAACACACTGGTGCTGTTGCATATGATTCCAATGCATCTGGTGG
AAATGCACTCAACATGAAAACAAAGACAAGAGACATGTTGGAGGAGCATG
CCCGTCTCCGCCATATCGTCGTCTTTCATAACAAGCTCCTGGATTTTCAA
AATGAGCTGCTCAACCTCTGGAATCCGATCATGTTCGCTTTCATCGTCGC
CAACAACATCATGTTCTGCCTCATCATGCATCAGGTAGGGTTCAGCCCCG
TCAAACTCTCCCCAGCCTTGCACTTCAAGCTGCTGATGCAGGTGTTGGGC

GGCTGCATGGAGTACTATCTTCTCTGCAACTGCTCGGAGACCCTGGACAA
TTGCAACGCTGTCCTGCGCCGCGCCATAGACAACTGTCGGTGGGACAACT
GCAGCCCTGACACGAGAAAAGCGCTCTGCGTGATTCTGGTCCGAGTCCAG
AAGACCAACTACTTGCAGTTCTACTACGGCTACATCGTTCTCAGCAGAGT
TTTCTTTGTGGGCGTCTTGCGGTTTGCATACTCCTTCTTGAACTTTATGA
AACTCAGGAACTCCCGGCAGCAGCAGCAGCAGTGA
>OR15
ATGATTGTCACTATCGAGTACATCTTCAAAGTCATCGAAAATGTTGGTCT
CATTAATTTGAAGAGCTTTCCTACCAAACGGAGAGCCACTCTACAACGCA
TTCACGTCGCCATTCAAAGCGTCCTATGGTTTATCTACATGTCCAGCCAC
AGCCTGAACACCTACACCCGGGCGACTCGCTACTTCCCTGAGTTCTGCCA
GATGCTCATGGAAGACTTGGTCGGACTGGAAGTGGTGATCATTGCGTTTC
ATCTATCCCACAGGACGCAAGAACTGAATCGTCTCATCAGTTATGAGCGA
AATATTTTCAGCCGCGCCGATGGAAAGATCCTGGCGGAGGCCCAGCACCA
TGGGAATCTCACGTACTTCGCCGTGTTTACCGGTATAGCGCTAGCCACGG
CGGGGGCTAATTTGGAAGCTATCCTACCCATTGCCCCCGCAGAGCTGGAG
ATCCGGCGCAGCACGTACAGGACAGAACATCCAGAACGTCGCCACCCTAT
CACTCTCAGGATTCCTTTCATGGACGAGTCCAAGTCCTGGACTTACGAGG
CCCTTTTTGTGTACATTGTCTACATGGACACCGTGTTCGTATTCGCCACG
AACGCTATCATCACCCTGCTACCCGTCGCTATCATACATCTGCGCGCACA
GTACGAGATCCTTTGCAAATTCTTGACAATGTTGGGCGAAGAGCATGAGG
ATTTGGAAGGGAACAGGATTTTCTACACAGATTTCATGAAAAACAAAAGC
ATATTAGATCCTCGAAAAAAATGCACGGGGACAATGCCTTCAACCTCAAA
GAATGATGTGAGGATTCGACAACAATACGAACAGTTTTTTCTTAAGCAGA
TTGTTCAGTTTCATATGCAGCTACTACGGTTTCAAGATGAGCTGTTGAAG
CTATGGAGTCCCGTTATGTTCGTGTTCATCATCGCCAACAACATCATGTT
CTGCCTGTGCCTCTACCAGATGGTGTTGAACCCCAACACCCTAGCCCCAG
TGAGGTATCTTAAGTTCACGGGGGAGATAGCCGCTGTGGCCATGTCCTAT
TTTCTTCTCTGCAACGCGTCGGAGATCATGGATGACGGCAATGCCTCCAT
GGAGAGAGCCATTTTGAACTGTGCCTGGCACAAGTGTTCAGGCGAAACAA
GACGAGACCTTGGTGTCATTCTAAGGAAAGTACAACGCACGAATCATTTG
AGATTCTACAAAGGAGCTATCATTCTGAGCAGGAAATTCTTTCTGGGGGT
TCTTAGGCTGACGTATTCCTTCTTTAATTTTATGCGGCTCAGGGGTGCTG
CACATTGA
>OR16
ATGTATGCTGAACAATTAAAAGAATGGAAAAATACATGCAATTTGAAACC
TATCAAAGAAACGGAAGATATCTACCAGTGGGATTACTGTTGTCAAGTCA
TCCGATTTCATCAGATGTTGATTAGCTTTCAAGAACAGGTCTCCAGCCAG
CTGCACACTTACTTGGTCATCAAAATTGTTTTCATGGACACAATAGTCGC
TCTGTGCATGTATCAGCTCACGACTAATCCCACAGATATTTCTCGATTTC
GTGTTTTTAAATTGGTCGCCGAATTTATTGCAGTAGCAGTTGTGTTCTTC
TCTGTGTGTAACTATTCTGAGAGACTGGATGATTGCAATGCAAGGCTACG
CCACTCTATCAGTCAGGCAGACTGGTGGAAATGTTCACCCGGAGTGAGAA
TGAATTTAGTCATGATGCTGAGGGCAGTGAGTGAGCCTAATCATTTGAAG

TGGGCGTATCGATCTCGTGTAATAGGTTATCAATTATTTTTGTCGACTGC
CAAATTTTCTTTTAACTTTGTGAATTTTATGAGGCTCAAGTCAAATGTCT
CTTTACGTTGA
>OR17
ATGATTACTATAGAAAACATTGGCAACACACTTGAAAAAGTAGGTTACAT
TAATTTGGGAAGATTTTCAAAAAAGAGGAGGATCATCCAAATTATTTACA
ATGTGGCACAACTGGTATCGGTCTTCCTGTTTGTGAGCTTTCAATTATTC
AGTACCATGAATAGATCCATCAGGTACATGCCCGAGTTTTTTCAAAGGCT
TGCGGAGCTTGTAGTCAGTCTGGAATATTTTATTATAATATTCTACTTGG
CAACCAAGTCCAAAGATATCAACTCTCTAGTTGAATACGAGCGGAAAGTG
TTCATTAAATCCTCCCACTTTGAGATTCTCAAGAAATGTGAAAAGACTGG
TAAGATGATGTACTATACTGTTTTTTGTGTGATAGCTGGGGCCATGACTA
CAATTTACATAGAATCTTTCTTTTATCTGCCAAAATCTGAATTGAAAATT
CGACGCCACGTCTACAGAACTAAGCATCCAGAGAGGCTTCATTCGCACGA
GATTCAGATTCCATTCATTGATGAAACCGAATCTTATGCCTACCTATTAA
CAGTGATATGGCTGGTGATCGTAGATATTACCTTTGTTTTTGGGGCGGTT
GCCATAATTAACTTGATTCCAATTGCATCATTTAATTTACGAGCTCAATA
CGAAATTCTCGCTAAATACCTATCGCAAATGGGAGATCAACAAAGAGATG
ATAAAGGGAATGCCATTTCATACACAGATCTGAAGACAAATGAATTCATT
TCTCATCCCAACGTACCTGAAAGAAGATTAAAACTGATTGTGCAGAGACA
TGAGCAAAATCTCTTGAAGAAGATTATCAAATTTCAGAATCAGCTGATCC
AGTTTCAAGCAGAGTTGTCACAGTTGTACAGCCCCATCATGCTCCTCATC
ATAGTCTCAAACTACATCAAATCATGTCTTTGTTTCTACCAACTTGTGTT
TAGCCCAGTGAAGCTACCTCCCTTCAGATTCTTTAAATTTCTTCTCGAAT
ATGGAACTGTAATTGTGGAGTATTTCATAGTTTGTAATTTTTCGGAGGTA
TTCGATGACTGTAATGGCTTGGTGAGGCAGGCAGTATGCGACAGTCAATG
GGGTAGCTGTACGAACGAAACGAGGAAATATGTTCTCATGATTCTGAGGC
GGGCGCAGCAACCCAATCATTTGAAATTTTATAAGGGAGCTATCATATTG
AGCAGGAAATTTTTCATGAATGCAGTCAATGTGACGTACACTTTCTTCAA
CTTCGTTCAGTTGAAGAAAGGTTTGACTGTTGAGTGA
>OR18
ATGTATTCAAAATTGAAGGAACCACCATCCATATCATTTCTTATAGCCCT
TATGGAAAAATCAGGCCTAATTAATTTTCACCACAGGGAACAAAACTGGC
AAAACAAAGTACAAGAAATTTACTGTTGGATTCAAACACTGCTTCTGACA
ATTTTTCTATTCAGTCATCTTTACAACACCGTCACAATGTATGGACAGTC
TGAAACTGAATTTTTTCGATGCCTTAATGACGATTTCTACATTATTACTT
ACTATATAAAATACATTATCTTGCAGCGCAGATCCAAAGACTTGAAGGAA
TTGGAATACTTTATGCAAAACTCTTTTAGTCACGCAGATTCCAAAGTTAT
TTACAAATATAGCAGGAAAATGCATGTTTTCCTAAAGACATTGGTATGTG
TCTTTTTTGTGCTTTTCATCGCAAATATGGTTGAGATACTGTCACCTGGT
GGAAAGGATTTTCAACAAAAATTTCCATTTGTTGACACAAAAGATCTGAG
ATCATACAAGATATTCATCCTATTTATCTTATACATATCTTTTGATTATT
TCATCTTGCTAATCGTGTTTATCACTTTTGTTCCATGTGTCATATTTGAC
CTTATCGCTCAGTACACAATATTGTGTAACTACACTGAAAAACTAGGTGT

CCCACATTACAACAAGGAAGGAAAAGAAATCATCTACATGGATATTGAAC
AGAATAAGTATGTCCTTCGTTTGAAAACATTGGGCAAAGTTGAAAGAAGA
ACTAGTTCGGGATTTGGTGGATTCAGTCATCAAACTTACAGTCCACAGTC
CTGCACTCGAGAAAGACGAATAAATGAACAGAACTATTTGAGGCAAATCA
TAAAGTTCCACCAGAAACTCCTTCATTTCCAGGTACAGTTCATACAGTTC
TATGGTGTCTGCATGTTGTGTCAAACAGTCCTCAACTTTTGCGGCACTGC
TCTGTGTCTGTTTCAAATAGTCAGCAATCATGGATCCGTCTTTCAACCTC
GGTTTTATGCCTTTATGTCCTTTTTAGTCCTTATTAATATGATGAGCTAC
TACTGGTGCCACTGCTCTGAGCTTCTTGATGACTGCAACACCAAACTCTG
TAGGTCCGTGCGAAACAGTGAGTGGATGAGGTGTGACTTGGAGACGAGAA
AAAACATATGCTTCCTTCTTCGAAGAGCACAAAAACCAAATCATTTGAGA
ATTTGCCAGGGCATAATTGGCTACTGTAAAGCTCATTTCTTGAGGATCAT
ACAATTTTCGTACACTTTTGTTAATTTTATGAGATATGCCAACAAGTTTT
GA
>OR19
ATGAAGAAACATATTATTTCACATCAGGAACTACTGGAATTCCGAGCTCT
GCTTAACTCTATCAGCAGTCGCAATTTCGTCATCCGCATCATCCTGGTCA
CCTTCATCAGCAGTCTCTCTGCTCATCCCATTTCGATTCTGTCCGAGTTC
GACAGGAAAAAGCAGATCCTATTTGTCGGCGAAGCTATACTGGCTCTGGG
GTTCTACTTTTATAACTGCCTCTTGTCGGAAACTCTAGAGTGGTCGAACG
ACATAATCCGACAAAGTATCTACGACTCCGGATGGTACGTCTTGTGTCCT
CAGGCAAGACGCATGATACTCATGTTTCTCAGGCGGACACAGAGGCCGCA
GTATATTCGAACCCTGGGTGGGGTCGTGGTGTTGCGCAATGTCCATTACT
TGCAAGTCCTCAAACATATTTACTCATTCATACGATTCATCAATTTGAAG
AACCGGAGAACTGGTTTTTAA
>OR20
ATGCTTCGAAAACGAATCGCATTTGTCAGATCTGAAGAAAAAGTCTACCC
TCATTCAATAGGAATATTGTACGTGGTTCTTGAAGGCATGGGTTTGGTAA
ACAAAGTCAAATTCAAATCGGACACACTCAACAGACTGAAGACTGTCTAT
TTCAAACTGTTTCATTTCACTTGCCTGGTATCTCTGACCCTACATTTCCT
ATCGACCGTCACTCGTTCTGTACGCTATCTCCCTGAATTTTTTCAAAGAC
TCTTCGAGGATTTCGGTTGCAGCTGTTTCTACATCGACTGTTTGCTGTAT
CATTTTCATTACGAGAAACTCCAAGACATGATGAGATTTATGGAAACATC
TTTTAGCATGGCAAATGAAGATGTGGTGCGGAAAAGTGACTACAAAGCAA
AAGCAACTCTGATCATTTTCTCATTTATATCTACTATCGCGCTGTCTGGC
TCAATCATAGAAACCTACTTCCCGGTGTCCGAAGAGGAGATAGAGATACT
GCGCTATGTTTACCAAAGAAAGCATCCCGAGAGAAGACTTGAAAGCAGTT
TTTGGTTTCCATTCATTGATGAGACCGAGTCCCCGTTTTACGACGTACTT
TTCATCGTTGAATTCTATTTGGTATTCGTCATGATACTTGTGGCGATGTT
ATCAGCAGTTCTTATTTACTTGTTGGTCATCCACGTCGAAGGGCAATACA
TGATACTGTGTGGATACATTCAGAAGATAGGACATGAACATAGGGATAGA
GAAGGAAAGAGGATTTGTTATGCAAATATAATAAGAAGCGACGTTCTGTA
TATTAATGACCTTAAGACACCTATAAAAAGGCGCAATCTGAGATGGAACC
AAAAACTTGAAGATATCTATCAACAGAATTACTGTCGACAGATTATCCAA

TTCCATCAGATGCTGATTCACTTTCAAAAGCAGATGGTCAGTTTACAATT
CCCTTACATTACCATGAAGGTCATTTTCTACAACACAATGTTAGCTCTGT
GTATGTATCAGCTGACAACCAATCCAACCGACATTTCTCAATATCGTGTA
TTCAAACTGGTCACCGAATTTCTCGCAGTAGGAACCATTTTGTTGGGACT
CTGCCACTGTTCTGAAAGACTGGACAGTTGCAATAAGAGGCTGCGCCACT
CCATCGTCCAATCAGATTGGTTGAAATGTTCGTCCGCAGTGAGACTGAAT
TTGGTCATGATGTTGAGGACGGCAGGAGTGCCTAATCATTTGAAGTGGGG
CTACTGGTTTCAGGTGATTGACTATGAATTCTTTCTAGCCGTTGTGAAAT
TTTCGTTCAGGTTTGTGAATTTCATGAAGCTGAAGTCAAACTATAATAGT
GTTCAAGTTTAA

>OR21
ATGAAATCTTCAACGCCAATAATGACTTTCAGGGATATTGGCACAGTGAA
AATGTTCCGTATGACTTGCAGAATATTGGAACTGTCTGGACTCACCGATT
TTCACTGTTGGAAATCTCCACTGAGGGGTCATCTTCAGAAAGTATACGTA
TGGTCCATTTTCATCCTGACATATTTCTATCTCGCTTGTCACGCCTGGAA
CACAGCCACCCGGGCAACACGCTATACGCCGGAATTTCTGCAGTGTCTCA
TGGAAGACTTGGTGTTGTACATGACGGCGCTCAACGCCTTCATTCTCTTC
ACTCTCCAGCCGCATAAGTTTGTCCATCTGATAGAGTCCATGGAGAGGTG
TTTTATCAAAGACACCGAGGTGTCTGACCGCTGTGCGATCCAAGTGAGGA
ACATTATCATCACATACATCACCTGCTGCCTCATCACAATGTCAACAGTC
ATCTTGCAAGTCCTCGTCCCTCTGTCGGAGGAAGAGTTTAAAATTCGCCA
GTTGGTGTATGCTGACACAGCACACCCTGAAAGGATTCTGCCCACTATTA
TCAAAATTCCTGGGGTCGATGAGACAGTTTCCTGGAACTATGAGCTTGTG
CTTGGCTATCAGATTTTCCTCTCGTTGATTTTCGTCATCACATTGATATT
CACTGTTTCATTGTTGCCAGTTATTGCTGTTCATTTGGAAGGGCAGTACA
GGATTTTAGACCAGAATTTTCAACTCTTAGGCCGAAAAGACAATTTTCTA
AACATAAAAGGATTTGATTTAAGAAGAAGCAATCATCAGAAAAAGAATTT
TAATAAATACCGTCCTAATATCAGCCGCGTAGTATGCAATACATTAAAAT
TTCAAGAGAAAAAGCAATGGATTGAGGATATGCAAAGGAATCTAGAGTTA
AATCTTTTGCGAAGAGCCATCAAAACACATCAAAAGCTTATTCAATTTCA
AGAACAGATGAAAGTCTTCTTCACACCCTCCATGATCCCCATATGTGTCA
CCAACTACACCTGTTTCTCGTTGAACATCTACCAGCTGGTCGTCAACCCC
TCGGCTATGTCGAGTATCCGCTTCTTTCGGTTCTTCTGCGAGTTGGTTAT
TCTCAGCACAGAGTTTTTTCTCGTCAGCAACGCCTCAGAGATAATTGATG
ACTGCAGCCACACCCTCTACCGCTCCATCCAGAACTGCAACTGGTACAAC
TGCTCGCTACAGACAAGACGCAATCTTTGTATGATGCTGCGGCGGCTGCA
ACGTGGCAACCGGTTGAATTTCTACCAAGGGCTGGTGGTCCTTAGTCGAC
AGCAGTTCCTGAGGGTCTTGAGACTGGGGTATACCTTCGCCAACACTATG
CGAATGAAGGCGAGCACCAAACTTTGA
>OR22
ATGGAGGAGCCTCATGATCAAAACACGACAGACCCTGATCCGAGAGACGA
AGATCTGGCTCGTGTGGAGATTCTCACACTTCTCAACATCCTGGTGATTA
CTCTCATAGGTAACCTCATGGTCCTGGCTGCCCTCTACTCCCGACGACAC

TACCGAAAGCAAATCAAAATGCCACGGATGTACTACTTCATCCTTCACCT
GAGTATTGCGGACCTACTGACCGGTGTGTTCAATGTTCTACCACAACTCT
GGTGGGATATCAACTACCGGTTTCCAGGGAGTACGAGCTGGTCCTGCAAG
TTTGTCAAGTTTGTTCAACCGTCTGGTTCATTCCTTAGCAGTTATATTCT
GATGGCCATCGCCATCGATCGGTATCGTGCGATTTGTCATCCTCTCACGT
ATCATGCTTGGAACTCCAGGAGATCCAGAATTATGATTATG
>OR23
ATGACGTACGTGACAGTTTGTGTGGCTATGGTCACAGCCATTCTGTTCCA
AGCGTATGTGCCTCTCTCAGAGGAAGAGCTGAGCATGCGTAAGCGGGTGT
ATCCTGACGCAGCAAATCCTCAGAGAGTTCTGCCGACTGCCGTTAAATTC
CCTGGCATTGATGAGTCGGTGTCCTGGAACTATGAAATCATTCTCTGCAT
GCAGCTGTTCTTGGGGTATATTTGCATCATCACCCTGGTTCTCACGGTTC
CTCTGACTCCAATGATTCTTGTGCATCTGGAAGGGCAGTACAAGATTCTA
AGCAAACGTTTGAGAGTTTTAGGAAGCCGACAACAAAATTATCCAAGCAC
GAAAGAATCCAATACGGTGCACTACATTTATTATGAAGATTATGTGAAAT
ATTGGGCCATGAGCCGTCCAGTTTACAGAACACCAAACAGAGACATCCTA
CAACAGATGAAGATGGAAAGACGGCAGTGGTATAAAACGATACAAAATAT
TCGGGAATTCAACGTCCTACGCCAGATTGTTCAAATTCATCAAAAGCTTA
TCCAGTTTCAAAAAGAGCTGAAGGACTTCTTCTCGCCAATCACAATTCCT
CTCTTCGTCATTAACTATACCTGCTTCTCTCTAAGCATCTACCAGCTGGT
TGTTAACCCATCCACCATGTCCACTCTCCGCTTTGTTCGCTTCTTGTGCG
AGTTCTTGGCGCTGATCATTGAGTACTATCTCCTCACCGACAGCTCAGAG
ACGGCAGACAACTGCAACCAAATCCTCCTTCGTTCAATCGAGCAAAGCAA
TTGGTACAACTGTTCTCCGTCTATAAGACGCGATCTCTGTATGATTCTGC
GGCGCGCGCAGCAATGCAACAAGCTAAATTTCCATCAAAGGATTGTCGTG
CTCAGCCGTGTTCAATTTCTCAAGGTTCTTAGAGTGGGATATACTTTCGC
CAACACCATACGAATGAAATCTAGCCTTAGCATTTGA
>OR24
ATGGAGAGACAGCAGTGGTATGAAAAGATGCAACATTATCAGGAATCGAA
CATACTGCGGCAGATTGTCCAAACTCATCAAAAGCTTATCCAGTTTCAAA
ATGAGGTCAAGGACTTCTTATCGGAAATCATTATTCCCATCTTCTTCGTC
AACTACACCGGTATATCTTTGAGCATGTACCAGCTGGTCGTCAACCCATC
CGCCATGTCCACTCTCCGCTTCGTCCGCTTCTTGTGCGAGTTCTTGTCGC
TGATCGCCCAGTACTATCTCTTTACTGACAGCTCGGAGACGGCAGACAAC
TGCAACCAAATCCTTCTCCGTTCAATCGAACAAAGCAATTGGTACAACTG
TTCTTCGTTTATAAGACGCGATCTCTGTGTGATTATGCGGCGCGCGCAGC
AATGCAACAAGCTGAGTTTCTACCAAGGGACTCTCGTGCTCAGCCGTGTT
CAATTTCTCAAGGTTCTTAAAATTGGATACTCTTTCGCCAACACCATGCG
GATGAAATCT
>OR25
ATGTTCGGTTTCATCGTCGCCAACAACATCATGTTCTGCCTCATCATCCA
TCAGATAGCGTTTAGCCCGATGCATCTGTCTCCAACTTTACACTTCAAGT
TATTGACGCAGCTATTGGGAGCCACAATCGAGTATTTCCTTGTCTGCAAC
TGTTCTGAGATCCTGGACGAATGCAATGCTATCCTACGCCGAGCGATATT

GAACTGTAGGTGGTACAGCTGTTGTGCTAGCACGAGGAGAGATCTCTGTG
TGATCCTGATCAGAGTGCAGAGGCCCAACTACTTGAAATTCTACAATGGT
TCCATCATTCTAAGCAGGGTTTTCTTTGTGGGCATCTTGCGGGTGACATA
CACATTTGTGAACTTCATGAGGCTCAGAAGTTTGAGCAAATCTTGA

>IR1
ATGCAGCTGGCAAGATCTTCATCAGCCCTGTTTGTTTACATCTTTACCCT
GTCAGTGTTCTATGGTAATCGAGTGTCACATGGTCAGATTGTTCAGGTCC
TTAATATTCTGTACATCAACAGCGACGGCAACACTGTGGCGGACAGGGCA
GTGGATGTGGCTCTCAACTTTGTGCGGCGCAACCCCAGATTCGGCATCCA
CATTGATGGCTTCTACAAGGTAGTTGCCATGGGGACGGACCCGCAGGAGT
TCCTAGACACATTGTGTGCAAAATTCAACGAGACAATCGCAGCAGGGAAG
CCTCCGGACCTGGTGTTGGACACCACGACCAGTGGCGTCATGTCAGAGGC
AGTCAAAACTTTCACGTCAGCCCTGGCACTCCCCACAGTGAGCGGATCTT
ATGGCCAAGATGGGGATATACGTCAGTGGCGTAATCTAGACGGCGAACAG
ATGAAGTATCTGATCCAGGTGAATCCCCCAGGAGATATCTTGCCTGAAGT
CATACGTGCCATCATCCTCATGCAGAACATCACTGACGCAGGCATACTGT
TTGATGACTCCTTCATTTTCGACCACAAGTACAAGTCCCTCCTCCAGAAT
ATCCCCACTCGTCACATCATTGCGCCCGTGGAAGACGCCCGCAGTGTCAA
GAGACAACTCTTCCGCTTCAAAGATCTGGACATTGTCAATTATTTTGTGC
TGGGGAGACTGCAAACCATCAAGATGATTCTTGACAGCGCCAACATCAAC
AAGTTTTTCGGGAGAAAGTTTGCTTGGCATGCGATCACGCTGGACAAAGG
AGCCCTCAAGTGTGCCTGTTCCAACGCCAGCATCCTGTTTGTGAAGCCAG
AGCCCGACCCCACCGCCAAGGAGAAGCTTTCCAATTTGAAGACTACGTAC
GGGCTTACAGCGGAGCCCGAGATAAGTGCCGCCTTCTATTTTGATCTGGC
GTTCCGGACATTCATCGCCGCCAAGGCCATGTTAGATGCGGGAGAGTGGT
CAAAAAACTTCACCTATGTGACTTGTGAGGAATACAATGAGGACAATCTT
CCTTCGAGGAAAAACTTTGACCTGAGGAAATATTTGAAGCAAGTATCCGA
GCCAACATCGTACGGGACATTCGTGATTGAAACCAACGGGCATAGCTTCG
AGGAGTTCACGGCTCATCTGGAGAAGGTGTCGATCATCAACAGTCAACCG
GTGAGCTCGGAAGCGGTGGGCACCTGGAAGGCGAACCTCAACTCCCCCAT
CATGGTCAAGGATGCAGCAAGTCTCGCCAACTTCTCGGCGGTCACCGTGT
ACAAGATTGTCACTGTGCTGCAAGAGCCGTTCGTGATGATAGATGAGAAA
GAGGGAGGAGGTATTGGCTACAAGGGCTACTGCATAGATCTCATCAATGA
GATCAGAAAGCTAGTGGGATTTGAGTACGAGATCTTTGTGGCTCCTGATA
ACTCGTTTGGCAACATGGACGAGAATGGACAATGGAACGGCATGATCAAG
GAACTCATTGAAAAAAGAGCGGACATAGCTCTGGGCTCCATGTCGGTGAT
GGCAGAGAGGGAGAATGTGGTGGACTTCACGGTACCCTACTACGATCTGG
TGGGAATCACCATCCTCATGAAGAAGCCCAAAACACCTACTTCCCTCTTC
AAGTTCCTCACGGTACTGGAAAGCGAGGTGTGGCTGTGTATCTTGGCCGC
ATACTTCTTTACAAGCGTTCTCATGTGGTTATTTGACCGGTGGAGTCCAT
ACAGCTACCAGAATAACCGGGAGAAATACAGAGACGACGAAGAAAAGCGA
GAGTTCAACCTGAAAGAATGTCTTTGGTTCTGTATGACTTCACTCACTCC

TCAAGGCGGTGGGGAGGCACCCAAGAATCTGTCCGGCCGTCTTGTCGCTG
CAACTTGGTGGCTGTTTGGTTTCATCATCATCGCCTCCTACACCGCTAAT
CTAGCCGCATTCCTCACTGTCTCCCGTCTCGACACCCCCATAGAATCGTT
AGACGACCTGTCCAAGCAGTACAAGATCCAGTATGCCCCTCTCAACGGCT
CTGCGGCCATGACATACTTCCAGCGCATGGCGGACATTGAGGGCAGGTTC
TATGAGATCTGGAAGGACATGAGTTTGAACGATAGTCTGAGTGATGTGGA
GAGAGCCAAGCTGGCTGTGTGGGACTACCCGGTGAGCGACAAGTACACCA
AGATGTGGCAGGCTATGAAGGAAGCGAAGCTGCCCAACACTCTAGAGGAG
GCGGTCCAGAGAGTGAGAGACTCCAAGTCCTCCAGTGAGGGCTTTGGATA
CTTGGGAGACGCTACGGACATTCGATACTTGGTGATGACCAACTGTGATT
TACAAATGGTAGGAGATGAGTTCTCCAGGAAGCCATACGCCATTGCGGTA
CAACAGGGATCACCTTTGAAAGATCAGTTCAATAATGCCATTTTACTCCT
CCTGAACAAGAGGAAGCTGGAAAAGCTCAAGGAGATCTGGTGGAATCAAA
ACCCGGAGAAGAAGAAGTGTGATAAGCAGGATAACCAGACAGATGGCATC
AGTATCCACAACATAGGCGGAGTATTCATCGTGATATTCGTGGGCATTGC
TCTGGCTTGCATCACTCTCGCTTTCGAGTACTGGTGGTATAAGTACAAGA
ATCCGCAGCTCAACGAGTTCGTGGCCAATAATCAGAACATAGATGAGATG
AACAGGAAGATGCAGATCTCGACCATAGAGTATGATAATGTGAGGAAGCG
ACACTACCAGGGGAACTATACCAAAAAATCAAGTTTGCCACCCGTCAGTG
ATCAATGGCTTCAGTAA

>IR2
ATGCAAGTACCCGTCGCTTCCAGCTCCAGAACATTGCAGTTCACCATGTC
CACACATCCACTCGGTCTCTCCCTGCTTCCTCTCCTGGGTCTCCTGCAAC
CACACCCCTGTACCTCAATGTCCCTCAACATCTCCAACCTCACCGAAAGC
CCGCAACCCTTCCACATCTCCAGCTACCAATTCTTCACGACCATCAAACG
CCTCAAGTTCAAATCTCCCGAGCTGGCCAAAGTTTTCAACGTGGCCATGT
TCAAGGTGACGCTCAGTCTCGACCAGTACCTGGCCATCCGAGATATCCTG
GGCCAGGTGTATCGGGTCAATGACCTTCAGGTGCCCTTGGAAACGCCGTA
CAATATTATCTTCATCAACTCGAAGAAAAGCATCAACCAGCTGTTGGACG
AGCCGAATGGTCTCTGGTGGAATTTGAATACCTACTACTACTTTGTCCTA
CCTAAACTCTCCAGGAATTTGCACCTCATCTTTGAGCGCCTGTGGATAGA
GCACTCCATCCTATATGTGTTCATCACCACCATCACTAACATGGAGAAGA
TCTTCGTATTCAACCCGTTTCTACCTAACAGCATGCGCCAGATTATGTCA
TTCAACGTCCGGAACCACAAAATAATCAACCGGTTTATTCGGAACCGCAT
TAAGAATCTCTACGGTTATCCGATGAATGTTCTCATGTTTGCCGTAAAGC
AGAAAGCAATCAAAATGAGCGATGGATCGTATACCGGACCGGATGGCCAT
CTACTCAAGTTAATATCGCAGACCATGAACTTTACCCCCATCGTCAGGGA
ACCTGCCGATGGTGGTCGTTACGGATGGCGAAACTCTGACGGCAATTTCA
CCGGACTACTAGGTGAGCTCATACACAACCGTGCTGAGATGGGCTTCAAC
AGCATCTTCATTAAGAACTACCACACGACAGATATAACTTTTTCAACAGC
CTTCGATGTGGACGATGTGTGTCTGGTCGTTCCAAAGTCCAAGTTGAAGC
CAGAGTGGCAAGGGATTTTCCTCGCGTTTGATGAGACGACCTGGGTGCTC

ATCATCCTTACTTACCTCTTCTGCGTCGTTGTCTGGTACTGGGTCAAATC
CGTCATTTACGAATCCTTTGACCATGCTTTGCTTGTTCTAAACGTGTTCC
AAGCATTTATCCTTGCCCCTCTTCACCAAGTGCCCAAGTTCTACTCCGAG
AAAATGATCTTCCTGTCCATTCTTCTCTTCACTCTGGTTATCTCCAACAG
TTTTCAAGGCTCTTTGGTGCGTCTGCTCACTCACCCAACATACGAACCAG
ACATCAACTCCCTCGCTGACTTGGACGAATCAGGACTGCCCATCCGATCC
ACCGCCAACAATCTGCGTGAAACGTTCAGGACCTATGACAATAGGAACGT
GATTTACCAGCACCTGTGGAACAAGTTCGTCTTCACTCCGGACAAGAGCG
AGAACATACTGACTCGGGTATCTTACAAACGGGATGTCGCAGGGATTGTG
AGGAAGAACATAGCCAAAGAGGCGATGAAGAATTACATCGAAGATGACAA
GGTGTTGCTTCACATTGTCCCCGAGTGTCCTATCTCCTACAATCTCGCCC
TCATGTTCAAGAAGCAGAGTCTCTACATTCAAGTGATCGACGACATCATC
TTGCTACTGGTTCAATCCGGAATACTCAACTCCTATCACATGACCAACAC
GACCAGTGTCATTCGCCTGTACAATCAACGCAAGAATCATCAGATCTTCA
CCATCAATAATGTCTACATCGCGTTCATTATTCTCATGATTGGATACGGG
ATAGCGCTGATTGTCTTTGTGTTTGAGTATTGCCTGGATCACATCTCTAA
CCAGAAGTATCGGACTTGGTGTGCGGAGTTGGAAACGTCGGAAAAGCCAA
TAAATAAACCTGAACATGCCGTCAAGCCCGATTCACAGAAAGAGGTTGCA
CAGGTCAAAATTGAAAAGCCAAACGAGGAAGGTGTATATTTACCGTTTCT
TTTGTAG
>IR3
ATGTCAACTTTTACTTGCTGTTCTATTTTTTCTGTGTCAATTGTCTTGCT
AGTTGTCATACTTGTGCAATCAGAAAGTCGTACACCACAGATAAGTGCAC
ACAATAGTATTGCAGGCAAAGTTCCTGAAACACAAGTAATTAAGATTACC
CAAAGCATTGACAGGAGTATAAAACCTGATGTTGAAATCGAAATCAATGA
TGAACCCAGATCGAAAAGAAATGTGCGCGAAACTCAAGGGTCAGGGGTTA
ATCTGAATTTAACCGTGACCACAGACAATGTCACTAAAGATCTTGCTTTC
AAACTGCCTGCTAACAACGAATCTGAAGCCTCAAATATTGAGATCAAACT
GGGCCTAAAAGAAAATGGTAGAGTGCAAAATCTTAGTGGAATTTTAACCA
ATATTGTACGCAATAGTGGTACGGTTGTAAACTCTAGTATGGTACAAGGG
CTGCCGATTTCAATATACCACAATATTAGTTCCCAAATTCAAGCCAGATC
GAATGTCAGTCATAGACCCGACATAAAGCATAGAGCTGGCGGTGAACATA
ATAGCGACTCAAAGATCGAGAAGTTCACTATTCAAGATACTTTTGATATT
TTGAAATATTATGACACCAAAAATGATCAAGACTCGGATATTTCTAAACT
GATCAAGTATGTTATGAAACATTACTTGTCCAACTGTATGATAATCTTTC
TCTATGATGAACTATTCTATGCCAGCAATTCTCTCAGGGGTGTCATCACA
GATATACTTGAGTACTCGCCCCTACCCATAATGCACGGTATGATCAACAC
CTCCGGCCTCTTTTACCACACCTCCGCGCACCTTCTGGACCCTCCGGAGC
GGAAGTGTCGCAACTTTATCATTCTCACAAAGAGCTTAGAGAACAGCGCA
GACCTACTGGGTCGACAGCGCGACAGTAAGATCGTGTTCGTAGCTTTGAT
GTCCTCCTATCAGATCAAGAGCTTTCTCGTAGATCCATTGTCGCACAAAA
TTGTCAACTTGCTCATAGTAGCCGATCCCAACCTGCAGCATGACGAGTTT
AATCAGAAATCAGAATTCTACACCATTGACCTGTACACTCATCGTCTCCA
CACGAACAGCCTAGGTACTAGTGTTCCTTGGATCCTGACATCCTGGAGGA

ATGGCAATCTCACTCGTCCCTCGGCCAATCTCTTTCCTGACAAACTCAAC
TCGGGATTCCAAGGACATCGCTTCATCGTATCGGTAGCAAAGCAACCACC
GTTCGTCTTCCGCAGACAGACATTTGAGCATGACAATTCAGGCAAGATAA
TTCAAACCGAGACAGAACAATGGGATGGACTGGAGGTGAAAATCCTAAAG
CAAATGTCCGATTATCTCAATATTACACTAGACCTGCGTGAGACCTCTAG
TTTGAATCCTGGTGACGGAGAATCAGGCAGGGTTATCTACGACATCATGC
AAAACAAAGCTGTTCTGGGTCTGGCAGGAATATCAATGACCAATGAGCGA
CTGCATAACGTGTCCTTCTCAACCAGTCACTCCCAGGACTGTGCTGTGTT
CATCACTCAAACCTCTCTGGCACTCTCCAAGTATCGGGCTATTTTCGGTC
CATTCAGATGGGAGGTGTGGGTGTGTCTCGTGCTGGTCTACTTACTTGCT
ATCATACCCCTATCATACTCGGACAAGCTAACCCTCAGACATCTCTGGGA
GAACCCGGCGGAGATAGAGAACATGTTCTGGTATATGTTTGGCACATTCA
CCAATCTGTTCACGTTCCGGAATGTCAACTCATGGACCTCTAGCAAGAAA
GCGTCAACACGAGCTCTCGTAGGTACATACTGGGTGTTCTCAATCATAAT
CACGGCAGCATACACAGGGTCGATCATCGCTTTCATAACAATGCCTGTGT
TTCCCGATACAATAGACACGCTGCAACAGTTGAAGAGCGAGAGATATCGC
ATCGTAACATTGGACAGTGGCGGATGGCAAACGTTCAACGACACGGAGAA
GAAGACTTTGGAGGACGCTGTGTTTGAGCACTGGGAGCTTGTGTCATCAG
TGGAGGATGGCCTCAAGAATGTGACTGAGGGACATTTGCTGTGGAACTAT
GCCTTCCTCGGATCCAGGGCACAGCTAGAGCATATTATCAAAAATAACTT
CGAATACAACGTGAGAAAGCGGCGTCGTCGTAAGAGAAAGAAGTTCCACA
TCAGCAGGCAATGCTATCAGCGCATGTACGTCGGCATCGTCTACTCCAAG
ACCTCTCTTCTCCATAATCAACTCGATAGCTTCATATTGAAAGCTCAGCA
GTCAGGTCTAATGAACAAGTGGATGAGGGAGGTGCAGTGGGACAACTGGA
GGAATGAGAGAGGTGGCAGACTTCAAGCAGGAAACAGAGATCTCAGGATA
ACAATCCCAGATGACCGCATGTTAACCCTTGATGACACGCTGGGTATGTT
TCTCCTACTAGCATTTGGTTACACCCTTGCGATTATCGCCTTCATCAACG
AATGTCTCACCCACTACAACAAGCGGATCTTGTGCTGCAAGAAGAGAGAT
CCTCACACTCACCCCCCCACTATCACTCTCACTGATAACACGCCAGTCAC
ATCTGTGGAGAATCTACAGGAGGAGTCAGAAGTCCAAGCCTATGATCTGA
GCAGTCCAGAGCATAAGCTACTCATCGTGAGGAGTATTAGTCCTCATCGG
AGATACTCCATGACGCAGGTTGATGATAGAATCCTGGAGATTTGGGCTAA
GAGACGACTAAGTTGTTGA

>IR4
ATGTCAACTTTTACTTGCTGTTCTATTTTTTCTGTGTCAATTGTCTTGCT
AGTTGTCATACTTGTGCAATCAGAAAGTCGTACACCACAGATAAGTGCAC
ACAATAGTATTGCAGGCAAAGTTCCTGAAACACAAGTAATTAAGATTACC
CAAAGCATTGACAGGAGTATAAAACCTGATGTTGAAATCGAAATCAATGA
TGAACCCAGATCGAAAAGAAATGTGCGCGAAACTCAAGGGTCAGGGGTTA
ATCTGAATTTAACCGTGACCACAGACAATGTCACTAAAGATCTTGCTTTC
AAACTGCCTGCTAACAACGAATCTGAAGCCTCAAATATTGAGATCAAACT
GGGCCTAAAAGAAAATGGTAGAGTGCAAAATCTTAGTGGAATTTTAACCA

ATATTGTACGCAATAGTGGTACGGTTGTAAACTCTAGTATGGTACAAGGG
CTGCCGATTTCAATATACCACAATATTAGTTCCCAAATTCAAGCCAGATC
GAATGTCAGTCATAGACCCGACATAAAGCATAGAGCTGGCGGTGAACATA
ATAGCGACTCAAAGATCGAGAAGTTCACTATTCAAGATACTTTTGATATT
TTGAAATATTATGACACCAAAAATGATCAAGACTCGGATATTTCTAAACT
GATCAAGTATGTTATGAAACATTACTTGTCCAACTGTATGATAATCTTTC
TCTATGATGAACTATTCTATGCCAGCAATTCTCTCAGGGGTGTCATCACA
GATATACTTGAGTACTCGCCCCTACCCATAATGCACGGTATGATCAACAC
CTCCGGCCTCTTTTACCACACCTCCGCGCACCTTCTGGACCCTCCGGAGC
GGAAGTGTCGCAACTTTATCATTCTCACAAAGAGCTTAGAGAACAGCGCA
GACCTACTGGGTCGACAGCGCGACAGTAAGATCGTGTTCGTAGCTTTGAT
GTCCTCCTATCAGATCAAGAGCTTTCTCGTAGATCCATTGTCGCACAAAA
TTGTCAACTTGCTCATAGTAGCCGATCCCAACCTGCAGCATGACGAGTTT
AATCAGAAATCAGAATTCTACACCATTGACCTGTACACTCATCGTCTCCA
CACGAACAGCCTAGGTACTAGTGTTCCTTGGATCCTGACATCCTGGAGGA
ATGGCAATCTCACTCGTCCCTCGGCCAATCTCTTTCCTGACAAACTCAAC
TCGGGATTCCAAGGACATCGCTTCATCGTATCGGTAGCAAAGCAACCACC
GTTCGTCTTCCGCAGACAGACATTTGAGCATGACAATTCAGGCAAGATAA
TTCAAACCGAGACAGAACAATGGGATGGACTGGAGGTGAAAATCCTAAAG
CAAATGTCCGATTATCTCAATATTACACTAGACCTGCGTGAGACCTCTAG
TTTGAATCCTGGTGACGGAGAATCAGGCAGGGTTATCTACGACATCATGC
AAAACAAAGCTGTTCTGGGTCTGGCAGGAATATCAATGACCAATGAGCGA
CTGCATAACGTGTCCTTCTCAACCAGTCACTCCCAGGACTGTGCTGTGTT
CATCACTCAAACCTCTCTGGCACTCTCCAAGTATCGGGCTATTTTCGGTC
CATTCAGATGGGAGGTGTGGGTGTGTCTCGTGCTGGTCTACTTACTTGCT
ATCATACCCCTATCATACTCGGACAAGCTAACCCTCAGACATCTCTGGGA
GAACCCGGCGGAGATAGAGAACATGTTCTGGTATATGTTTGGCACATTCA
CCAATCTGTTCACGTTCCGGAATGTCAACTCATGGACCTCTAGCAAGAAA
GCGTCAACACGAGCTCTCGTAGGTACATACTGGGTGTTCTCAATCATAAT
CACGGCAGCATACACAGGGTCGATCATCGCTTTCATAACAATGCCTGTGT
TTCCCGATACAATAGACACGCTGCAACAGTTGAAGAGCGAGAGATATCGC
ATCGTAACATTGGACAGTGGCGGATGGCAAACGTTCAACGACACGGAGAA
GAAGACTTTGGAGGACGCTGTGTTTGAGCACTGGGAGCTTGTGTCATCAG
TGGAGGATGGCCTCAAGAATGTGACTGAGGGACATTTGCTGTGGAACTAT
GCCTTCCTCGGATCCAGGGCACAGCTAGAGCATATTATCAAAAATAACTT
CGAATACAACGTGAGAAAGCGGCGTCGTCGTAAGAGAAAGAAGTTCCACA
TCAGCAGGCAATGCTATCAGCGCATGTACGTCGGCATCGTCTACTCCAAG
ACCTCTCTTCTCCATAATCAACTCGATAGCTTCATATTGAAAGCTCAGCA
GTCAGGTCTAATGAACAAGTGGATGAGGGAGGTGCAGTGGGACAACTGGA
GGAATGAGAGAGGTGGCAGACTTCAAGCAGGAAACAGAGATCTCAGGATA
ACAATCCCAGATGACCGCATGTTAACCCTTGATGACACGCTGGGTATGTT
TCTCCTACTAGCATTTGGTTACACCCTTGCGATTATCGCCTTCATCAACG
AATGTCTCACCCACTACAACAAGCGGATCTTGTGCTGCAAGAAGAGAGAT
CCTCACACTCACCCCCCCACTATCACTCTCACTGATAACACGCCAGTCAC

ATCTGTGGAGAATCTACAGGAGGAGTCAGAAGTCCAAGCCTATGATCTGA
GCAGTCCAGAGCATAAGCTACTCATCGTGAGGAGTATTAGTCCTCATCGG
AGATACTCCATGACGCAGGTTGATGATAGAATCCTGGAGATTTGGGCTAA
GAGACGACTAAGTTGTTGA

>IR5
ATGCTCTCCTCAGGAGTGGTCTCGGTGCTCATTCTCTGCAGTGCCTTACT
AAAGGGAGTGATCTGTGTTCCCAAGGTCATCAAGATAGGGGGGCTGTTCG
ACTCGTCGGAAACCGACCGCGTCAATGAAAAGGTTTTCCGCCGGGCCATT
GATAAGGTGAATGAGCTAGATCTACTGGAGGGTACCAGACTGATGGGTCT
AACTATGCGGGTACCTGAACATCAGAGCTTCCAAGTTCAGAGGAGTGTGT
GCGAACTAATTCAGCAAGGTTGCGTCGCCATCTTCGGTCCCCGCTCGCCG
GACACGACCCCAATCGTCGAGTCGGTGTGCGACGCCAAAGAGATTCCGCA
CATCGAGGCCCGATGGAACCACAAGAACGCCCGCGGCTCCTGCTCCGTCA
ACCTGTATCCGGACGCCGGCGTTCTGGCCGCCAGTCTAGTGGACGTGGTG
CGTGCGCATTCGTGGACTCACTTCACCCTCGTGTATTACGACAACGACGG
ACTGTACCGGGTCAAGCGACTGCTCGAGATGTACGACCGCAAGGGACACA
CAATCGTGTTGAGAAAACTGAAAGACGATGGCACAGGCAACTTTCGACCT
CTGTTGCGTCGAATCAAAAACTCGGAGCAGACATTTATCCTGATCGACGC
CCCCACGGATATTCTCTTCGAAGTGTTGAAGCAGGCGCAACAGATTGGTC
TCATGAGCTCCGAGCAGGACTTCATCATTAACAATTTGGACATGCACACC
ATCGATATGGAGCCGTTCAAATATAGCAGCACCAACATTTCAGGAATTCG
AATGCTCAACACCTCGTCCGAGCGACTGAAGAAGACCATCGAAGAGTGGG
GGGATGCCGAGTTGGACATTACCCCCGACAACATCAAACTGGAGACCGTG
CTCATCTACGACGGAGTCTTCCTATTGGCGCAGTCTCTACTCAACGCACC
CATCCAATATGATCCCACCAGTATTCGAGTGACCAGGGAAGATGGTAAAG
ATGGCGGTGGCAGTAATCCTCCCGAGGACGACGGCAATCCTACGAGTGAG
GACGGCAACACCGAACAAAACGGCGAAAACGCCCGCAAAGAAGATCCATC
CGAGCATGCCGCCAGCGGAGTGGTCGAGGCAGTCCGGTGTGACAACGGCG
TTGCGTGGCCGCACGGCTACAGTATCATCAACGACATCAAGTCCGGCCAG
CTCAAGTCGGCCCTGTCTGGCAACATCCGGTTTGATCATCAAGGCTTCCG
GACGAGCTTTCCGCTGGAGCTGACCAAACTGACCGAGGATGGCGTTGTGG
GGACAGGCAAATGGTCACCCCGGGAGCGCGTCAAGTTTTACAACGAGAAA
CCGGCCGGTGACTTTCGCAACACAACATTCCGAGTGGTGATTGCCCTCAC
ACCGCCCTACGGTATGGAACGCGAGTCACCAAAACCGCTCACCGGTAACG
ACCGGTACGAAGGGTTTGCGGTGGATCTGATTCATGAGCTCAGCATCCTG
TGCGGATTCAACTATACTCTGGAAGTGCAGGCTGACAGCAAAGGAGGCAG
CTACGACAACAACACCAAGAAGTGGAATGGTATGATCGGCAAGATTCTGT
ACGGGGAGGCGGATCTAGCTATCACATATTTGACTATCACGAGGGAGAGG
GAAAATGTGGTGGACTTCACCATGCCCTTCATGAATCTGGGTAAGAACTT
GTGGTATGATCGGCAAGATCCTGTAGGGAGAGGCGGATCTCCTTCATGA

>IR6
ATGAAATTTTTGTCTTCAAGTGACGCCCAAATTTTGAGCTCGTTCACAAT

GGCCACACCAGTGTTTACCTCCTTGATTCTGCTGCTGTTCAGCATTCCTG
ACTCACTGCAGCAGCAGCACAACCCGCATTTGTTCAACATTGGAGGAGTT
CTTAGCAATAATGTGAGCGTGTCAAATTTCAAGGAAATAATCGATCACAT
TAACTTTGACATTCAGTATGTCAACAAAGGTGTCACATTTTCGGCCACAG
GCATTCAGATGGATCCGAACCCCATCCTCACTGCTTTGAGCGTGTGCAAG
TTTCTAATCTCAGAGCGAGTGTACGCTGTGATAGTATCTCATCCTCTCAC
AGGAGATTTATCTCCAGCCGCCGTTTCCTACACCAGCGGCTTCTATCATA
TTCCTGTCATAGGTATTTCATCTCGAGATTCTGCGTTCTCGGATAAGAAT
ATCCATGTTTCATTTTTGCGCACAGTTCCTCCCTATTCACATCAAGCAGA
TGTTTGGGTCGAGCTGCTCAAACATTTCAATTACATGAAGGTGATCTTCA
TCCACAGCTCTGATACAGATGGGAGAGCGTTGCTGGGGCGGTTTCAAACC
ACTTCTCAAAATCAAGAAGACGACGTTGAAATCAAAGTTCAAGTGGAAGC
TGTGATAGAGTTTGAGCCAGGGCTCACCACCTTTAAGGATCAGTTGTACG
AGATGAAGAATGCTCAAGCACGGGTGTATCTTATGTATGCAAGCAAACAA
GATGCAGAGATAATCTTCAAAGATGCCCAATATTTGAACATGACCGAAAC
TGGCTACGTTTGGATTGTCACCGAGCAAGCTTTGACAGCTAAGAATGTTC
CCGCAGGAATCATAGGACTCAAGCTGGTCAATGCTACGAATGAAGATGCG
CACATAAGGGATAGCATTTATGTCCTGACTTCTGCATTGAAAGAAATGAA
CCAGACAGCTGTGATCACAGTAGCTCCACAGGACTGCGACAATTCTGGGT
CAATCTGGGAAACTGGAAAGGTCCTGTTCGAATTCATTCGTAAGCAGGTT
ATCATGAATGGGTACACCGGTAAAGTTGCGTTTGATGACAATGGTGATCG
TATCAATGCTGAGTACGATGTGGTAAACATCCAGAAACCAAGTCCCTACA
ATAAAGTACATCAAAAATCCGTCGGCAAATACTATTACAGTAATGTTTTC
TCCCGAATGAAACTGAAGGTGAACGAGTCCAACATCGTATGGCCGGGAAA
TTCCAAAAGTAAGCCGGAGGGTTTCATGATTCCCACTCACCTCAAGGTAC
TCACCATCGACGAGAAACCGTTTGTCTACGTCCGTGCTTTAAGTCAAGAG
GGAGACTCTTGCAATGCAGATGAGATCCCGTGCCCTCATTTCAACTCAAC
AGACTCGATGGAAGGTAAAATGCAGTGCTGCAAAGGTTTCTGCATGGATT
TACTTCGCGAGCTCTCCCGTACCATAAACTTCACGTACAGCTTAGCTCTC
TCCCCTGACGGACAGTTTGGCAGTTACATTATCAAAAATACCTCAGGAGG
TGGAGGTAAAAAGGAATGGACGGGTCTCATAGGAGAACTAGTTTCCGAGA
GAGCAGACATGATTGTAGCGCCTTTGACTATCAATCCTGAACGAGCAGAA
TTTATTGAGTTCAGCAAACCTTTCAAGTATCAAGGAATAACCATTTTAGA
AAAAAAGCCATCGCGCTCTTCCACCCTGGTATCATTCTTGCAACCATTCA
GCAACACATTGTGGATTCTGGTCATGGTGTCAGTCCACGTTGTAGCACTG
GTCCTGTACCTCCTGGATCGATTCTCGCCGTTCGGAAGATTTCGGCTTTC
CAACTCAGACAATACAGAGGAGGATGCGCTGAATCTTTCCTCTGCCATCT
GGTTTGCATGGGGAGTTCTTCTCAACAGTGGCATTGGTGAAGGTACCCCT
CGAAGTTTTTCTGCGCGCGTCCTGGGAATGGTATGGGCAGGATTCGCCAT
GATCATTGTGGCATCATACACTGCTAACTTGGCAGCTTTCCTCGTCTTGG
AGAGACCTAAAACCAAACTAACAGGAATTAATGACGCTAGGTTGAGGAAC
ACCATGGAAAATTTGACGTGTGCCACTGTGAAAGGGTCTGCGGTCGACAT
GTACTTCAGACGTCAAGTGGAGCTGAGCAATATGTACAGAACCATGGAGG
CTAACAATTACGATACAGCTGAGGATGCTATCGCGGATGTGAAAATTGGA

AAACTGATGGCATTTATCTGGGACAGCTCCCGCTTGGATTTTGAAGCGGC
GCAGGACTGTGAGTTGGTGACGGCCGGAGAACTATTTGGACGGTCAGGAT
ACGCTATAGGACTGCAGAAGGGATCTCCCTGGGCGGATGCTGTGACTCTC
GCGATACTCGACTTTCACGAGAGCGGCTTCATGGAGCGTCTGGACAACAC
ATGGATTCTGCAAGGGAATGTGCAACAGTGTGAACAGTACGAGAAAACAC
CCAACACCCTGGGACTGAAGAACATGGCGGGGGTGTTCATTCTCGTAGCA
GCGGGTATAGTTGGCGGCATAGGACTCATCATTATCGAGATGGTGTATAA
GAAACACCAGATTAAGAAGCAGAAACGCTTGGAGCTGGCAAGGCATGCGG
CGGACAAGTGGCGAAGTGCCATTGAGAAGCGCAAAAAGCTACGATCTCGC
GCCGCTGTACAGAGGAGGCTCAAGGCAAATGGCATCAACGATACCGTGAT
GCCCATCAGCTTATCAGTGGAGGAGCTACCGCGGGGGCTGGAGCTACAGA
GCCCCGTTCATTCCATGGAACCCTCCTTTCCGCGAGAGATGTCGCCACTG
AGAACCCCCATCAGATGTTGA

>IR7
ATGTTTCTACCATGTATTTTGTGCCTGCTGTGTTTCGGGCGAAGGTCTTT
GGGCGTTCTGCACTTGGACCCCTTGCAACACCAAGAGCCGGTGCCCCGGG
TTGTCGATCTGGTGTTAAGTAACAGAAGCCAAAGTCAGAATGAGACGCCG
GTGGCGGGTGGCACCTGGACACAGGCGAAGTCTCTGAAAGCGCTGTTCAG
GGCTAGAGTGTCCACAACTTCCGAAATGGAAGGGGAAGAAGAGACTGAGT
TGTCATCTGAACCGGTGAGTGACGGTCAGGTCGATGTGATGAAGAATATT
ACGGACAAGGTTGCCAATGTTCTCATTGATGTTGTTCTGGGACTTCACGA
GCCCAGGATGGCTCTGATCAGCGATGATATTAACGGAAACGTCTCAAAAA
ACAATCTTCTCCGAGATTTGCAAAACGGCGGGCTGTCCTTGTCACATTAC
TTCTTTAATCGCACACAGGATATGAAATACCTTCTGAAAGATTTTGAGCG
ATGGCAAAACAAGCTAAGGAAACTAGTGTATGTGATAAATGTACGGAAAT
ACCTCATGGAAGAGATTCTGCTGAAAATCCAAAAGAAAGACAAAATGATT
CGACGTCACATCTTCTACATTTTCATCTCTCCGGAGCCTCAGCTTAGCAG
ATTCTTCCAGCAGAACATTCTGGAGGCCATGAAGATTATAGTCATTGCCA
AGTCCAGGCCTGGAGTTTATCAAATCCTCTACAACCAAGCGTCTTCAAAC
AGCAGGGGATCTCTGGAGCGAGTTAACTGGTGGTCCCTGACCCCCATCAA
AAAGGGCCTCTTCCAGTACCCTCTCCTTCCACCCGCCATCAGAGTCTACT
CCAACTTTCACGGCCGAAATCTACTCATTCCGATTTTACATAAGCCACCC
TGGAACTTTGTGCACTATGAAAATGACACCATTCAGCACATAGAGAGTGG
CAGGGATGACAAGCTACTGTCCTTGCTAGCGTCCAAACTTAATTTCAGGT
ATAGCTTTGTGGACCCACCAGATAGAAACCAAGGATCTTTCATTAATGGC
AGCTATACTGGAGCTCTGGGCCTTGTGACGTCACGGGAGGCAGATATGTT
CATGGGCGACCTCACACTGACCCACGAGAGGAACGAGGCGGTGGAGTTTA
CGTTCTTCACACTGGCCGACAGTGAAGCGTTCCTCACACACGCGCCAGAA
ACTCTAAATGAAGCTCTGGCATTGATCCGGCCTTTCCATTGGAAAGTTTG
GCCTCCCCTGATTCTATCTGCAGTGGTTGCAGGGCCGGTCCTCTACCTGA
TCATTCATTTCCAACTCAAGTGGCGTACGGTCAACGTCTCCCGGTCCAAC
CTGGATCGGCTGTTCAGCGATTGCGTCTGGTTCATTCTGGTCATTCTGCT
CAAACAAAACGGCAAATTCCCAAGCTACACAAGCACTGGAAGAATGATGG

CACTGCTCCTGTCTTTGTCGGCCACATACGTCATTGTGGACCTCTACTCT
GCCAACTTAACCTCCATCCTCGCCCGGCCTCGCAAGGAGCAGCCTATTCG
AAACATGGAGCAACTGGTGGATGTAATGAAGTCCAAACAATATCATACAG
TAGTGGAGAAGAACAGTGCATCTCAGAGTATGCTGGAGGTGATGTATGGA
GGTGACATGGAGGTGATATGGAGAACGGCACGGGAGAGCTAG

>IR8
ATGAGGTGCAAATTATCCGTGTTCTTCTTAGTTGTCTGTCTCCTGGTTCC
AGTCTTCACTTTGAAATTGTTGATGGTGACAGAAGAAGAAGACGAATTGA
GCGATTTTTTTCTTAAACAAATCGTACATGACGAAAACGTTAGTGTAGAA
GTTATCAGTGTATTGCGATACAATGAAACAGAGGCAAGAAGAGAGTTTTG
TGAGCTGAGTGAAGTTTCAATCATCGTGGACGTCACCTACTCGGGATGGG
GAGTTATCAAGAAATTTTGTGAACTGTTCGGTATCCATTACATTCACTTT
GACTTGACTATTCAGCCCTATCTCAATATGGTGGTGGACTTTCTGGAGAA
TCTACGAAACAGCACCGATTCTACCTTCGTTTTTGATACTCTCATAGGCC
AGCGGCAGAGTCTACCTTACCTGCTTCGCAATTCCCGCATGAGGATCTCG
ACATTTCTAGGACTAACTAATGATGTCATCAAATCTTTAAGGACTAAAAA
CCCTTACCCCACTTTTATCATACTGGTGGGAGAACAGCAGATCCTCCAAC
AAGTATTTCTGCCAACGTCTCAACTAATGGAAGCTCTGCTCAGAAAGTAT
TCCATCGTGCTTGACAAAATGCTGAATGACCTCAATAAGTCCAACATGTT
ACATGCAAGAAAGTTCCAATGCACGGTGGAGGAAGATGGCTCCTTGACAG
TACCTGACACGGACGTTAAGGATAACAAACCTCTCAACCATCTCTATGTT
TTTCAGGCCCTCCAGAACAACCCTCCCCTGACTTTCAGTGAAAACACCTC
TGTCATTTCCTACAAGATAGATCTCAACATGTCCACTTGGATCGCTACCA
ACCGCAGTATTCATTTGTTTGGCACCTACAGCACTAGCAATGGCTTCAAC
ACAAGTCGTATGGTAGACAAAATCCCCCGCTATTTTAGGATAGGTTCTGT
TGTGTCTGTCCCCTGGACCATGCCCATCCTGGACCCTCTCACTGGCTACG
TAATGAGAGACTACCAGGACCGGGAGATGTATCGTGGCTACTGCATTGAC
CTGATCAATGAACTGGCCAAGCTGATGAACTTCCAATATGAGTTGGTCAT
CGTCAGAAAGTTTGGGAAGAAACTGGAAAATGGCACCTGGACTGGTTTGG
TTGGATTACTCAAGCAAGGGATACCGGCCCATAGTAAAATATCGAAACAG
AAAATAGACATTGCCGTGGCAGCCCTTACAATGACCGCAGAGAGGGAGGA
GGTGGTAGACTTTGTGACGCCCTACTTTGATCAAACTGGCATCACAATTG
TGATTCGCAAACCCATTCCCAAGACCTCCCTGTTCAAGTTTATGACCGTG
CTCAAGTCTGAGGTTTGGCTTTCCATCGTGGCTGCTCTGGTAGTTACCGG
TATCATGATATGGCTATTGGACACCTACTCACCTTACAGTGCGCAGAACA
ATCCACAACTCTATCCACCATCTACCAAAATCTTCACTCTCAAGGAGAGC
ATCTGGTTCGCCTTGACATCTTTCACCCCTCAAGGAGGAGGAGAAGCGCC
CAAAGCTCTAAGTGGGCGCACTCTGGTCGCATCCTATTGGCTGTTTGTGG
TTCTCATGCTGGCAACGTTTACTGCTAACCTAGCAGCATTCCTAACTGTG
GAGAGAATGCAGTCCCCAGTTCAATCCCTGGAACAATTAGCGCACCAGTC
CCGCATCAACTACACTGTAGTGGACAACTCTAACGCGCACGAGTACTTCA
AGAACATGAAACACGCTGAGGATATTATTTACAGAGTTTGGAAAGAAATT
ACATTGAATGCTTCGTATGATCAGAAGCAGTTTCGTGTGTGGGACTACCC

AGTCAAGGAGCGATACGGTCGAATACTAGAGGCTATCGAAAAAACGGGAC
CCGTGCAAGATGTCAAAACTGGTTTCGACAAGGTTCTGGAGAGTGAGCAG
GGAGAGTTTGCCCTCATTCATGATTCTGCCGAAATCAAGTACGAGGTGTC
CAAGAATTGCAACTTGACGGAGGTGGGAGAACTTTTTGCCGAGCAACCCT
ACTCCATAGCCGTTCAGCAAGGCAGTGAGCTCGCTATTGAAATAAGCTCT
GTTATTTTAGATTTACAGTCCGACAGATATTTTGAATTTCTAGACTCGAA
ATATTGGAACACGACTATGCAGAATAAACAATGCTCCAATGAGGACGAGT
CAGAGGGTATAACACTGGAAAGTTTGGGTGGTGTATTCATAGCAACTCTA
TTCGGGCTAGCCCTTGCCATGGTTACTCTAGTGCTGGAAATCTTCTATCA
CCAGAAAAATAGGGCCCGAAACAATCGTCACTTTGGAAAATTAAAACAAA
CTGCCGCTCCCATGCAGCGTCAATCATTTGAAATTCAAAAGTCTTTTTTT
CGCCCTTACAAACCAATTTTCGACTCCTTCAGACGCAAGAAAACCGTTCA
CTCTATATCAAATATAACAAATGTGCGCGAAAAAGATAATCTAAATATCC
TGAAAAGTCATTCTAAAATGGCAACCATTCAGGAGAAGGAGATGGATAGC
TTTCCGGACGCTTTCCTCTATGCTGGTGACTTTGGAGGGGTACATCGGAA
GCTGGAGATGAGGAAAAAGCCAAGGATGATCTCCGTGCTCCCATCCGACG
GCTGGTATTGA

>IR9
ATGTTCACTGCAGGATGGACATTAATCTTCCCTGCTGTATTCAGTTTGTT
GATTACGACAACTATGGCTGAAGACGTCCTCAAATATTCACATACGTCCA
ACGATACGATTGCTATTATAGTAGATCCGAGGTTCATGCCAGACAAAACC
AGCAAAGTCATTGCTAGCATCAAAGAGAAGGTGGATCAACTGAAACAAGA
AGAGCTGAAACAAGGGCTTCTCAATGTGGACTACTTCTGGACAACACGCG
CTATCATCAATGCAGAGTACACGGGTTTGGTGATTGTGGCGGCATGTGTA
GATGTGTGGAGAATCCACGCAGACAACGAAGAGAACTCGCTGTTCTATTT
GATCATCACGGAATCGGATTGTCCCCGACTCCCTGCTACGGAGGCCGTCA
CTCTGCCTCTGGGAGCGGCTGGCTCGGAGATATCGCAGATACTTTTGGAC
CTGCGGATCTCCCGTGCCTTTAACTGGAAAACGGTCAACCTTATTCATGA
CGATTCAGTGGATCGAGATCTGATGGGTGGATTCATCGGTTCTCTTACCA
AGGAGTTGCCCTCCAAGGAAAGTCTGCCCTCTTCAGACGTAGCCGTGTTC
AACTTCGTATCAACTGATGCTGATTGGGAGAAAACTAAACGAATTCACAA
GTTGCTTTCTTCAATCCGAATAAGAAAAGAAAATTCCAACTACATGGTGA
TTGTATCCCACCAGATGATTGGTACAGTGATCACAGTGGCCAAATCTCTG
GGTCTAGTGAACCCTATCACCCAGTGGCTCTTCCTCATCCCGGATACGAA
GCATAACTTCAACATTTCCATGTTCGGCAACTTGCTAGGAGAGGGAGAGA
ATGTAGCGTTTTCCTACAATTCCAGCACAGGAACTTCGCAATGCATTGGT
GGCCTCTTATGTCACGTGGAGGAAATCCTACGAGTCCTGCTGGTGGCCAT
AGACAAGTCCTCCATGGAGAAGTACGAGCTCTCCAACCAAGTGTCTGAAG
AGGAGTGGGAAGCCATAAAACCGTCCAAAGAGGAGAGGCGAAACTCCCTC
CTCATGTTCCTCAGAGCACGTTTAAACGAGGTGGGCAAGTGTGATATCTG
CACAATGTGGACGTTCAAATCAGGTGAGACGTGGGGGGATGAATTCCAGG
ACAACATGTCTCCTGTGATGGATCTGCTTGATGTCGGAATCTGGACACCT
CGTGACGGACTTCGGTTTAGCGACGTCCTGTTTCCTCACATTGAGCAGGG

CTTCCGGCGCAGAACCTTGCCGGTGATATCTTTCCATTTTCCACCCTGGC
AAATCATCACTTCGAACCCCGGAGAGCCTATCAAGTACAAGGGAGTTGTT
TTTGAAGTGCTAAACCAGTTGGCTAGAAATTTAAACTTTACGTACACCAT
TATCATAGCATCGAACAATACGGAGGGGTACACAAATAGCAGCCAGATTT
CTCCTTATGTACAGACATTAGAAGGAGAACAAGATGCATCTCCTTTGGCC
AATCCAACTTGGGACAAGATGGTTGATTTTGTTAGTCAGAAGAGGGTGTT
TCTAGCAGCCGCCGCCTTTACTCACACACACAAGCACAAGACCATTATCA
ATTATACTGACGCCATAAGTGTCGAACCCTATGTCTTCCTGGTTGCAAGA
CCACGCGAGCTTAGCAAAGCCTTACTCTTCACCGCACCATTCACTACGGC
GACGTGGCTCTGTATTGGAGTGTCCATTTTGTTCACTATACCCCTCCTGT
ACACACTCCATATGGTGAGCCCTTATTACGAACATTTCAACATACGAGAG
CAAAGCGGCTTTCATCGATTCTCAAACTGTGTGTGGTACATCTACGGGGC
GATCTTACAACAAGGTGGTGGTAAGTTACCGGAGGCAGACAGCGGTCGTC
TGGTCATCGGCACCTGGTGGCTGTTCGTCCTGGTCATCGTGACCACCTAC
TGTGGCAATCTGGTGGCCTTCCTAACGTTCCCTATGATGGACACTCCGGT
AACCAATGTAAATGAACTGCTAGATAACCAGTGGCGGCTGAGCTGGGGCA
TGTCCGAGTATTCGACACTGCACACAACACTGCAGAATTCCCAGGACAAC
CAAAAATTGAAGGAGTTGTACGACAAGATGGAGACTCATCGTGACATCAC
GGACGCGGTAATATCTCGTATAAGATCCGGCACTCATGTGTTCATACAGC
GGCGCACAACTCTCTTGTATCTGCTCAAGAAAGAGTACCAGAAGAGTAAC
CGCTGTGACTTTACATTGGGAGATGAGGAGTTTGCAGAAGAGCGGCTCTC
TATGGTCCTAGCACAAGGCAGTGCCTATATGCCAATTATCAATAGAGAGA
TAGGTAGGATGCACAGGGTGGGGCTGATTCACAAGTGGCTCCTGGATTAC
TTACCGGACAAGGACCGCTGCTGGAGTGAGCCCAAGAACCAGGGCACTAA
CCATATGGTCAACCTGAACGACATGCAGGGGTCTTTCTTCGTACTGATAA
TCGGTTGTGTCGCAGGAGGAATCACTATCCTGGGAGAGTTCTGCCTCAAC
ATGTACAAGACTTCCAAGGAAAGGAGTTTGATCCAACCGTTTGTGTCCTG
A

>IR10
GAAATACAGAGACGACGAAGAAAAG
>IR11
ATGGACTATGATACAGGGTATGAAGAAATCTTGCAGGGCCTGATGATTCT
GCCAAACAGTAATGTAGAGGTGGTGGAGTTGAATGCAGCGAGAGGAGTGT
GGCAAACGTTCACCCTGTATAAAATTCGTCATAACAGTACGAGAATGGTG
AGGACATTGGAAAGAGAAGGTTCTGTCGATGACAAAGGACTCCGTTTACA
CATTCTCAAGAACAGACCAAGCCGTGACAACTTCAATCGAGAACCAGTGA
ACATTGTTGAAGTTTTGATTCACCTAGACAAGTTCCAAGGATGGGATAAG
AAGTCCTCCATGGATTTGGTTGACTTGTACGGTTGGGCAAGTCACGAGCT
CCTCCAGTGTCTCGCCTTCTCTCTCAACGCTACACAAGTGTCCTACCTGA
TAGATGACTACGGATGGAAGCACACGAGTGAAGCGGGTAGCTTCCTGGGA
GCTGGGGGACTCATCAAGTTTCTCAGAGAATCAGAGGCGGATACGGGCTC
CGCGCTGTTGTTGAGAAATGACCGCCGGGTGTATGTCAACTACGCCGCCA
CTGTGTTTCCTGTCACCACTTCATTTCTGTTTCGGCAACCATCCCTCGCC

TCCGTGTCCAACATCTACCTGAAGCCATTTTCCACCGGTCTCTGGCTCTG
TACGCTCGTACTCCTGGCGTTCATAACCGTCACCTTGACCGTGCTCCTCT
CCCTGTCTCATCGCTATGGGTTCGAGACCCAGTTCGGCCGGATTTCTCTG
CCCGATGTTCTCATGTCCGTTCTCGGCGCGATTTGTCAACAAGGTGTGGA
CAAGGTTCCTCACAGTGCCTCCGGTCGCATGACGGTCCTCGTGACATCTA
TCACCTGTCTCTTTCTCGTTACCTCCTACGCGGCCAACATCGTCGCCCTC
ATTCAGACGCCCAGCACCGTGATCAACTCCGTCACGGATTTGGCCAACTC
GCCGTTCACGGTCAAACTCCACGAGTTCCAGCACAACCGGGCTTTTCTCA
GAGATGTCAAAAAGAATCCAAAACTGAATAAGGAGGCCAAATATTTGTAC
GACAAGAAGCTACAGGGTAGATCCGAGGCCTCTCTATTCGTGAATGCCTC
CACAGGAATCGAGAAGATTCGACAGCCCCTGTATGCTTTGCTGGTGGCTC
TAGAAAACTTCGCCTACTTCTACATCAAACAGACGTGGCGCGAGGAAGAA
AAATGCGGCCTCTCTGAACTGAGGGCTTTCCCCAAGAACTATATGGTGCT
ACCTGTGAGTAAGCAATCCGGATATGCTGAGCTATTTGCTCAGAAATATA
TTCTGTTTCGTGAGGTGGGTCTCATACAGCAAACCACACAGAGGTGGCTG
GGTACGAAGCCAGAATGTAGATCCGGCACCTCACCCCAGTTTGTATCCAT
CGGAATAATTGAGTTTGCCCCGGCCCTGAAGATCTACGCTGTAGGTATAG
CGGTAGCCCTGCTGGTCTTCGTCGGAGAACTAGTCAAATATTCTCTGGAT
GGGAAACTGCCCCATCCTCACGGCGGTACTCCTCAACAAGAGCAATCGCC
GGATAGGAGGAGAAGAGAGGGGAGCAGACGGTTCCGTCACCCGTCCTGTA
TTCAGGAGGCCCGGCTTCGAATCCCTTGCGCATAG
>IR12
ATGAAATTATCAGTGATATTTTTCGTGTTTTTATTTATTACTATTAGCTG
GTGTCCAGTGCGATCTATTGTACCAAAGAGCATCATGGATAGATGGCGGG
AGATAGAGCGTCGCTCCAAGCTGAAGTTGCTGATCTCCGACATCATGTTC
AAGACGGTGGACACCCTCAACTGTATGCTGGTGCTGACTGACTCGTTCTA
TCAGCCTCTGTTCACGAATGACTTGTATGGCCGCCTTCATGAGGTGCCTT
ACTTCAAGATTCGCATTGGAGACAATGAGGACCTATTGGCCCCCAACTAC
AAGACTCTGGCCGTTCTTAAGGAGGCCAGGAAATCGGGGTGTAACACCAA
CGTCATTGTCGTGGCCAATGGAGACCAACTGGGCCGCCTGCTGAGATTTG
GGGACAGGCACCGTGTCCTGGACACCCGGGCTCGCTATATTTTCCTGTTC
GACCCGCGTCTTATTCACCCCGGACTACACTACATCTGGAAGAAACTGGT
CAACGTCATATTCCTGCGGGAGTACGCGAGCTCTGGGCGGTACGAAATCG
TCACCACGCCCCTTCCCGGTGCCCATCCAGGAGGTGCTGGTGCCCCGCCGT
CTCGACTACTGGCACAACGGAAAGTTTCAGGTGAATTCGGATCTATTCCG
AGACAAGACGTCTAACCTCCTGGGACAAACACTTCAAGTCGTTACGATTC
AACACATTCCCGCCTCCATCAAGACAGTCATCAGTCCCTCTGATTCGACC
AGCTCTATAGCCGTCAAATATAGTGGACTGGAAATAGAGATCCTCCGCGC
TCTTCAGAACGCCATGAACTTCGCCACCAATGTCTACGAGCCGCAGAACA
GCGACGCGGAGAAGTGGGGTCGCCCGCAGCTGAACGGATCCAACTCAGGT
CTGCTCGGCGAAATCGAAATGGGCCGAGCCGACATCGCTCTGGGCGATCT
CCACTACACGCCCTACCATCTCAAGCTGATCGACCTGAGCCTGCCCTACA
TAACGCAGTGCCTGACGTTTCTGACCCCGGAGATCGAGACGGACAACTCG
TGGCAGACCCTGATCCTCCCCTTCCACCTAGACATGTGGATAGCTATCCT

CATCACAATTCTCCTCGGCGGCTTTCTCTTCTACGCCCTCGCCACTTTCC
ATCTCTACATAGAGCACAAGGACTTCTCTCGACCTCCACCGAGCCCCCAA
CCCAAGCCACCCTCCAAACTATCTCTCGCCTGGAATAGACTGCGCCAGAA
ATATTTCAAACAGGCCACGCCCAAGCCACGACGCCATATTTATCTAGAGA
AGATGAAAAAGAAACATGGCGTCCTTCAGTTTATCAAGAGAAAGTACTCG
GATATGTCGGAAAAACTGAGCAACACCATCAATGAGATGTTGAAAGAAAA
GCCTAGTACAGAAGGTCTCTACCTGTTCAAAGACATCGACAACAGTTTCC
TGTACACTTTCAGCATGTTGCTAGTCGTCTCCCTGCCTAAAATGCCCACG
GGCTGGGCTCTGCGCATGCTCACTGGCTGGTGGTGGCTCTACTGTGTTCT
CGTGGTCGTCTCCTACCGGGCGAGTCTGACAGCCATACTAGCCAATCCGG
CGCCGCGAGTCACAATAGACACTTTGGACCAACTCGCGAAGAGTTCTCTC
AAATGTAGCGGCTGGGGGCAGCAGGTCAAAGAATTCTTTTTAACGTCATT
AGACACGGCGGGTCAGAAGATCGGGCAAAGGTTTGAGGAGTTGAATGACC
TTGAGAAGGTGGTCAAGCAAATATCGGACGGCAACATGGCCTACTACGAG
AACATTTACTTCCTCAAGGATATCGTCATGAAATACTCACACCGAAGCGG
CAAAGATGACTCTAAAGACGCCGCCCTCAACACCACCGCCCAAACATCCG
CCTCCTCGCTGCACATCATGACGTCTTGTATCATCAACATGCCCATCTCC
CTGGGCCTCCAGAAGAACTCCCCGCTCAAGCCTCGCGTAGACACCTTCAT
GCAGCGGGTCATCGAGGCTGGCCTCGTTAAGAAGTGGCTGAATGACGTTA
TGCTGAACATCACGGTGTCGGAGACCGAGGACGGAGAGGACGAGGTCAAG
GCGTTGATGGATTTGAAGAAGCTCTACGGAGGCATTGTAGTGCTCATCGT
TGGATCAGCCCTGAGTGTTCTGATTCTACTGGGTGAACTGGCGCATTGGT
ATCTGGTCACCAAACGAAACCCTCACTACGACCAGTACAATGTGGTGAAG
TACTACAAAGCAATGAGACACTATTGA

>IR13
ATGAAAATGGCCAAAGACTTCAAAGGGCTCACCGGCAAGATAAAATTTGA
CCAAGAAGGTTTCCGAACTGACATCGAATTGGAACTGGTGGACTTGACGC
AGAATGGTTTGCGTGTGACGGGCACGTGGAGCACAAAAACCGGAATTAAC
GTTTCGGCGATACCCAAATCGGAAATGGTGCCTGGTGGGAAAGAATTCGA
TTTGAGGAACATGTCGTTTGTCGTCATCACGGCGTTGACAAAA

>IR14
ATGCAGCAGGGATCCGACATTTCACCCAGATCGATCTCGGGTCGCATCGT
GGGAGCTGTGTGGTGGTTCTTCACCCTCATCCTCATCTCTTCGTACACCG
CTAATTTAGCAGCATTCCTCACAGTGGAGCGGATGGTGAACCCTATCAAC
TCAGCGGAGGATCTTGCCGACCAGAGTGATGTCCTGTATGGCACCGTCAG
GGATGGTGCCACGTATCATTTCTTTGAGACGGCCACATTCGACACGTACC
GCAAGATGTACCACTTCATGCAGATGCACAAGGACGAGGTGTTCGTCTCC
TCGGAACGAGAGGGCATCAAGAAGGTGCTGACGTCCAAAGGCAAATACGC
GTTCCTCATTGAAAGCCCGTCCAACGATTTTGAGAACTCGCGGCAACCCT
GCGAGACAATGAAAGTTGGCAACAATCTGGACGTAAAGGGGTTTGGTGTA
GCCACGCCCATTGGGTCTTCTTTGAAGAATGACGTGAACATGGCCGTACT
GAAACTGTTGGAAGAGGGAGAGCTGGCGAGGCTGCAGAAGAAATGGTGGA

TTGACACGAGCGAGTGTAAAGCCAGCAAGTCTCAGGACTCACACAGCGAG
CTAGCTCTGAGCAATGTGGCGGGAGTGTTCTACATCCTGGGCGCTGGTCT
ACTACTAGCTATGGCTGTGGCTCTGGTAGAGTTCTGCTACAACACGCACA
TAGAGGCCTCTAATAATAAAGTTCCAGTGAGTGATGTGATGAAGAACAAG
GCTCGAATGACTATTGGAGCGAGGGAGTTTGATAACGGCAGAGTGAGTCA
AATGTACTATGCTTCGGGCAACACTCTCAATCCCCTGGAGGATCAAGTCC
ACAGCAACACTCACACTCAAGTCTGA

>IR15
ATGTTGCAACAGGGTTCCGATGTATCACCTCAGGCGGTGTCCACGCGTTT
AGTGGCAGGGATGTGGTGGTTTTTCGCGCTGATCATGACCTCGTCGTATA
CTGCCAATTTGACAGCGTCCATTACCAGCGGACGCCTGGACACGCCAATC
AAAAACGTCGATGACTTGTCGAAAGACTCGAACATCGAGTACGGCTGTTT
CGAGGATGGATCGACCGCCAGTTTTTTTCAGAAATCGAACTTGAGTTTGT
ACCAGAGGATGTGGAGCGTGATGGAAGCGTCTAACCCGACTGTGTTCACG
AAGAGCAATCAGGAGGGCGTTGACCGAGTACTCAAGGGTAAAGGCCGATA
TGCGTTTCTCATGGAATCTTCGAGCATCGAGTACCAGACAGAACGTAACT
GCAACCTGATGGAAATCGGCAACACCCTCGACTCCAAAGGGTACGGCATA
GCAATGCCGATGAATTCTCCGTATCGTACTCTGATCAGTGAATCGGTGTT
GAGATTACAAGAGTCTGGATTCATGCGAGAGCTCAAAGACAAATGGTGGA
AAGTCCAAGGAGACAATAAATGCGAGGAAGAAGATGAAAGCGACGAACTC
GGCTTTACGAAAATTGGTGGCGTTTTCGTGGTACTGGTTCTCGGGTGTTT
AATTGCGTTTATGTTTTCCATATTGGAGTTTTTGTGGAATATCCGGAAAG
TGGCTATCGAAGAAGAGATAACACCAAAAGAAGCGTTGATTTTAGAATGG
AAATTCGCAATGAAGTGTGACGGAGGAGTCAAGCCACTAAGGCGGCGGCA
TCATATCGATACCGAAACTAACAACTCTGATACAAGCTAG

>IR16
ATGTGGTGGTTTTTCGCATTAATCGTAACGCAGTCATACACCGCTAATTG
GACAGCATTTTTAACATCGAGTCGAAAGGAGTCTGCCATCAAAAGAGTTG
AGGATCTAGACAAACAGTCGACCATCAAATATGGATGTGTTAGAGGGCAA
TCGACTGCCGGTTTTTTTGAGAATTCTGATGTGAACCTTTATCAGAAAAT
GTTTAGCGTGATGGAAACATACGGTGACACAGTGATGATGTTTGACAATA
AACAGGGTGTAGACCGTGTTAAAAAAGAAAGAGAAGCCTATGCTTTTTTT
ATGGAGTCTAGTACAATTGAGTATGAAGTCCAGAGAAATTGTGATTTGAC
AGAAGTAGGAACCTGGTTGGATAACAAGGCATATGGAATTGCAATGCCTT
TTAATGCTCCACACAGAACGGCAGTTAGTATGGCGTTATTAAAACTTTCC
GAATCTGGGAAGCTAATGGAACTAAAAGATAAATGGTGGTCGGTATCTGA
AGAAAAAATGTGTCCAGTACCTAAAAAAGATTCTGCAGAGCTAGACGTAA
ATGAAGTGGGAGGGATGTTTGTCATTCTTATTTTAGGATGTATGTTGGGA
TTTCTATTCTCTCTATTGGAATTCTTATGGAACATTCGAAAAGTGGCAGT
CGCAGAAAAATTATCGCCGTGGGACGCATTCAAGTTGGAGCTGAAATTTG
TCCTTAAATGCCACCATTCAACTAAACCAGTGAGACACACTACATATTCG

GACGAACCGTCTTCAGAAGACTAA

>IR17
ATGACGGGGGTAAGAAAGAAGACAAACTACTTCCCTTCGTTCATGACTTT
ACGAAAGCCCAATATAATGGATTACAAAGGTCGTCAAATAGTTGTTTCTA
CATACAAATGTCACCTGTTCAACTCTTTACAAGCTTTACGTGAATTTAAT
ACACGTCAAGTTTCTAGAATTCCGAGTTCAAGTGATAATTTCTATGGTAT
TGAAGAACGTATCTTCTTGGAAATAGCCATGCGCATGAATCTTACATGGA
AGTTACGCATTCCCAACGAAAAACGAATATTTGGAATGGAATTATCTAAT
AAGAGCTTGGGTGGCGGGCTCATGAACGATCTTTATAAAGGACAAGCTGA
TATAGGATTCTGCGCTCTGTGGTATGACCAGGTTAAGATCAAATCCTTTG
ATATGAGTACCTTTTGGTCCGCTGTCTGTCCGAAATTTCTCTTCCCAAAA
CCGAAGAAGATGAAAACCAATTGGCGAAACTTGTTCGAACCGTTTAATTG
GAAAATTTGGACTCTCTTATTCTCATGCATTCTGCTCCAGACCTTCGTAT
TGATGTGGATTTCGGACAAGGCTCTAAGACTTAGAATTCTTTCTATTCGA
TATTACTCACATCCAACCAACTCATTCCTGGAGGTGATTAGAATTTTGTT
TATGGCTGGCTGTACGTCATCCAAGAAAATGCCCCGCCTCGGACCATTGC
GTCATTTAATATCTTGGTGGTTTGTGTTCAGTCTGGTAGTGGCTACGATC
TACTCTACTAGTTACTATTCACATCTCACATCGCCTGAATACACTCGCAA
AATCGAGGGCATTCAGGAATTGTTGGAGCAGAAAATTCGCTGGGGAAGCC
GTTTTCCTCCTCCTCACTCATGGATATTTCATTCCTCGAATCCCATTCAT
CAGAAATATAGTGAGCTTTATGAGCTAGAAAAATCAGTTGAAGACAGAAT
CGAAAGGCTCGAAGCAGGAAATTACGCTGTGTATTGTACTAATGTCAACA
ATTTATTCTTCATGGATGTAGATGAAGTATCGCCAAATCTTCTCAAGGAT
TTGCGATCCTTCCGCAGATGTCTGAGTCAGTACTTTGTTAGCTTTGGACT
ACGTTTAGGAACCTCATATCTAGAGCAAGTGAACACAATCATACTCCGAC
TTCTACAAACGGGCATTATAGACTACTGGCTCATCAGAGTTATACTTCTC
CATTACCATCGCAACCCGTTCAACCGTGTGTATGAGTATAAACCCCAAAA
CAACAAGAATGGTCATGATCGTCTTACTCTATCTAGTTTGGAGGTAAAAT
AA

>IR18
ATGGTCAAACTTTCCAGTAATACCCTGGAAGGAAACGACCGATACGAAGG
TTTCGGAATCGATTTGATCAAAGAGCTCAGTGAAATGAGTGGTTTCAATT
ATACGTTCATAATACAAGAAGACTTCAACAGCGGATATATCGACGAAAAA
ACTAAAAAAATGGAACGGAATGATCGGGGAAGTCATAAACGGACAAGCAGA
TCTCGCGATTGCCGATATCACTATCACTAGACAACGAGAACACGACGTGG
ATTTCACCAGCCCGTTTATGAATCTCGGCATCAGCATATTGTACAAAAAG
TCGACAAAGAGTTCACCGAGTCTGTTTTCATTTTTGGCACCGTTCAGTTC
CTTCGTCTGGCTTTGGGTGATAACGGCCTACTGCGGCGTGTCTGTTCTGC
TATTTATCATGGCCAGGATCAGCCCTTACGAGTGGACCAACCCTTACCCG
TGCATTGAAGAGCCTGAGTTTTTGGAAAACCAGTTCAGCCTGTCAAACGC
ATTTTGGTTTACGATCGGATCGCTGATGCAGCAAGGTTCCGACATCGCTC
CGATCGCCGTGTCTACTAGACTGGTCGCAGGTATCTGGTGGTTCTTTACG

CTCATTATGGTTTCGTCTTACACTGCCAATTTGGCTGCGTTTTTGACCGT
CGAGAGTGTGTCGGAACCTTTCAAAAACGTAGAGGATCTAGTGAACAATC
AAAATGTTATCACTTTCGGTTTGAAGAAAAGAGGATCTACTGAGGAATAT
TTCAGGGAATCAACAAACCCTACTTATAAAAAAATATTTGATATTTTACA
AAAAAATCAGCCCTGGTACACTTCGAGTAATGACGAAGGCGTTGAGAAAG
TACTCAGAGAAAATTATGCGTTTTTCATGGAGTCTACGTCAATCGAGTAC
ATGGTCGAGCGGAACTGCAAGTTGGCCCAAATCGGCGGACTGTTGGACAA
CAAAGGCTACGGGATTGTGATGAAAAAAAACGCGAGTTTTCGAAACGTTC
TGAGCGCAAACATATTGAGCCTGCAGGAGAAGGGCAAACTGACGGCGTTA
AAGAACAAATGGTGGAAAGAGAAACGCGGGGGCGGAGCGTGTCAGGACAC
CGACAATAACGAGGCCAGCGAGTTGAGCATGAAAAACGTGGGCGGCGTTT
TTATCGTGTTGTGCAGTGGCGTCGGCGTCGCCGCCATACTGGCAGCGATG
GAAATGTTCTGGACCCTGTGGAAAACGACTAGCAAAGAAAAGGTGTCGTT
CAAGAGCGAGTTCAAAGACGAACTCAAGTTCATCGCCAAGTGTCGCGGTT
CCACGAAACCGGCTCGGAGGAAGCAGAACAGCAGCGCGGACAACTCGAAC
CCGACCGGATCCAATCAGTACATGAACTCCGACCGACACTACGACTACGA
GTCGTGA

>IR19
ATGTTGTGGGAGCACATGCAACGCTTCTCGGTCAAAACGGTGGCCGAGGG
AGTACAACAGCTCAAGAACGGAAGTTTAGACATTCTCATAGCAGACACAC
CGATATTGGACTACTATAGAGCCACCGACCATGGCTGTAAGCTGCAGAAA
ATCGGGGAGTCGGTTAATGAAGACACGTATGCTGTGGGACTGACCAAGGG
GTTTCCACTAAAAGACAGCATCTCCGCAGTGATTTCCAAGTATTCCAACA
ATGGGTACATGGACATTCTGCAGGAGAAATGGTACGGGGGACTGCCCTGC
TTCAAACTAGACACAGATATGGATATGGCTCAGCCCAAGCCTCTAGGCAT
TGCAGCTGTGGCAGGTGTGTTCCTGCTATTGGGTGTTGGCATGGTAGCTG
GCTGCATGATCCTCTGCATGGAACATCTATTCTACCGGTACACTTTACCT
ATACTACGACACAAACCAAAGGGCACTATATGGCGCAGTCGAAATGTCAT
GTTCTTCAGCCAAAAACTGTACCGATTCATCAACTGTGTGGAGTTGGTGT
CACCTCATCATGCAGCGAGAGAGTTGGTGCATACACTAAGACAGGGTCAG
ATCACCAGCCTCTTCCAGAAGAACGTGAAAACGGAACATGAAACGACCAG
GAGAAGAAAAAGTAAAGCACAATTCTTCGAGATGATACAGGAAATCAGGA
GGGTTCAACAGCAAGAAAAACTGGACAACAACATGAAGAATAGTCCTAGC
AAAAAGAATCACAAGCGATCCAAAAGTCCCCAAGTTTTACTGAGCCCACC
TGAACTCATAAAACAACAAAGACGGCTATCTCCAAGCAAACTGGACTTCG
CCCGGCGATTCAGCAAGGATTTCTTCCGCTCGAAAAGCTCAGGCAACTTG
AACGCTGCGACCCGTCGCATGAGCAGTGACATTGGCGTCGCCACGGGTAG
ATTCTTGGACTTCCATAGCGCCCAGACTATAGGACGACGTCTGAGTCACG
GGGTCGCCAACAGTCCACCCGATTTAAACAGTCGACGATCAAGTGCCTTG
ACTCCACGACGGGAATCCAGTCCTGAACCAATCTATGAAGTCGAACCTAC
GTCCCCTAATATAAAATTAAGTCCCCTAATGCCTAATTTACCCCCTACTT
TAACGTTGCCGAGAGATATTTCTTCTGCTTCCTTACACGAAATTGAACCA
ACGACTCCAAATAGACTGATCAAGTTACCGTTACCCAGAAATATATCTTC

CGCTTCCTTGACGCCCAAATATGACACCAGTCCTCGATCTCCAAGCTCGA
GAAACTTCCCTGCAATTCCGTCACGGGATATTGGAAGCGCTGCAAAAAAA
TTCAGCTATGTTGACGTGAATTCAAGTTCATTTAAGAGTAAAATATCCAA
GCTACAAAATAATAATGGTGTCAGTCCGAGGCAAGTTCACAGTACCGAGA
GTGCAACTGTGAGGCCCATGGTTGTGTCACCTGGCTCCCCAAGTAATACT
CTAGAAAACACTAATACTGGTAACAGTGGCGAGGATTTTACACTCAACAG
AGAGAACTGGGCAAATGTATCGGGAAAGTCCGTGGAACACATCATCGAAA
TCATTATTCCAGATGAAAAACGAAGTAAATTGGACACGGTGAGTAAGGGC
AACAAAGCAGATAAAGTTAAAAATGTACCAAAACCGATCAAAGAGAAAAA
AATGAAGGCAAGTGTAGCCAACCAAGACCAGTGTTTTCCTGTGGTGAAGC
CAACCATTGTTTCTTACATTCCCAGAAAACCAGATAGTGATTGCTCGAAT
GGTTCCACTCATTGCGATCAAGATGTGTCGAAACCAGAATCACCTCTGCA
GAAGCTGAGTCGGGAAGAGTTGTTAAGTCTGATAGAAACGCCTGAACAAG
AGCTCAAGAATTCCATTTTAGAAGCTTTGAAGTTCAAAGATCCAACTTGA

>IR20
ATGGAACTTGTGTCTCGTGGTGTTCTGTTCTTGCACGACAAGCATTGGCT
CAACTTCTTCATGCTGGGCATGGAGAGGGAGACGGATGAGGACAATCGCT
GCCAGTATGTCCTCACCACCGACATTTACCTCACCCGTTCCCTCAGCTTC
GTGTATCCCAAGCATAGCATCCTGCCTCTTCTGTTCGACTCCATCATGTT
GTCCTACGTCGAGAGTGGCATCATCAAGCATCTGTTGACCAAGGATCTGC
CGCAGGCCGCAATCTGTCCGCTGGATCTCGGCAGCAAGGAGCGACAACTC
CGCAACCCGGACCTGGTCACAACCTATTACGTGGTG
>IR21
ATGCGAGTGATGGAGTTAGACGGACTGACCGGAAAAATGAGATTCAATTC
AGAGACAGGTAACAGGAATTACTTTAAAGTAGACGTGGTCCGTGTGCAAG
AGAGTAAGAAGCATCGTTTGGGATCTTGGGATCCGGTGGAGAGAATAACG
CTGACGAGATCGGCGTCCGAAATCTACTCAGAATTTGCCCAGTCGATTAC
GAACAAGACGTTTATTGTCGTCGGAAAATTAGTTCAACCATATCTGATGA
GATGCAACGCCACTGAAAAGGGAATGGACAAAGACGAAGAATGTTTCGAA
GGATTTGCGTACGACTTGGTCGAGGAAATGGCCAAATACAATGGATTTAA
ATTCAAGTTTACCACCAACCAAGATTACGGAATTATGAATCACAAGACTG
GCAAGTGGAGTGGTATGATTGGAGAGTTACAGTCCATGCGAGCGGATTTA
GCTATCTGTGACCTCACGATCACTTTTGACCGGCGGAACGCGGTGGACTT
CACGACACCGTTCATGACGCTGGGCATAAGCATACTTTACGCTAAACCTG
AGAAGAAGAAACCACAGCTGTTCTGGTTCCTGAACCCGCTGTCATTCAGC
GTCTGGATGTACACAGCGACCGCATATCTGGGTGTGTCTTTGTTCTTGTT
CATGTTGGCCAGA
>IR22
ATGAACTACTCGCTCCACGTGGTGGAGATGACGGTGAACAGTGCCATGGT
CAAGGTGGCGGAATGGAGCGATATCAACGGCTTCATACCTGACGCCAGTG
TGAGGTATCAACGTCAGAAGATCTACGGCGTGTATGACAACAAAACATAC
ATCGTGACCGCTATCCTGGCTGAGCCCTACCTGTTTGAGGTGAAAGACCA
TCAGGATCGAGAGGGTAACGATAAGTACGACGGCTTCTGTAAGGATCTGG

CAGATCTTCTGGCGCAGAGGCTGAAGATTTCCTATAAGTTCCATCTGGTG
AAAGACAACAAATATGGAGCACCAAACAAAAGTCAGCCTTCCGAGTGGGA
TGGTATGGTAGGGGAGCTGATCAGAAGGGAGGTAGACATGGCTATAGCTC
CTCTGTCCATCACTTCCGAGAGGGAGCGGGTTGTTGACTTCTCCAAGCCC
TTTATGACGTTGGGCATCAGCATTATGATCAAAAAGCCCAACAAGCAAAG
ACCTCGCGTGCTCAGCTTTCTCGATCCCCTCAGTGAGGAGATCTGGGTCG
CTATCATCTTCTCCTATATTATGGTGAGCGTCGTTCTTTTCCTCGTGTCG
CGTTTCTCCCCCCACGAGTGGCGTCTCCTCAACTACTCGGACCCGGGTCA
CCCCCACCAC
>IR23
ATGCTGGGCGCTGTCAGTCCCGACTCGTTCGATACGTTTCACTCGTACAG
CAACACGTTCCAGATGCCCTTCGTTACGCCCTGGTTTCCTGAGAAAGTCC
TGACTCCGTCCTCCGGCTTCCTGGACTACGCTATCAGCATGCGTCCGGAC
TATCACCAGGCAATCATTGACACCGTCAAATACTACGGCTGGAAGAACAT
CATCTATATGTATGACTCACATGACGGCCTGCTCCGTCTGCAGCAGATCT
ACCAGAGTCTGAAACCAGGCGCGGACAGCTTCCAAGTTGTCACGGTCAAG
CGGATTCAAAATGTGACGGAGGCCATTGATTTTCTGCACGATCTAGAGCT
GCTAGACAGATGGGGCTTCAAGCATATCGTGTTGGACTGTGCCACAGACA
TGGCCAAAGCCATAGTCGTGAACCACGTGAGGAAAGTCACCCTGGGAAAG
AGAACCTATCACTACCTGCTCAGTGGATTGATCATGGATGATCGCTGGGA
GACGGAGGTGATAGAGTACGGTGCCATCAACATTACCGGGTTCCGACTCA
TCAACAACAACAAGAAATTTGTGAGAGAATTCCTGGACGAATGGAAGAAG
CTAGATCCCAAGACTTACATCGGAGCGGGCAAAGACTCGATATCGGCTCA
GTCAGCCCTCATGTACGACGCAGTCCTGGTCATAGTTGAGACGTTCAACA
AGTTGCTGAAGAGAAAGCCAGACCTGTTCAAGGCTAACGTGAACAGGCGC
GGACAGATGATGAACAATGGAAGCAGGATGGACTGCAACACAAGCAAGGG
ATGGGTGACGCCCTGGGAACACGGAGACAAGATATCCAAGAACCTGAGA
>IR24
ATGGACTGGAAGTCATTCACGTTACTCTATCAGCGACCAGAGTGTCTTCA
GAGATTACAAGACTTAATACAAGATTACTCGGGAAAAACAAAACCTAATG
ACAAACAGGTAGCGGCCATCTCCATATTACAATTGCCCGAAGGCAATAAT
TTTAGACCGATTTTAAAAGACATTAAGAAGTCTTTAGAAGGTCACATTGT
ACTCGACTGTGATGCAGATTTAATATTAACTGTATTCAAACAAGCAAAAG
AAGTTAACTTGTTGGATGACTATCATAGTTTTATCATAACCTCATTGGAT
GCACATACAGTAGATTTTAGTGGTATTGTGCAGAACTTGCGTACAAATAT
AACAACTGTAAGGTTGATTGATCCACTAAGCCCATTTGTGGAAAATATTG
TGAGAGATTTGAATTTTGTTCAACAAAGAATGAACGTAAATATGGAACCT
TTGAAAGCTGACAAACTTACTGTTAACTCCATACTCATATATGACGCGGT
AAATGTGTTTGCTAAAGCTTTAAAAGGCCTAGGTATGATAAATAAAATCA
TCACTGAACCACTACAATGTAAGAACTCGCCATTTATCCCCTGGTCCAAT
GGATTTAAACTTATAAACTTTATGAGAGTAATTGAAACAGAAGGCTTGAC
TGGTCTGTTACGCTTTGACAACAAAACTGGTCATCGATCATATTTTACAC
TAGAAATGGTAGAACTGGTTGATACTGGTTTCAAAAAAATTGGCTTGTGG
GATCCAGAGAGAGGCATGACTTATACGAGAACCAGCCTTGAAATGCTTCG

TGATTTATACGCCGGGAGTAAGAACAAAACATTTATTGTTTCAACAAAAA
TTACTGAGCCGTATTTGATGTTAAAAGAAGGCCACAATAAACTAGAAGGC
AATGACAAATATGAAGGATATGTGGTGGATCTTATACAAATGATTGCTAA
AGAAAACAACATGACTTATGAATTTCGTTTGAGAAGTGATGGAAATGGGA
AACGTGACAAAAAAACAAACAAATGGAATGGAATTATTGGTGAAGTACAA
GAAATGAGAGCCGACTTGGGGATCTGTGATTTAACAATTACTCACGAAAG
AAGGTCTGCTGTGGATTTTACCATGCCTTTTATGAACTTGGGTATCAGCA
TATTGTTCAGTAAACCAGAAGAACCGCAAACCAATCTTTTTTCATTTACC
CAGCCGCTTTCTTTTCAAGTATGGATATTTACAGCAACCGCTTATTTAGG
CCTGTCTCTGGTATTATTTTTCTTGGCTAGAATTACGCCCAACGAATGGC
AGAACCCTCACCCGTGCAATCCTCATCCACAAGAATTAGAAAACTCCTTA
TCATTGCTTAACTGCCTATGGTTTTCTATGGGTTCCATTCTTTGTCAAGG
TAGCGATATTCTGCCTAGAGCTTTTCCAACCAGACTATGTGCTGCTATGT
GGTGGTTCTTTGCTCTTATTATGACTCAATCTTACACTGCTAACTGGACT
GCATTTTTAACATCGAATCGGATGGAAACTACTATAAAAAATGTAGAAGA
TCTTGACAAAAAGGGAGGCACAGATAGCATCAAGTATGGATGTGTAACTG
ATCAATCTACAGCCAGTTTCTTTCAAAACTCTGATGTAAATTTATACCAA
AAGATGTGGAGTGCTATGGAACTAAATGGTGATTCTGTGATGGTATCTGA
TAACAAACAAGGGGTGGATCGGGTGAAGAAGGAGAGAAATCATTATGCTT
TCTTTATGGAATCAAGTTCTATCGAGTACGAAGTGCAGAGAAATTGTGAT
CTTACAGAAGTGGGATACTGGCTAGATAACAAGGCCTATGGTATTGCGAT
GCCATTCAATGCTCCTCACAGAACACTTGTTAACATGGCTGTGTTAAAGC
TTTCAGAGTCAGGAGCGTTGATGAACCTAAAAAAATAGATGGTGGTCAGTA
TCTGACAATAAAAGGTGCAAGGATTTAAAAAAAGACTCGGCTGAACTTGA
TGTCAATGAAGTTGGTGGTATGTTCGTCATTTTAATACTTGGCTGTCTGA
TTGCATTTTTATTCTCTATACTCGAATTTTTATGGAATATAAGAAAAGTG
GCTGTTGAAGAAAAGTTAACACTATGGGAAGCCTTCATGGTGGAGTTGAA
ATTCGTGCTCAAGTGTCACGGAACATCCAAACCAGTGAGGCATGTAGAAG
ACAGTTCG

>IR25
ATGCCATTTATGAACCTCGGTATAAGTATTCTGTACCGGAAACCAATAAA
ACAACCACCGAACCTATTTTCGTTTTTGTCTCCTCTGTCGTTGGACGTGT
GGATATACATGGCCACGGCGTACCTCGGCGTATCGGTTCTTCTCTACATA
TTAGCCAGGTTTAGTCCGTACGAATGGGAAAATCCACATCCTTGCAACTC
CGAAGCTCCAGATGTTTTTGAAAACAAGTTTTCCTTGAACAATTCACTGT
GGTTTACGATCGGCTCTCTGATGCAACAAGGATCTGATATGGCTCCTAAG
GCCGTTTCCACCAGAATAGTGGCCGGTATGTGGTGGTTTTTTACTTTAAT
CATGATCTCGTCGTACACTGCTAACTTGGCGGCGTTTTTAACTGTGGAAA
GAATGGACTCGCCGATCGAAAGCGCCGAAGACTTAGCTAAACAGACGAAA
ATAAAATACGGTGCACTTCGAGGTGGTTCAACTGCTGGATTTTTCAGAGA
TTCAAATTTCATTACTTACCAAAGGATGTGGTCGTTTATGGAATCGTCAC
GACCAAGCGTATTCATGGCGTCTAATAACGAAGGAGTAGAAAGAGTTGTA
AAGGGAAAAGGAAACTATGCCTTTTTAATGGAATCCACTAGCATTGAGTA

CGTGATCGAGAGAAATTGCGAGCTCACTCAAGTCGGAGGACTTTTGGATT
CAAAAGGTTACGGAATAGCAATGCCCCCGAATTCTCCATATAGAACAGCG
ATTAGCGGAGCGGTGTTAAAACTTCAGGAAATCGGTAAACTACATAAGCT
AAAAACAAAATGGTGGAAAGAAAAAAGAGGTGGTGGAGCTTGTCGGGACG
ACACTTCAAAGTCCAATAGCGCAGCCAATGAGTTGGGCCTGGCTAACGTG
GGTGGCGTGTTTGTAGTGCTAATGGGTGGAATGGGTGTAGCTTGTGTAGT
TGCCGTATTCGAATTCGTATGGAAGTCCAGAAAAAATAGCAGTCGAAGAGA
GGATCATTAAATACCATACAAAGTATAGGAAATGA

>IR26
ATGACAATCAACTACGCCAGAGAGAGTGTCATAGACTTCACCAAACCCTT
CATGAATTTGGGCATTGGAATCCTGTTTAAGGTAACAAAA
>IR27
ATGGCATTTATATGGGACAGTAGCCGTTTGGAATTCGAAGCTGCGCAAGA
TTGTCAATTAGTCACGGCTGGCGAATTATTTGGACGTTCTGGATATGGAG
TTGGTCTACAAAAAGGATCTCCTTGGTCTGAAGCAGTTACATTATCAATT
TTAGACTTCCATGAGAGCGGTTTCATGGAAAACTTGGACGATAAGTGGAT
ATTTCAAGGGAGAGTAGAACAATGCGAAGATCAAGAAAAAACACCCAATA
CGTTAGGATTGAAAAATATGGCTGGTGTATTTATACTTGTCGCTGTGGGC
ATAGTGGTTGGAATGGTTTTAATCGTCATCGAGATAGGCTACAAGAAACA
TCATGTACGTAAACAGAATCGATTGCAGTTGGCCCGTAATTATGGACAAA
CGTGGAGAGCTATAGTTCAAAAACGAAAA
>IR28
ATGGCCTTTGAACTGGCCATACATAAAGTCAACTTGGATCCGGCACTTTC
AGACGATGTCAAACTCGAAGGCCGCATAGAAATCGTCGATATCAACGACG
GTTATCTAACTAGCAAAATAGTATGCGAATCCCTCGAGTCCGGTGTTGGA
GCTATTTTCGGTCCCGCAGGCTACGAGTCATCGGCCATAGTCCAATCGAT
ATGCGATTCTATGGAAGTACCCCACATCGAAACTCATTGGAAAATGAACC
CAAGACAACAACCGAACTATTACATAAACGTTTATCCGGATCCGGTAGTC
CTCAGTCGCGGTTATACGGCCATCGTCCGCGACATGGACTGGACATCCTT
TACTTTGCTATACCAGCGAGACGAAGGACTGTTGAGGTTGCAGCACTTAA
TCCAAGATTATTCTGGACTGACTAAACTATCAGATACTGAATCATCGGCC
ATTAGTATCATAAAGTTACCCGAAAATAACGATTTCCGACCTATGCTCAA
AGAGGTCAAAAAATCTCTGGAGAGTCATATTGTTCTAGATTGTGATACAG
ATATCATTTTAACTGTATTGGAGCAAGCTGAAGACGTAGGCCTAATGGAC
GATTATCACAGTTTTATTATTACTTCTCTGGATGCGCACACCATAAACTA
TGGTCACTTACAATTTAAACGTACCAATATCACTGCAGTAAAATTAATAG
ATCCGTCAAGTCCAACAGTGACTAGTATAATGGCAGATCTTGAATTCGTT
CAACAGAGAATGAATTTGAATATGGAAGTATTTCGAGCAGACACTATTAC
AGTTAACGCGTTATTGATGTTTGATGCGGTCAATGTGTACGCTAAAGCTT
TGCGTGGTATTGGTGGCACAAAAGCTGTCAAGGCCGAACCGAACAGTTGT
ACAAATAGATCGAGTACGGGATGGTCCAGTGGATTTAGTCTTATTAACTT
CATGAGAGTCGTAGAAATTGATGGTCTCACTGGAAAATTACGATTCGACC
AAAATTCTGGGTATAGGAGTTATTTCACATTAGAAATGGTTGAGTTGGCA

AATACAGGTTTCAAAAAGATCGGCGTTTGGGATCCACTAAACGAAATGTC
CTACACGAGAACTAGGAATCAGATGCTGGACGATCTAGTAAACGCAAACA
TGAACAAGACTTTCATTGTTGCTTCAAAAATTACGGAGCCGTACATGATG
TTGAAAGAAGATCATAAAAATCGAGTTGGCAACGACAAGTACGAGGGATA
CGTGGTAGATTTGATACATATGATTTCGGAAGAGATCAACATCACGTACG
AGTTTAAATTACGGAACGACGGTAATGGCAAGAAAGACAAGAAGACTGGT
AAATGGGACGGACTTATCGGAGAGGTTCATGAACTGAGAGCCGACCTAGC
TGTTTGCGATCTAACCATCACACACGACAGGAGAACGGCAGTTGATTTTA
CGACTCCTTTCATGAATTTAGGAATTAGCATCTTGTTCAGTAAACCGAAA
GAACCAGAAACAAATTTATTCTCTTTCACCCAACCCTTATCGTTTCACGT
GTGGATATATACTGCGACTGCATACTTGGGTCTATCAATTATTCTTTATA
TATTAGCTAGGATTACTCCTAACGAGTGGCAAAATCCTCATCCATGTGCA
ACGGAGCCCGAAGAACTTGAAAACTCGTTGTCTTTAATAAATTGTTTATG
GTTTTCTTTGGGGTCCATATTATGCCAG

>IR29
ATGAACGTGATCTTAGGTTTATTGGTGCCGTTCGTTTTGATTTTGGCCGG
TGGTAAACGATGCGGCGGCGACACGACGATCACGCTGGGGATTTTATACA
ACGAGGAAAACTCGATGCTGGAAACGGCCTTTAAGTCTTCCGTGGACGTC
GCAAAGGCCAAAATGCTTGCCAGCGGTGTTCAGTTGGACGTCATCAGCAA
AATAGTTCCTCTTTACGATTCGTTCGAGACCCAGCACCATGTATGTGAAA
TGCTCACCGAGGGGGTGTCCGGCATGTTCGGCCCGTCAGCCGGAGACACA
GCTCCCATCGTGCAATCGATTTGCGATTACAAGGAGATTCCTCACATACA
GACCCGATGGGATATCAACCAGAAACGAGGGTCTTGTCAAATTAATCTGT
ATCCT

>GR1
ATGAAAAGAATAATTCGAATTTGTATATTTCTTTTTTTTTCTTTTTTGCC
ATTTTTCTTCCTTTTCAGGAAATGCATCTCCCTCTGTCACATGCTGAACA
AGCTCTTTGCCCTTCAGTCATTCTTCTCCATCGCGCTCTCCTTCTTCCTG
GTTATCACCTTCCTGTTTCTGGGCTCACTCCTCCTCATCCAGAAAGCACG
AGGCGCTGTGAACAACTTTGAGTTTTGCCTGCTTCTAGCTGATGTACTGA
TCATGATTTTCATACATCTGCTGAAGCTATTGTACCTCATCTCCGTCTGC
AACAATACAAAATTACGGGCTGACAGAATTCTGCAACTTGGAAGAAAAGT
GATTATCGAGGATAAGTCCCCCGAGTTAAACTATGAGCTGACAATTCTGG
TGCAAATTCTGAAGGAGGAGAAGGTTGTGTTTACCGCTCATGATTTCTTC
CCTATCGATTATCCCCTGTTCATTTCGTTTATTGGGGCTGCTTCTACCTA
CTGGGTCATCATGGTTCAGTTCCAACTCACACCCTCTGAATAA
>GR2
ATGAGTACAACCGCCTTGGTATACCAGATGTTGATGACAAGCCACCGCAT
GGTTGATGCGATACCTCTCACGTTATACTTGAGACAGAGGTTGAGTCAGC

CAATCCAATTCAGCGCCTACGGATTTTTCCAGTTGGATTTTGCTCTGCTG
TATACATTTATTGGTGGCGTATCCATGTACGTTATTGTGTTCTTACAGTT
TCGTGTTTCCAGCCAAGACTAA
>GR3
ATGCAGGAGGCGACTCGCGTCATTGTGATCAGCTTGGCGGCCAGTGCAAC
TGCTGATGAGGTAAATAGAACGCTGTGGTATGTGAGTTCAGTGGATGCAT
ACAGTCTCGATTTGCGCATTCAGCATCAGGTTAAGTGTTTCTCTCTACAT
TTGCAACATCGAAGAATTAAGTTCACACTATGTCAAATGTTCACTATCAA
CACAACCTTCCTAGCTGAGATGGCGGTAGGAATAGTCAACTCACTCATGT
TGTCCTTACAGTACAAATACAGTGACAAACAAAGTCTGTCCAGCACGAGC
TAA

>GR4
ATGCAGTATTGCGTCCGCTTTGTCAGTATGATACAGATCTGTAATGTGAC
CAGCTCCGAGGCAAGGAAAGCGCGATCTCTCATTGCAAACATTAGTAACA
GATATCTCGATGTTGCTACTAAAGAAGAGCTGACGTTATTTTCAAATCAC
ATCTCAAGCAGGAATTTGGAGTTTACTGCGGGAGGATTTTTCAGTCTCAA
CACACATCTCATCACCTCGGCTATAGCTGCTGGAACAACATATCTGGTGA
TTTTGGTTCAATTCAATACTTCATGA

>GR5
ATGACGAGGAAGAAAATCAAAGTGATGATGGGTTTGAGTGGAACAAATCG
GAATAATATTGATACTATGATGGAAATATTTATACAACGCTTGAGCTATC
ACAAGATGAAACTAATGTTATGTGGCTTGTTTCCTCTCGACTACAGACTC
ATCTATTCGATTATAGGTACAGCTACTACATACTGGATCATATTGATACA
ATTTCAACTAGCTGTTTACGACAAGGAAGGTGTTCAGTAG

>GR6
ATGACTCATCAGTTGAGGAGACACCGCGTCTTGTTCACATCGTACGGAAT
ATTCGCGTTGGATTACTCTTTGCTCTACTCGATCATTGGAACAATTACAA
CTTACTGGGCCATTTTTGTCCAACTTCAATTATCTTCATAA

>GR7
ATGGAAGATTTCATGTTCTACACCAAGTCCATCCTGGTCTATCTCCTGTT
CATCCAGCTGGCTACTAAATGGGCCCGTCTGATGCGAGCTTGGGCCCAGT
TGGAGTGGAACATGCGGGCCTATGGATATCCACAGGACTTGGCTGTCCGA
TGCAAACTGGTCACTGTTCTGATGGTGTTTCTTGCTATAGTGGAACATCT
CTGGTTCGTAATCACCCGAATAGCCGACGCCAGTCAATGCACAAGACGGA
TGCCCCGCATGTCCCTCGCCGACGCGTACTTCCTCACCGTGTACCACCAG
CTGTTCTTCGTCGTACCCTACTGGCTACCGTTGGGGCTCTTCCTCGCCAT
CGTGGGCACCTACTTCGCCTGTGCCTGGAGCTTCGTGGACCTGTTCATCA
TCATGCTGTCCCACGCCCTGGCGTTGAGATTCGAGCAGATCAATCATAAA
CTGATGAGCTTGCAAGGAAAAGTGTTACCTAGCAGTATGTGGAGACAACT
TCGAGAAAGCTACAACGAGTTATCTTGTTTAACCAAACTGCTGGACCAGA

CATTGTCACCCATAGTGTTACTGTCATTTGCAAACAATTTGTATTTCATA
AGCTTACAACTATTCAACAGTCTCAAGCCAATGCAAAATGTTTGGGAGGC
CATCTATTTCGTCTACTCGTTCACCTATCTGCTGGTGCGGATCTGCGCAG
TCTCACTCTACGCAGCATCCGTCAATGATGCCAGTAAAGAATGTATGGGT
GTCCTCTTCTCAATACCATCAGAAAGTTATTCTGTGGAGGTATCCCGTTT
CCTAACCCAGGTGACCTCCGATGATCTGGCCCTGACCGGCTGCAAGTTCT
TCTCCGTGACTCGAACCCTCATGCTGACGATTGCGGGCACCATCGTCACC
TACGAGATTGTGCTCGTGCAGTTCAACGCGGTGGCCGGCGACTCCAACCC
GGACAATACCACGCGACCTTGCCGATACATTACGGTCGACATGTGA

>SNMP1
ATGCCTCCGGGAGCAGCCTTCATTATCGGAGGAGCGATCCTTTTAATGTT
TGGACTTTGGTTCGGATGGTTTGGATTCAAAGGGATTTTGAAAAGTCAAA
TTGGAAAGCAAGTTCGACTGGTGGAAGGATCTGAAATGAGAGCACTTTGG
TCCAAACTGCCCATCGCTATTTATGACGACATATATTTCTTCAACGTCAC
AAATTCAGAGATGGTCTACCAAGGAGAGAAGCCAAATCTGCAGCAAGTGG
GACCGTACTGTCTCGAGTAG

>SNMP2
ATGAGACTTCAGTTCAACATGTTCATGTATGAGATGAAGAAAGTCAGCAT
CACCAAGAACTTGAGCTCCACCCCCATGTTACATCCCTTGTTCTGGCTGC
AGTCAGTAAGTAATGGAAACTAG
>SNMP3
ATGAATCCCAACCGGACTTTGCATGAATTCAAAATAATTTTGGAAAACGA
TTATTCAATACCGCTCAGAGTAGATGCTAGAATTCAATTTAATGTCAAAG
TGAAACCAATCAAGGGATTAAAAATTTTAAAAAACATGCCAGAAATCTAT
TTACCTGTGTTTTGGTTTTCTGAAAGTTTTGAAATTCCACGCAATATGTC
TGACCAATTGTTGATTGTTACAAACGTATTACCAATGTTTGTTCCTTACG
TATGGCTTATATTGGCATTAGCCGGATGTATAATGTTAGTCTTTAGCACT
TACTTATGGGTGAGCAGAAAAAATGAATCCTTCACCATCTTGGACCCCTT
GTGA
>CSP10
ATGTTGAAGCTTTGCTGCGTTGCCGTTGGGCTGTTGTTCGTCGTTGAGTT
CAGCAATGCCGCCGCGGTCGGTAACGCCAAAGAGGTCAAGTACACCACCA
AGTACGACAACATTGACATCGACCAGATCCTGGCCAATGAACGACTCTTC
ATGAACTACTACAACTGTCTGCTGGAGAAGGGCAAGTGCTCACCTGACGG
CCAGGAACTGAAAACTCTGCTTCCCGACGCCATTGCCACCGAATGCGTGA
AATGTTCCGAGAAGCAGAAGGAAGGATCCAAGAAGATCTTCAAGTTCCTG
ATCGAGAAGAAGCCAGCTCAGTGGTCCGAGCTGGAAGCCAAGTACGACAC
CGAAGGCACCTACAAGAAGAAGTACGAGAAGGAGCTGGCCGATCTGAAAG
CCGGCAAGCCCGTGAAGATCTAA
>CSP11
ATGGCCCGTCTACTCTCATTCGCTCTACTGTTCGTGTGCTCAGCGAGCCT
CGCTCTTGGCCAGAAAGCGGCCAAGGAAACTGCGGCCAGCGAGTCCGGCA

TTCTGGACAACTTCGACGTGGACACGGTGTTGAATAACGGCCGCGTTCTC
AAGAACTATGTCAAGTGCGTGCTCGGCAACGGGCCTTGCACGGCCGAGGG
ACGAGAAATGAAAAGAGTTCTCCCCGATGTCCTGAAGACAGCCTGTGGCA
AGTGCTCCGAGCAACACAAGGAGAGGCTGCGCAAGGTCCTCCTCAAGCTG
AAGAACGACCCCAAACTGAAGGACGACTACAAGAAAGTCATCGAAAAGTA
CGACCCCAAGATGCAGTACGTCGCCAACCTGGAAAAATTTTTACTCAGTT
GA

>CSP12
ATGTTCAAGTCTCTGGTTCTGGTGGCGTGTTTAGCCTGCGTCGGGTCAGT
GCTGTCAGCTCCTGCCTACACCACCAAATACGACAACGTGGATCTCGATG
AAATCATCTCCAACGATCGTCTCTTCTCCAACTACTACAAGTGTTTGCTG
GACCAGGGCAACTGCTCGCCCGATGGATCTGAATTGAAGAAAATCCTGCC
CGATGCAATTTCTAACGAATGCAAGGATTGCTCTGAGAAACAGAAGGAAG
GCTCCAAGAAGATCTTCAAGTTCCTCATTGAGAAGAAGCCCGAACAGTGG
AAAGCTCTGGAAGAGAAGTACGACCCCAACGGAACCTACAAAGTCAAGTA
TGACGCTGATCTCAAGGCTTTGACAGCCGAATAA
>CSP13
ATGAAGCTTTCATCCACCACATTCTGTCTCTGTTCGTCCCTACTCATCGT
TGCAGTTCATGCGGCGGTCACGTCCAAACCGTCCACAGCAGCATCTGACC
CAAAGCCCCTCGCTGACGCAAAGACCTCCACTGACGCAAAATCTGTGACA
TCATCCACGTCCAGTTCTTCTTCGTCAAGCAAATCATCTGCTCCAGGAGC
TGTCCCTTCAGTCAACAAGGCAGCATCCAAAGCTCCTCCCAAGTCAGCGA
CTGCTGCACCAACCAAGGCGAGTGCGGTGTCAGATGATGAACTGGACAAG
TTGCTGCTCGACAGACGGTACCTGTCAAGGCAGCTCAAGTGTGCGCTGGG
CGAGGGAGTGTGCGACCCCGTGGGTAGGAGGCTGAAATCTTTCGCCCCCC
TGGTCCTGAGAGGTAACTGCCCCCAGTGCACCCCGGCCGAGACCCGACAG
ATCCAGAAAGTTCTCTCCCACGTTCAACGAAACTACCCGAAGGAGTGGGC
CAAGATCATTCAGCAGTTCACGGGCAATCGGAAATGA
>CSP14
ATGTTCAAGTCTCTGGTTCTGGTGGCGTGTTTAGCCTGCGTTGGGTCCGT
GCTGTCAGCTCCTGCCTACACCACCAAATACGACAACGTGGATCTCGATG
AAATCATCTCCAACGATCGTCTCTTCTCCAACTACTACAAGTGTTTGCTG
GACCAGGGCAACTGTTCACCCGATGGATCTGAATTGAAGAAGATTCTACC
TGATGCCATTGCCTCCGAATGCGGTGGCTGCTCTGACAAACAGAAAGAAG
GCTCCAAGAAGATCTTCAAGTTCCTCATTGAGAAGAAGCCAGAACAGTGG
AAAGAATTGGAAGCCAAATACGATCCATCCGGTGCCTACAAAGCCAAATA
TGACGCAACCCTCAAAGCTTTATAA
>CSP15
ATGTACAAAGTTTTAGCTTTCGCCGTCTGTTGTGCAGTGATCGTCAGCTG
TCTGGCGAAACCCCAGGACAAGGAGAAGAAGTACACCACCAAGTATGACA
ACATTGATCTGGAAGAGATCCTGAACAATGAGAGACTCCTCAAGAACTAC
TACAACTGTTTGATGGATGAGAGCCCATGCACACCTGATGGTGGTGAACT
CAAGAGTAAGTTATTTCAATTGTTTAAATTTTTAAATGAATTTCAACCAA

GTCAAATTGTATGGAACTTAAAATAA
>CSP16
ATGCGTTCGTCGACAAACTATCGTGTGTGCGTGCTTTCTGCGGTGATTCT
AGTTGCGGCCCTGCGGCGGGGGGTGTACGCTAGGCCGCAGAATACCATCC
AGTCGTCCACCGTGAAGGATGAGTTGCCCGCTTACCCGACAAGGTACGAC
TCCATAGATGTTGACCTGATCCTGTCCAACGACAGAATCATCCGGAGGTT
CATCGACTGCATCCTGGCCCGAGGTCCTTGCACGAGGGAGGGACTGGAGC
TGAAAAGAATCATCCCGGACGCCATCCGCACAGAGTGCGCCAAGTGCAAC
CCGTCTCAGAAGAAACACGTGGGCAAGGTCCTCTCCTATCTGTTTCACAA
CCGCAAGAACTACTGGGATGAGCTGCTCGCAAAGTTTGACCCGGAGAAGA
AGCTGAGGGAGAAGTATGGCTTCACGTAG
>CSP17
ATGCGTTCGTCGACAAACTATCGTGTGTGCGTGCTTTCTGCGGTGATTCT
AGTTGCGGCCCTGCGGCGGGGGGTGTACGCTAGGCCGCAGAATACCATCC
AGTCGTCCACCGTGAAGGATGAGTTGCCCGCTTACCCGACAAGGTACGAC
TCCATAGATGTTGACCTGATCCTGTCCAACGACAGAATCATCCGGAGGTT
CATCGACTGCATCCTGGCCCGAGGTCCTTGCACGAGGGAGGGACTGGAGC
TGAAAAGAATCATCCCGGACGCCATCCGCACAGAATGCGCCAAGTGCAAC
CCGTCTCAGAAGAAACACGTGGGCAAGGTCCTCTCCTATCTGTTTCACAA
CCGCAAGAACTACTGGGATGAGCTGCTCGCAAAGTTTGACCCGGAGAAGA
AGCTGAGGGAGAAGTATGGCTTCACGTAG
>CSP18
ATGTTCCATATAGACAGCATAATGAAGAGCTGGGTACTGATCTCACTGGC
GTTGACGTGCCTGGCAGGAGTGCTGACTGAAGAGGCTCCTGATCTGGAGA
AGAAATATGCCAACTTTGACATCGAGGCGGTACTGAAGAGTAAGAGACTG
GTGAACAACTATGTAAACTGTCTGATTGACAAGGGTCCCTGCAGCCCCGA
GGGAACTGATTTGAAGAAAACCCTCCCGTCCACCCTCCAGAGTCTGTGTG
AGAAGTGCACTCCCAGCCAGGTAGACAAGACCATCAGCGTCATCAAGCGG
GTGAAGAAGGACTACCCCGAGGAGTGGAAGGTCTTGCTGCAGAAGTGGGA
TCCCACGGGAGAGTACAAGAAGAAGTTTGAGGCCAAGTATGGCAAGAAGC
TGGACGAGTAA
>OR26
ATGGTCGCCAGCCCCACTAAGCTCACCCCATTACGCTACTTCAAATTCGC
AGCCGAACTGATAGCCGCCGTTATCGAGTACTTTATCTTGTGCAACTGCT
CGGAGGTCTCCGCGGAATGTCACGAACTAATCCGTACGGCACTCGGTAAT
TCCCGGTGGCGCTCGTGTACTCGAGAAACCCGAAAGGATTTGGTCATGTT
ACTCAATCGTGTGACGAAGGAAAATCATGCGCGCTTCGCTCATGGCGCGA
TAGTTTTAAATCGGCCTTTTATTCTGAATGTCATGAGAGTGGCCTATACG
TTTGTAAACTTCATGAGGCTGAGGAGTGTTCCTAAATAA
>IR30
ATGTATAAATGGTATCTACTTTTAAACTTTGTAATTTTAAGTTTCAACAA
TGCGAGTGGATTTCTGCCCTACCAGATCGATCAGCGAGAACCCATAAAAA
TATTGGGATTATTCAGTCCAAATGAAGAAGACCTAGCAACGGCTTTCAAA
ATAGCAGTCAGAAGAGTCAATAAAGATGTTAAACTTCTGCCCAGAGAAAT

TGTTTTCGACCCCATTGTCGAGTTTGTCGAAAGCTATGACAGTTTGACTA
CGGGAAAAATAGTCTGCAATGCCACACAGGAAGGAATCGCCGCAATTTTC
GGACCGCAATCCAATGAAAACCGCAACATCATCGAGTCCATGTGTCAGAT
ATTCGACATCCCGCATGTCGAGGCATTCTGGGACTCGAACACCTATTCCA
TCCCTACCAACGCTGTGCATGGCGTCAATGTGTTCCCGGAGTACCACCTC
ATATCCCAGGGAATCAGTGACATAATCAAGGACATGGACTGGAGTACTTT
CACCATCATCTACGAGAACCACGAGAATCTGGTCTATCTGCAAGAGGTGA
TGCTAAATGCCATCGAGGATGACGACAAGATCAAGCCTGGAAAGCCCGTT
GTTACCGTCAAGCAGCTACCTCAAGATACCAGCGACTTTAGACCCTTGCT
GAAGGAGATTAAGAATTCCTCAGAATCTTTCATCCTGTTAGACTGCTCGC
TGGACAAGATCGTTCCAATTCTCAAACAAGCGAGAGAGGTTCACTTGATG
GGGGACTACCAGAACTACATTCTTACTAACTTGAATGCCCATACAGTGGA
TTTCGAGGATTTCTCTCCGGGCTACGCCAACATCACAACGGTCCGAATGA
TCAATCCCCAGTCTCCCGTCATACGCAGCATCATGAGCGGGTGGATGTAC
GAGGAGAATGAGAGGGGAAGATCTCTGAACGTGAAGGCCGAAACTGTGAC
GATTGAAGCAGCATTGATGTATGATGCAGTATATCTGTTTGCGACCGCCC
TAACAGCTCTTGGCGAAAGCAAACCACTGCCTGCCAAGCTGAGTTGTGAC
ACGCCAAGTCCTTGGAAGCATGGCTTGGGGATTGGGAACCTCATGAAATC
GATCACAACCGAAGGAATGAGCGGTCAAATCAAACTGGACAGTGTGACGG
GGAGAAGAAACTCGTTCTCGCTGGAGTTTGTCGAATTTGTGGGCGGACAG
TGGAAAGTTCTCGGAACCTGGAACACCGCGTTCGGACTGAACCACTCGCG
GACCGTGGAGCAGATGGACCGGGAGAAGAAGGAGAAGATCGAGAATCGAA
CCCTGACTGTAGTGTCAAAAATAGGCGAACCCTATCTCATGAAGTCTTCC
AAAGGGGAGCTCTATGGCTATTCTATAGACTTGATCGACATGATTGCCAA
GGAACTGAATTTCAGCTACCAGTTTGTCCTCGAACGTGAAAATAAGTACG
GGACAAAGGATCCCAACACAGGCAAATGGGACGGACTCATCCGGGAGCTG
CAAGAACAGAGAGCTGACTTGGCCATATGTGATCTCACTATTACATCTGA
GAGAAGGAGTGCTGTCGACTTCACAATGCCATTTATGACATTAGGTATCA
GTATTCTCTATCGAACACCTCCTAAAAAGCAGCCAGACCTTTTCTCTTTC
CTCGAACCCTTATCCTTCGATGTTTGGGTCTACATGGCCACAGCTTACTT
GGGAGTGTCGCTTCTTCTCTTCTTCCTAGCAAGGTGCACCCCATACGAAT
GGGAAAGCCAGCATCCCTGTGATTCCGACCCCGAAGAATTAGAAAATGTT
TTGAATCTTGTCAACTGTCTCTGGTTTTCCCTAGGATCAGTTCTGGCCCA
AGGTTGCGATATTCTCCCAAAAGCCGTGTCGACTAGAATGGTGGCCGGAA
TGTGGTGGTTCTTCACCCTCATCATGATATCTTCGTACACCGCCAATTTG
GCTGCATTTCTCACGAACACCCGAATGAATCCGCCCATCAAAAACGTGGA
AGAGCTGGCGAAAGCGGGCATGATCAAGTACGGCTGCGTGGAAATGGGAT
CCACGCGAAACTTCTTCAAGAACTCAAACGTCTCCCTGTACCAGAGGATG
AACAGCGCCATGGAGTCTGCACGGCCCTCTGTCTTCGTGAAGAGCAACAA
GGAGGGGGTGGACCGGGTGTTGAAGGAGAAGGGCAAATACGCCTTTTTCA
TGGAGTCGACGGGAATCGAATACGAGGTCGAGAAAAATTGCGAGCTGATG
AAAGTTGGTGGACTCCTGGATTCGAAGGGATATGGAATCGCAATGCCCTT
CAATTCTCCTCACCGGTCGACAGTGAGTGGAGCAGTGCTGAGATTGCAGG
AGTCCGGCAAGTTGAGAGAGCTGAGAAACAAACACTGGGAGGAACACAAC

CAAACACAGGACTGTGAGGAGGATGAAGGCGATTCCGACAATCCAGAGCT
GGGCATAGCAAACGTCGGTGGTGTGTTTGTGGTTCTAATCGCCGGCTGCG
GGGTGGCCTTCCTATTCTCCCTTCTGGAATTTCTCTGGAATGTGCGCAAA
GTTGCCGTGGAAGAGAAGCTGTCTCCGTACGAAGCATTCATGTTGGAGCT
GAAATTTGCCGTCAGTTGTCATGGTACTTCGAAGCCAGTGAGAAGAAAGA
ATTCCATCGCGACATTCAAAGGGGCTAACTCTATTCCAAATCTTGAGCTG
TTGAACTCTAGAATTTATAGTGATTTCAAAAACCACAATAATACATAA
>IR31
ATGACCGCAGAGAGGGAGGAGGTGGTAGACTTTGTGACGCCCTACTTTGA
TCAAACTGGCATCACAATTGTGATTCGCAAACCCATTCCCAAGACCTCCC
TGTTCAAGTTTATGACCGTGCTCAAGTCTGAGGTTTGGCTTTCCATCGTG
GCTGCTCTGGTAGTCACCGGTATCATGATATGGCTATTGGACACCTACTC
ACCTTACAGTGCGCAGAACAATCCACAACTCTATCCACCATCTACCAAAA
TCTTCACTCTCAAGGAGAGCATCTGGTTCGCCTTGACATCTTTCACCCCT
CAAGGAGGAGGAGAAGCGCCCAAAGCTCTAAGTGGGCGCACTCTGGTCGC
ATCCTATTGGCTGTTTGTGGTACTTATGCTTGCAACGTTTACTGCTAACC
TAGCAGCATTCCTAACTGTGGAGAGAATGCAGTCCCCAGTTCAGTCCCTG
GAACAATTAGCGCACCAGTCCCGCATCAACTACACTGTAGTGGACAACTC
TAACGCGCACGAGTACTTCAAGAACATGAAACACGCTGAGGATATTATTT
ACAGAGTTTGGAAAGAAATTACATTGAATGCTTCGTATGATCAGAAGCAG
TTTCGTGTGTGGGACTACCCAGTCAAGGAGCGATACGGTCGAATACTAGA
GGCTATCGAGAAAACGGGACCCGTGCAAGATGTCAAAACTGGTTTCGACA
AGGTTCTGGAGAGTGAGCAGGGAGAGTTTGCCCTCATTCATGATTCTGCC
GAAATCAAGTACGAGGTGTCCAAGAATTGCAACTTGACGGAGGTGGGAGA
ACTTTTTGCCGAGCAACCCTACTCCATAGCCGTTCAGCAAGGCAGTGAGC
TCGCTATTGAAATAAGCTCTGTTATTTTAGATTTACAGTCCGACAGATAT
TTTGAATTTCTAGACTCGAAATATTGGAACACGACTATGCAGAATAAACA
ATGCTCCAATGAGGACGAGTCAGAGGGTATAACACTGGAAAGTTTGGGTG
GTGTATTCATAGCAACTCTATTCGGGCTAGCCCTTGCCATGGTTACTCTA
GTGCTGGAAATCTTCTATCACCAGAAAAATAGGGCCCGAAACAATCGTCA
CTTTGGAAAATTAAAACAAACTGCCGCTCCCATGCAGCGTCAAACATTTG
AAATTCAAAAGTCTTTTTTTCGCCCTTACAAACCAATTTTCGACTCCTTC
AGACGCAAGAAAACCGTTCACTCTATATCAAATATAACAAATGTGCGCGA
GAAAGATAATCTAAATATCCTGAAAAGTCATTCTAAAATGGCAACCATTC
AGGAGAAGGAGATGGATAGCTTTCCGGACGCTTTCCTCTATGCTGGTGAC
TTTGGAGGGGTACATCGGAAGCTGGAGATGAGGAAAAAGCCAAGGATGAT
CTCCGTGCTCCCATCCGACGGCTGGTATTGA
>IR32
ATGTTTCTCTATCTGGTCTGTCTTGTGCTAGTAGTGGCTGTCCCGTGCGT
GCCGAGCCTGCCTCCTATCATCAAAATAGGCGCGATATTCTCAGAAGCTG
AGAGGGGGGGAGGCTCCGAGCTTGCATTCCGCTACGCCATTCACAGGATT
AACAGAGACAAGTATCTGCTTCCCCACACACAGCTCGTGTACGACATCCA
GTATGTGCCCCTGGATGACAGCTTCCATGCGTCAAAGAAAGCCTGTCAAC
AAGTGCAGTTCGGGGTGGCTGCCATGTTCGGTCCCTCCGACCCTCATCTG

GGCCCCCACATCCACTCCGTGTGCGACGCTTTGGACATCCCTCACATAGA
GGCACGTCTAGATCTGGACGAGGACTATAAGGAGTTCTCCATCAACCTGT
ATCCTGCGCCGAGAATACTGAACAGGGCCTATCAGGATGTTATCCGCTAT
CTCAACTGGACCAAACTGGCCATTGTCTACGAGACGGACTATGGTTTGAT
AAAGCTGCGGGATCTAGATAAGACCCCGAGCAACAAGGATCTCGAGATTC
ATCTGATGCAAGCTGACCCTGGCACTTATCGCGATATACTGAAGACCATC
AAGACGAAAGAGATACAGAATCTGATAGTGGACACAAAGCCAGAGCATAT
TAATGAGTTCCTGAAAGGGATTTTGCAACTACAAATGAATGATTACAAGT
TCCACTATTTGTTCACGTCTTTTGACATCGAAAGTTTCGACTTGGAAGAC
TTCAAGTACAATTTTGTGAACATGACGGCTTTCCGCATTGTGGACGCCAG
TGACATTACCATCCGAGAGATTCTGCGGGACATGGACCGGTTTCGAAACG
GGGGCAAAACCTCACACAACATGACACTGGTGATTTCGTCGGACGCCGCT
CTTATGTACGATTCTGTTTACGTGTTCGCGGTGGGACTCCAAACCTTAGA
GCAGAGCCACTCCCTGCAGCTGTCTAACCTGTCCTGCGAAGAGGAGACAC
CCTGGGACGCCGGACTTAGCCTCATCAACTATGTCAATTCGGTCGAAATG
AAGGGTCTCACGGGGCCCATAGAGTTTAAGGAGGGGCGGCGCGTGGATTT
CAGGCTGGACCTTCTCAAGCTGAAGCAACACTCCCTGGTTAAGGTGGGGG
AGTGGGAGACGGGGGAGGGGGTCAACATTAGCGACCCCGGCGCCTTCTTC
GACTCGAGCACCATGAACGTGACCTTGGTGGTCGTCACCATACTCGAAAC
CCCGTACGTCATGCACAGAGCTTCCTCCATGAATTTGACGGGCAATGCCC
GCTACGAGGGTTTCTGCATCGATCTCTTGCGGGCCATTGCCACGATGGTG
AACTTTGAGTACAGCATCCAACTTGTGCCCGACGGGAAGTATGGCGTGTT
TGATTATGAGAGCGGCGAGTGGAATGGGATAGTACGTCAGCTGATGGACA
AGAAAGCAGATTTAGCTGTCGGCTCTATGACCATCAACTACGCCAGGGAG
AGTGTCATAGACTTCACGAAACCCTTCATGAATCTTGGAATCAGCATTCT
GTTCAAGGTTCCTACCTCGGCCTCGACGCGTCTTTTCTCCTTCATGAACC
CGCTGGCCGTGGACATCTGGCTATATGTCCTGGCCGCCTACATTCTGGTC
TCCCTCACCATGTTCCTTGTGGCGCGGTTCTCGCCCTACGAGTGGCACAA
CCCGCATCCCTGCGACCTCAGCAACGACGAAGTCAGGAACCAGTTCTCCA
TGGCCAACAGCTTCTGGTTCACGATCGGGACGCTCATGCAGCAGGGCTCG
TTGAACCCCAAGGCCACCTCTACTCGTATCGTGGGTGGAATTTGGTGGTT
CTTCACTCTCATCATCATCTCCTCTTACACCGCTAATCTAGCAGCATTCC
TCACGGTGGAGCGAATGATCACGCCCATCGAGAATGCCGAAGATCTGGCG
GGACAGACGGAGATAGCGTACGGGACGCTGGAGAGCGGATCTACCATGAC
TTTCTTCCGGGACTCCATGATAGACACGTACAAGAAGATGTGGAGATACA
TGGAAAACAAGAAGCCATCTGTGTTCGTGCCTACCTACAAAGAGGGAATC
GATCGCGTCCTGGAAGGTAACTACGCGTTTCTCATGGAGTCAACTATGCT
AGACTACATTGTACATCGGAACTGTAACCTCACGCAGATTGGGGGACTGC
TGGATTCAAAGGGCTACGGCATTGCGACTCCTATGGGGTCCCCGTGGCGG
GACAAGATATCCCTGGCGATTCTCGAGCTGCAAGAGAAGGGAGAGATCCA
GATGATGTACGACAAGTGGTGGAAGCACAACAGGGGAGACATGGAGTCGT
GCAGCAGACACGACAAGAACAACAAGGAGAGCAAAGCCAACGCTCTGGAT
GTTGATAATATAGGAGGCGTATTTGTCGTTCTGCTTTGTGGCCTCGCTAT
CGCCATTATGGTCGCCATCTTGGAATTCTGCTACAACAGCAAGAAGGCAT

TGCACTCGGATTGTGCCTCCCCCAATCCTCAGGCCCAGTCCCTGTGTTTC
GAGATGACGGACGAACTCTGTTTCGCCCTCAAGTGCCGAGGCTCGCGACA
GAAACCGGCCCTGCGTCGCAAGTGTTCCAACTGCAACGCCACCCGGGACT
TGGATCTCGGACTAGACCTGCCACCACCACCTCCGCCCCTGACTAGGCTA
CGAACCCAGCGAGAAAGTATATGTGATATCCCCCCACCCCCTGCCCCACT
GTACAGAACTACCATGCACCGCTATCTCAACGCTTCCCCAGACGACACCA
CCTAG

>GR8
ATGACCTTCGATATTAAAGAGGCGATAGTAAAAAAAAGGCTCATCTTTAC
TATCCTCCTTCCAGAGTTCATCCTCACCTACTCAGCAATGGCATGTACCT
TGGCCGCCAATAACCTCATCTCTCTCTGTCTACTCATCATGGAATACTCC
CACAAGGAGATCACTCGTCAGCTGAGTGCCCTCAGCAATGTGACTGTCAC
TCGCACAGTCCTCATCTCCACGCAAAACCTTCATCGGTTGCGTCTCCTCC
ACTGGCAGGTATAG
