# Supplementary material for: Transcriptome Screening and Identification of Chemosensory Genes in the Goji Berry Psyllid, Bactericera gobica (Hemiptera: Psyllidae)
Source: Biology (Basel). 2025 Aug 21;14(8):1105. doi: 10.3390/biology14081105 (PMC12383947; doi:10.3390/biology14081105)
Supplement: Supplementary file 1 [file biology-14-01105-s001.zip › biology-3758692-supplementary/Supplementary Files/Supplementary materials.pdf]

Supplementary materials

Table S1 Quality Table for the Transcriptome Unigenes of *Bactericera gobica*

sample	raw_base	raw_bases	clean_reads	clean_bases	error_rate	Q20	Q30	GC_pct	Depth	RIN
FA1	20610747	6.18G	20168122	6.05G	0.03	97.55	93.16	38.81	90.13	6.70
FA2	21990648	6.6G	21623159	6.49G	0.03	97.51	93.04	37.33	96.16	6.20
FA3	21832495	6.55G	21476607	6.44G	0.03	97.24	92.36	36.34	95.47	6.30
MA1	22505271	6.75G	22128447	6.64G	0.03	96.84	91.91	38.17	98.42	7.70
MA2	21763570	6.53G	21296761	6.39G	0.03	97.04	92.28	36.67	95.17	6.20
MA3	22506143	6.75G	22147448	6.64G	0.03	97.62	93.26	38.42	98.42	7.20
FH1	22043395	6.61G	21201343	6.36G	0.03	96.15	90.39	39	96.40	7.70
FH2	22917093	6.88G	21924367	6.58G	0.03	96.2	90.43	40.38	100.22	7.70
FH3	23602330	7.08G	22696898	6.81G	0.03	96.64	91.52	40.37	103.21	10.00
MH1	23876613	7.16G	22919990	6.88G	0.03	96.14	90.56	36.95	104.41	8.40
MH2	22625024	6.79G	21815233	6.54G	0.03	96.49	91.22	40.34	98.94	8.30
MH3	23342474	7.0G	22579374	6.77G	0.03	96.7	91.67	40.05	102.08	8.30
FB1	22654397	6.8G	21823227	6.55G	0.03	96.21	90.42	39.24	99.07	7.50
FB2	24592156	7.38G	23730526	7.12G	0.03	96.46	90.96	39.1	107.54	7.90
FB3	26736339	8.02G	25723015	7.72G	0.03	97	92.33	39.22	116.92	5.50
MB1	19980336	5.99G	19342897	5.8G	0.03	96.08	90.03	36.95	87.37	5.40
MB2	19339144	5.8G	18569345	5.57G	0.03	96.85	91.96	38.29	84.57	7.10
MB3	23805148	7.14G	23042789	6.91G	0.03	96.25	90.47	35.63	104.10	6.70
average	22595740	6.78G	21900530	6.57G	0.03	96.72	91.55	38.4	98.81	7.27

Table S2 Table of Frequency Distribution of Antenna Splicing Lengths

Unigene length interval	300-500bp	500-1kbp	1k-2kbp	>2kbp	Total
Number of transcripts	36839	30732	20855	19676	108102
Number of Unigenes	24244	20579	10779	8221	63823

Table S3 List of Distribution of Antenna Splicing Lengths

	Min_length	Mean_length	Median_length	Max_length	N50	N90	Total_nucleotides
Transcripts	301	1262	705	26407	2107	490	136454012
Genes	301	1061	615	26407	1612	440	67713994

Table S4 Whole-organism Gene Reads Statistics Results

name	Bases(G)	Reads	Read Length (mean)	Read N50
Polymerase Reads	87.93	730803	120320	201361
Subreads	84.89	41130546	2065	2377

Table S5 Whole-organism Gene Correction and Redundancy Removal Statistics Results

name	number	Min_length	Max_length	Mean_length	N50
CCS	680004	58	14988	2390	2622
FLNC	554682	58	14895	2295	2536
Polished consensus	39138	73	8706	2450	2711
After correct	39138	73	8706	2449	2710
transcripts	17290	73	8706	2626	2907

Table S6 Antennae Gene Annotation Statistics Table

Kind of Database	Number of Unigenes	Percentage (%)
Annotated in NR	22871	35.83
Annotated in NT	14588	22.85
Annotated in KO	11081	17.36
Annotated in SwissProt	15677	24.56
Annotated in PFAM	19123	29.96
Annotated in GO	19122	29.96
Annotated in KOG	9572	14.99
Annotated in all Databases	4336	6.79
Annotated in at least one Database	30216	47.34
Total Unigenes	63823	100

Table S11 Whole-organism RNA Quality Detection Table

Sample Name	Concentration (ng/ul)	Volume (ul)	RIN
Whole-organism	294	35.83	5.4

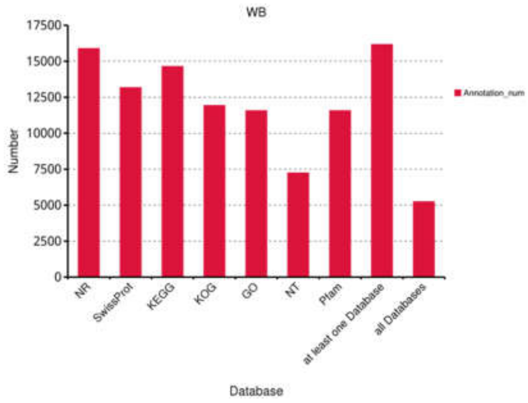

Figure S1 Statistical map of Whole-organism gene annotation results

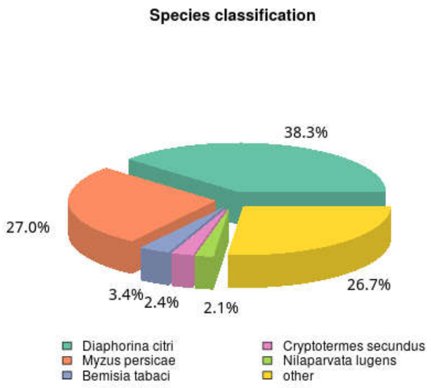

Figure S2 NR database alignment statistics chart for antennal genes

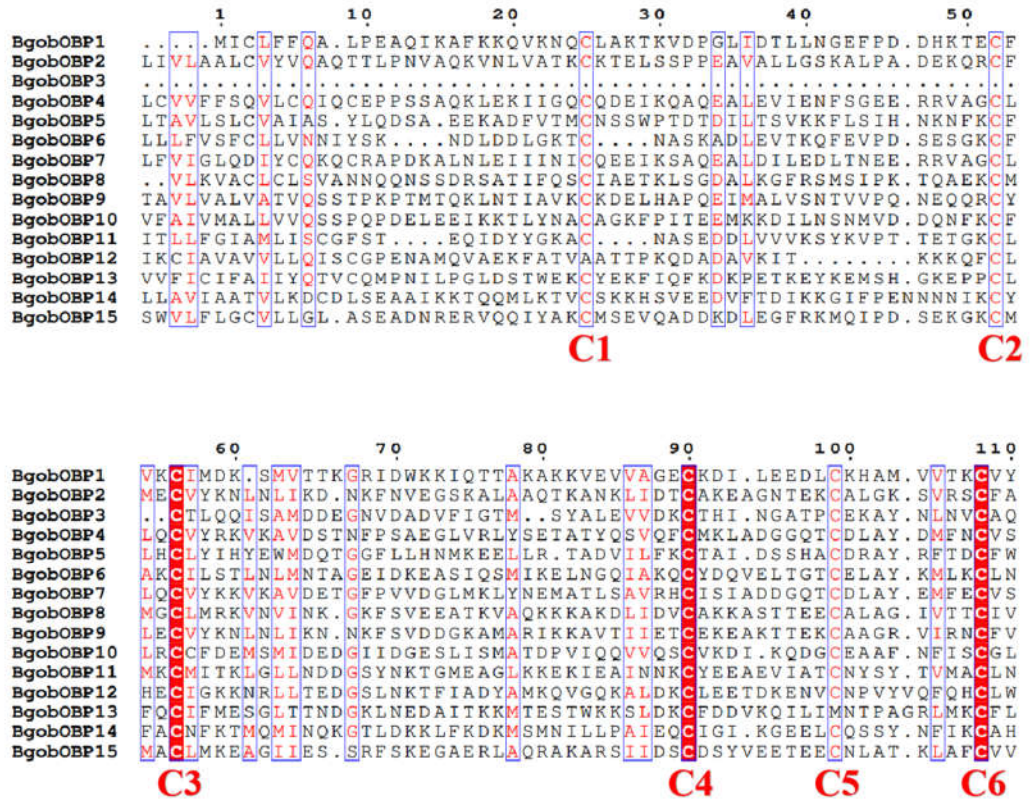

Figure S3 Amino acid multiple sequence comparison of OBPs of *Bactericera gobica*

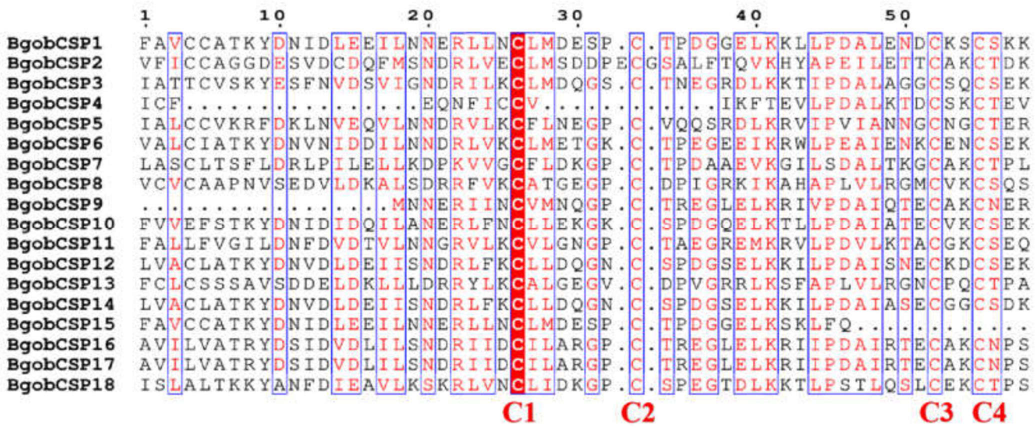

Figure S4 Amino acid multiple sequence comparison of CSPs of *Bactericera gobica*

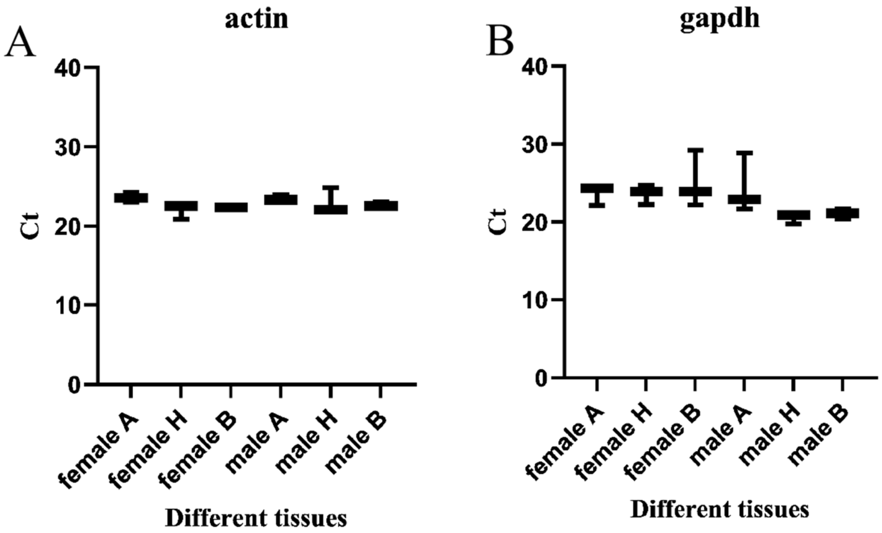

Figure S5 Differential expression of actin and gapdh in different tissue.

(There is no difference in the expression levels of actin and gapdh among different tissues ;one-way ANOVA, F=1.648, P=0.221 ;one-way ANOVA, F=1.855, P=0.177)
